# Oxidatively Induced Reductive N_2_ Binding: A Dinickel-Bridging Bent N_2_ Radical Anion and Its Redox-Triggered N_2_ Release

**DOI:** 10.1021/jacs.5c09334

**Published:** 2025-09-06

**Authors:** Sara I. Mozzi, Dennis-Helmut Manz, Nils Ostermann, Roland A. Schulz, Peng-Cheng Duan, Thomas Kothe, Martin Diefenbach, Sebastian Dechert, Serhiy Demeshko, Vera Krewald, Inke Siewert, Franc Meyer

**Affiliations:** † University of Göttingen, Institute of Inorganic Chemistry, Tammannstraße 4, D-37077 Göttingen, Germany; ‡ TU Darmstadt, Department of Chemistry, Quantum Chemistry, 64287 Darmstadt, Germany; § Universität Göttingen, International Center for Advanced Studies of Energy Conversion (ICASEC), Tammannstraße 4, D-37077 Göttingen, Germany

## Abstract

Nitrogenase accumulates reducing equivalents in hydrides and couples H_2_ elimination to the reductive binding of N_2_ at a di-iron edge of its FeMo cofactor (FeMoco). Here, we describe that oxidation of a pyrazolato-based dinickel­(II) dihydride complex K­[L­(Ni–H)_2_] (**1** ^**K**^), either electrochemically or chemically using H^+^ or ferrocenium, triggers H_2_ elimination and binding of N_2_ in a constrained and extremely bent bridging mode in [LNi_2_(μ_1,2_-N_2_)] (**3** ^**N2**^). Spectroscopic and computational evidence indicate that the electronic structure of **3** ^**N2**^ is best described as Ni^II^–(N_2_^•–^)–Ni^II^, with a rare 1e^–^ reduced and significantly activated N_2_ substrate (*ṽ*_~NN_ = 1894 cm^–1^). **3** ^**N2**^ is also formed upon 1e^–^ oxidation of K­[LNi_2_^I^] (**2** ^**K**^) under N_2_. This is an unusual and counterintuitive scenario where the oxidation of a dinickel­(II) dihydride, or of a dinickel­(I) complex, induces the reductive activation of N_2_. Detailed (spectro)­electrochemical studies and DFT calculations confirm that N_2_ binding by the {LNi_2_} platform occurs only in the regime of the mixed-valent Ni^II^Ni^I^ species, while both oxidation and reduction induce the release of N_2_ from **3** ^**N2**^; the latter represents a redox-induced electron transfer (RIET) process where metal reduction leads to N_2_^•–^ oxidation due to intramolecular back electron transfer. These findings offer new perspectives for understanding the multi-e^–/^H^+^ scenarios of N_2_ fixation via hydride intermediates inspired by the FeMoco function, and for the development of synthetic platforms that avoid strongly reducing conditions for N_2_ activation.

## Graphical abstract

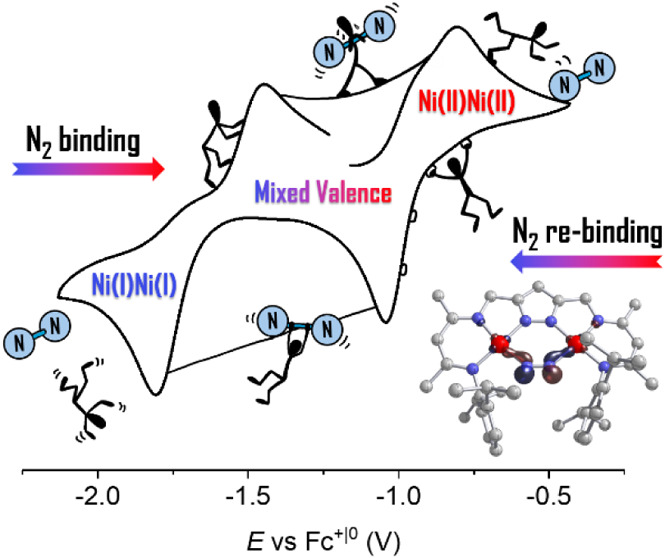

## Introduction

The fixation of atmospheric dinitrogen represents one of the most important transformations for both nature and mankind, but it is highly challenging due to the chemical inertness of the N_2_ molecule.^1,2^ In nature, the reduction of N_2_ is accomplished by the metalloenzyme nitrogenase whose FeMo cofactor uses eight protons and eight electrons together with 16 ATPs to transform N_2_ into two NH_3_ (eq 1, where ATP = adenosine triphosphate, ADP = adenosine diphosphate, P_i_ = inorganic phosphate).^3^

1 $$N_{2}+8H^{+}+8e^{-}+16\mathrm{ATP}\to 2{\mathrm{NH}}_{3}+H_{2}+16\mathrm{ADP}+16P_{i}$$

The use of (at least) two reducing equivalents and four ATPs to produce H_2_ is assumed to be required thermodynamically, to compensate for the energetic penalty of N_2_ binding by exergonic H_2_ release,^4^ and also mechanistically. The Lowe-Thorneley scheme^5^ provides a kinetic model where the first four reducing equivalents are accumulated into two hydrides, likely located at the central belt of the FeMo cofactor, and N_2_ binding to a diazene-like intermediate is then coupled to the reductive elimination of H_2_ (Scheme 1, top; it should be noted that alternative hydride arrangements at the FeMoco belt region have been proposed).^4,6,7^ In currently discussed scenarios, the initial substrate binding is assumed to occur between two iron atoms of the Fe/S cluster core, where both end-on and bridging N_2_ binding modes have been suggested.^7^ It is interesting to note that bent μ_1,2_-bridging substrate binding on the catalyst surface, viz. the α-N_2_ intermediate that precedes N–N bond cleavage, is also considered for the industrial heterogeneous Haber–Bosch process.^8^

**Scheme 1:**
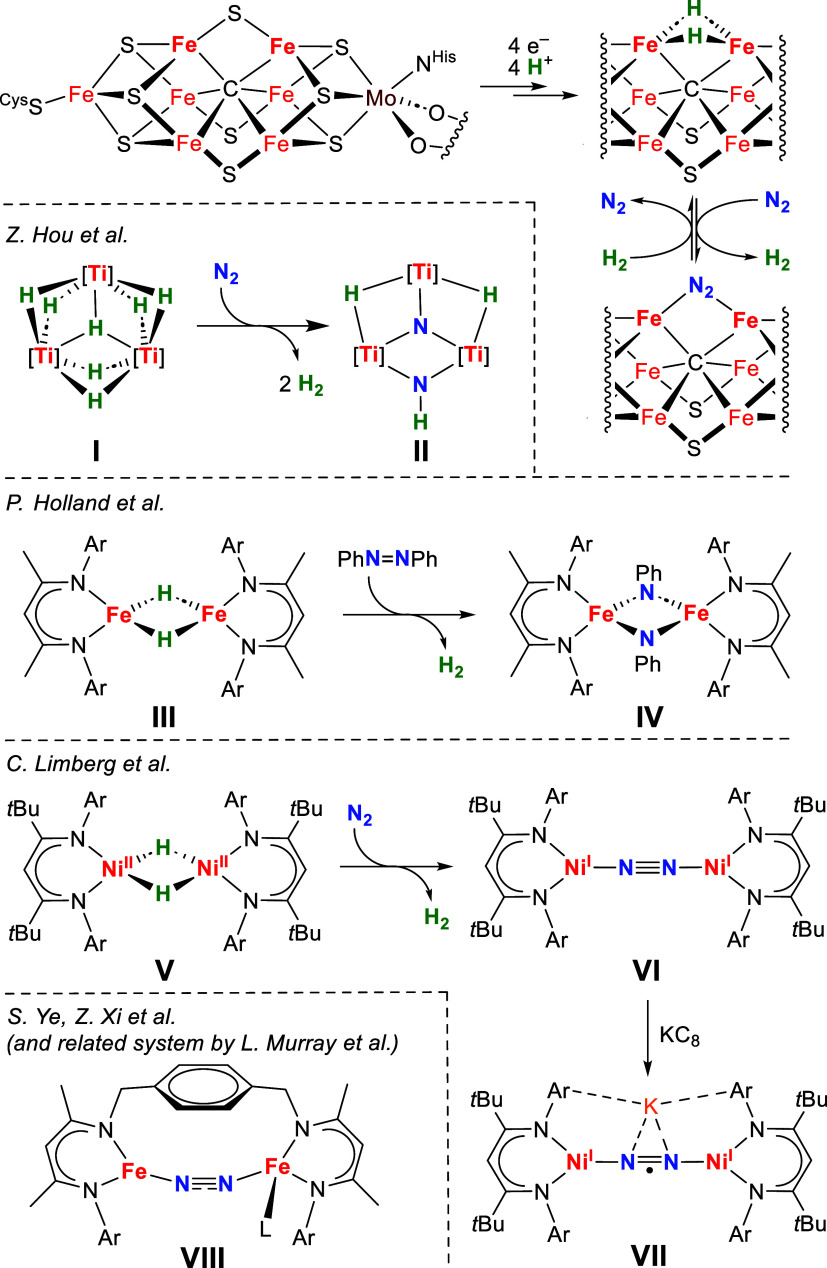
H_2_-Releasing Activation of N_2_ and Related Substrates by Nitrogenase (Top: Only One of Several Proposed Hydride Arrangements) and Selected 3d Metal Complexes

Using hydrides as an electron reservoir for reductive N_2_ fixation, as proposed for FeMoco, circumvents the need for highly reduced and coordinatively unsaturated metal ions. Hence, various molecular transition metal hydride complexes have been investigated in this context, aiming at synchronizing the elimination of H_2_ with the reduction, usually by two e^–^, of the N_2_ substrate.^9–11^ Particularly notable are the early examples of titanium and zirconium hydrides studied by Brintzinger, Bercaw, and Chirik^12^ as well as a series of oligonuclear chromium and titanium polyhydride complexes reported by Hou et al. where the hydrides also serve as a proton source for N–H bond formation.^13^ For example, the titanium polyhydride complex **I** (Scheme 1) upon H_2_ release even cleaves the N≡N bond. The mechanistic scenario involves initial N_2_ binding in end-on/side-on fashion (μ-η^1^:η^2^:η^2^-N_2_) to then give a μ-N/μ_3_-N dinitrido species followed by intramolecular hydrogen migration to the μ-N nitrido unit in **II**.^14^

For late 3d transition metal hydride complexes capable of reductive small substrate activation, β-diketiminates are a privileged ligand class.^15,16^ For example, Holland et al. found that the Fe^II^ hydride dimer **III** (Scheme 1) cleaves azobenzene (PhN=NPh) in a 4e^–^ process. Kinetic and computational studies indicated that azobenzene coordination occurs prior to rate-limiting H_2_ elimination; two of the four reducing equivalents needed to form **IV** are provided by H_2_ reductive elimination and two more by oxidation of the metal ions.^17^ In many cases, however, H_2_ elimination coupled to N_2_ binding goes along with minimal reductive N_2_ activation.^18^ A prominent example is Limberg’s highly reactive Ni^II^ hydride dimer **V** which in contact with N_2_ readily releases H_2_ to give the dinickel­(I) complex **VI** with a linearly bridging μ_1,2_-N_2_ that is only weakly activated, as evidenced by the N–N bond length (1.120(4) Å) and stretching frequency (*ṽ*_NN_ = 2164 cm^–1^) in comparison to the values for free N_2_ (1.098 Å and 2359 cm^–1^, respectively).^19^ **VI** can be reduced by 1e^–^ to **VII** (and even further by another 1e^–^) using strong reductants such as KC_8_; the additional electron in **VII** populates a π* orbital of the N_2_ moiety leading to a unique Ni^I^–(N_2_^•–^)–Ni^I^ core that exhibits much more pronounced substrate activation (1.143(8) Å; *ṽ*_NN_ = 1825 cm^–1^).^20^

In contrast to the bioinspired hydride approach, common N_2_ activation strategies using nonhydride transition metal complexes typically involve strong external reducing agents such as, e.g., KC_8_.^21^ However, these are often incompatible with reaction conditions for subsequent functionalization of the activated N_2_, or may lead to over-reduction. Elucidating the exact redox levels of the transition metal complex core competent for N_2_ binding is thus of key importance. Using Fe^II^ bromido complexes of arene-bridged tris or bis­(β-diketiminato) scaffolds and the strong reductants KC_10_H_8_ or KC_8_, respectively, Murray et al.^22^ as well as Ye and Xi et al.^23^ isolated di-iron complexes where the μ_1,2_-N_2_ substrate is hosted in a constrained, bent bridging mode (∠Fe–Ct_N2_–Fe = 154–159°; Ct_N2_ is the center of the N_2_ unit). In case of **VIII**, the electronic structure was described as a resonance hybrid between high-spin Fe^II^/triplet N_2_^2–^ and high-spin Fe^I^/neutral N_2_ (*ṽ*_NN_ = 1780 cm^–1^), and 1e^–^ reduction was found to populate a nonbonding Fe-centered 3d orbital.^23^ Also, for Murray’s tris­(β-diketiminato) cyclophane based mixed-valent Fe^I^–(μ_1,2_-N_2_)–Fe^II^ system (ṽ_NN_ = 1932 cm^–1^) no significant unpaired spin contribution was found on the N_2_ substrate.^22^

We have developed a dinickel platform based on a compartment pyrazolato/β-diketiminato hybrid ligand L^3–^ where redox equivalents can be stored as hydrides in the dinickel­(II) dihydrido complex [KL­(Ni–H)_2_] (**1** ^**K**^; Scheme 2).^24^ Due to the constraints of the {LNi_2_} scaffold the two hydrides are located in close proximity, and addition of various substrates such as phenylacetylene, O_2_, NO, PhNO and S_8_ to **1** ^**K**^ triggers rapid release of H_2_ and binding of the 2e^–^ reduced substrate within the bimetallic pocket (**X**), reminiscent of the functional principle of nitrogenase.^24–29^ Furthermore, it was previously shown that slow H_2_ release from **1** ^**K**^ in the absence of substrate leads to the highly reactive complex **2** ^**K**^ with two ″T-shaped″ Ni^I^ ions (which can also be prepared independently by KC_8_ reduction of LNi^II^_2_Br) while addition of two equivalents of the strong acid [H­(OEt_2_)_2_]­BAr_4_^F^ leads to peripheral ligand protonation that triggers reductive H_2_ elimination (**IX**; Scheme 2).^30^

**Scheme 2:**
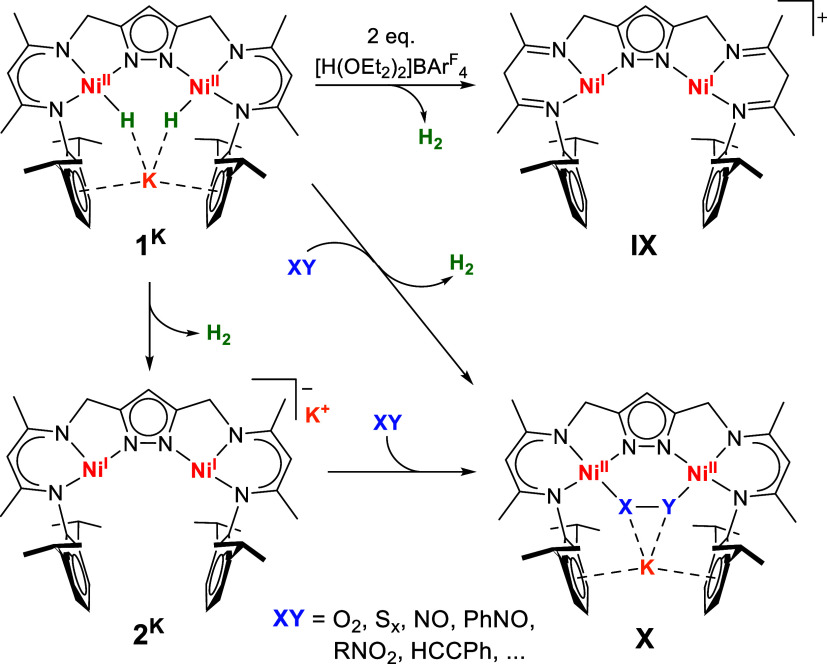
H_2_-Releasing Reductive Small-Molecule (XY) Activation from Dinickel Dihydride Complex **1^K^**

In view of our recent isolation of dicobalt­(I) complexes [LCo^I^_2_(N_2_)]^−^ that enforce acute Co-Ct_N2_-Co angles of the N_2_ substrate hosted within the preorganized bimetallic cleft,^31^ we surmised that the dinickel­(II) dihydrido complex **1** ^**K**^ might be competent of reductively activating N_2_ in a constrained binding mode, with the hydrides serving as an electron reservoir akin to the FeMoco mechanism. However, neither dihydrido complex **1** ^**K**^ nor dinickel­(I) complexes **2** ^**K**^ and **IX** react with N_2_. Here we report that reductive N_2_ binding at the {LNi_2_} scaffold can be achieved by triggering H_2_ release from **1** ^**K**^ via 1e^–^ oxidation, including by H^+^ addition. We evidence an unusual highly bent coordination of an N_2_^•–^ radical in the bimetallic pocket, and we demonstrate that only the mixed-valent Ni^II^Ni^I^ form of the {LNi_2_} platform is competent for reductive N_2_ binding, while both oxidation and further reduction lead to N_2_ release.

## Results and Discussion

Treatment of a THF solution of **1** ^**K**^ with one equivalent of a mild 2,6-lutidinium acid, [HLut]­OTf or [HLut]­BAr_4_, under N_2_ at room temperature (Scheme 3) results in immediate gas evolution and a color change from orange to brown-red. The gas was identified as H_2_ by gas chromatography (GC) analysis of the headspace of the reaction mixture and was quantified as >1 equiv H_2_ (Table S6).^32^ This suggested that one of the two reduction equivalents of the dihydride **1** ^**K**^ was sacrificed by proton reduction and that 1e^–^ reduction of the substrate N_2_ occurred, in contrast to the 2e^–^ reduction scenario of other substrates XY shown in Scheme 2. Single crystals of product complex **3** ^**N2**^ suitable for X-ray diffraction were obtained by layering the reaction mixture with hexanes at −30 °C.

**Scheme 3:**
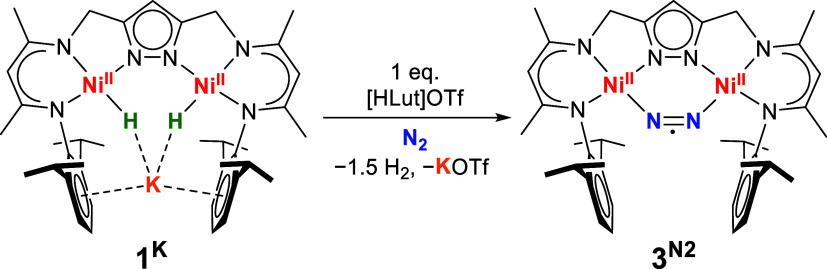
Idealized Reaction Balance for the Proton-Triggered H_2_-Releasing N_2_-Binding by the Dihydride Complex 1^K^ to Give **3^N2^**

**3** ^**N2**^ features a μ-η^1^:η^1^-N_2_ unit in the clamp of the pyrazolate-based {LNi_2_} scaffold (Figure 1). The two nickel ions are found in square planar geometry, as was observed for all known Ni^II^ complexes of the ligand L^3–^, and the central six-membered {Ni_2_N_4_} ring in **3** ^**N2**^ is almost planar (the dihedral angle ∠Ni1–N7–N8–Ni2 is 2.82°). The bound N_2_ is slightly elongated (*d*(N7–N8) = 1.132(2) Å) compared to free N_2_ (1.098 Å),^19^ indicating some activation of the substrate. Interestingly, the rather rigid preorganized {LNi_2_} scaffold with *d*(Ni1···Ni2) = 3.962(2) Å enforces a highly bent N_2_ binding mode with ∠Ni–Ct_N2_–Ni = 115.8(1)°. This bent situation is much more pronounced than in recently reported complexes with constrained geometry and N_2_ bridging that deviates from linearity (with ∠Fe–Ct_N2_–Fe ∼155°),^22,23,33–35^ and even more acute than in our recently reported [LCo^I^_2_(N_2_)]^−^ complex (123.5°).^31^

**Figure 1:**
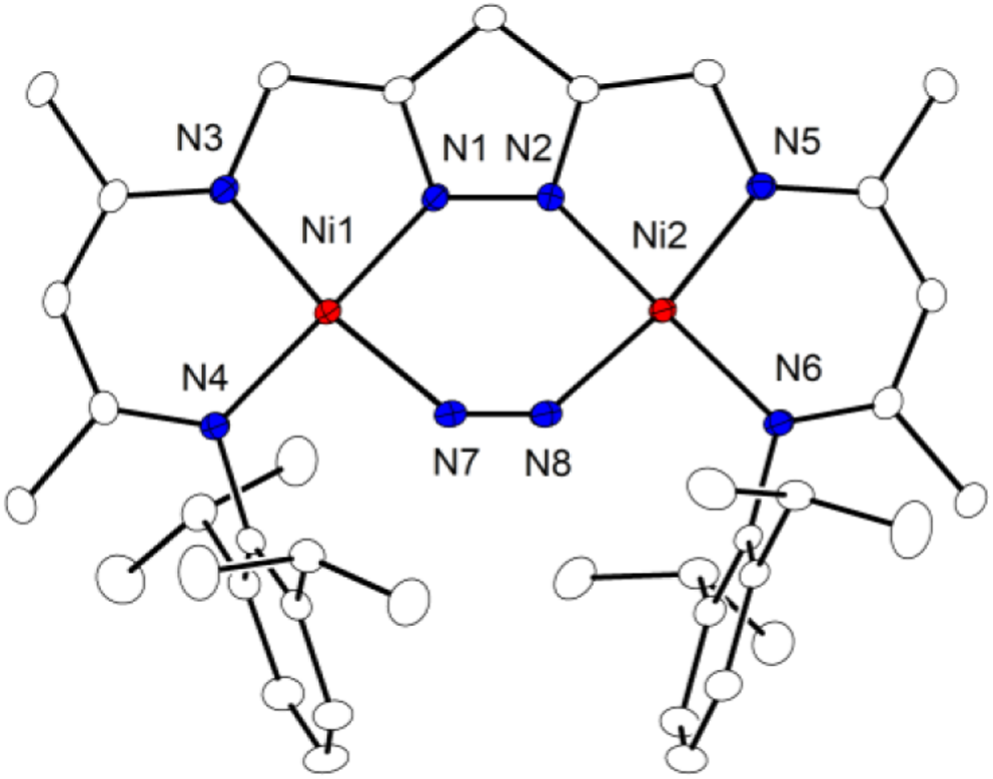
Molecular structure of **3** ^**N2**^. Thermal ellipsoids are drawn at the 30% probability level. Hydrogen atoms are omitted for clarity. Selected bond lengths (Å) and angles [°]: Ni1···Ni2 3.9621(5), Ni1–N1 1.8650(12), Ni1–N3 1.8859(11), Ni1–N4 1.8978(12), Ni2–N2 1.8737(12), Ni2–N2 1.8737(12), Ni1–N7 1.8844(13), Ni2–N8 1.8828(13), N7–N8 1.1324(18); N1–Ni1–N7 87.07(5), N2–Ni2–N8 87.37(5), N8–N7–Ni1 139.43(11), N7–N8–Ni2 137.93(11).

IR and Raman spectra of crystalline **3** ^**N2**^ show a sharp band at 1894 cm^–1^ and 1898 cm^–1^, respectively, assigned to the N7–N8 stretching vibration as corroborated via ^15^N_2_ labeling (Figure 2); this indicates significant activation of the bound N_2_ substrate (cf. ṽ_NN_ = 2359 cm^–1^ for free N_2_).^19^ The N7–N8 bond length and the *ṽ*_NN_ band lie in between those for the neutral and singly reduced linear Ni^I^-(μ-η^1^:η^1^-N_2_)-Ni^I^ systems **VI** and **VII** reported by Limberg et al. (1.120(4) Å/2164 cm^–1^ and 1.143(8) Å/1825 cm^–1^, respectively).^20^ Except for the latter and its doubly reduced congener^20^ as well as the formal dinickel(0) complex (^*i*^Pr_3_P)_2_Ni^0^-NN-Ni^0^(P^*i*^Pr_3_)_2_ (*ṽ*_NN_ = 1908 cm^–1^),^36^ complexes with a Ni-(μ-η^1^:η^1^-N_2_)-Ni core usually show much higher *ṽ*_NN_ > 2000 cm^–1^ for both, Ni^0^ and formal Ni^II^ systems,^36–40^ and the same is true for most mononuclear Ni^0^, Ni^I^ or formal Ni^II^ complexes with end-on bound terminal N_2_.^39,41–48^ Recently, β-diketiminato-based complexes with Ni–NN–Mg^II^(thf)_4_ and Ni–NN–Ca^II^(thf)_4_ cores (*ṽ*_NN_ = 1923, 1921 cm^–1^) have been reported whose spectroscopic signatures suggested a Ni^I^ oxidation state.^49^ Apart from **VII** and its doubly reduced congener,^20^ the present **3** ^**N2**^ with its highly bent N_2_ binding shows the lowest *ṽ*_NN_ and hence the most pronounced reductive N_2_ activation reported so far for nickel dinitrogen complexes. Still, the bound substrate in **3** ^**N2**^ undergoes exchange with free N_2_, as evidenced by exposing [LNi_2_(μ_1,2_-^15^N_2_)] to ^14^N_2_.

**Figure 2:**
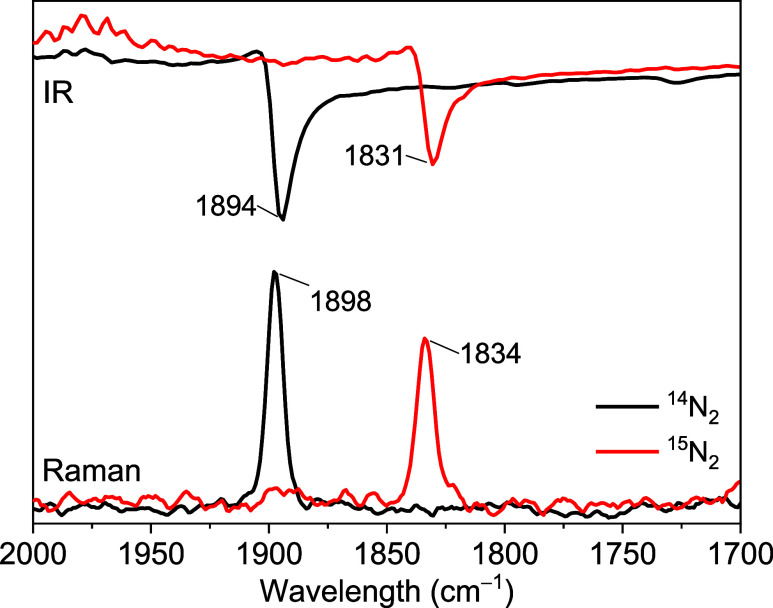
IR and Raman spectra of crystalline **3** ^**N2**^ and its ^15^N_2_ isotopologue recorded at room temperature.

Charge considerations (*viz*. the absence of a counterion) indicated that **3** ^**N2**^ has an uneven electron count. The paramagnetic nature of **3** ^**N2**^ is in accordance with its ^1^H NMR spectrum that shows broad resonances in the range from 10 to −7 ppm (in THF-*d*_8_ at room temperature; Figures S19 and S20). The EPR spectrum of **3** ^**N2**^ recorded in frozen toluene at 129 K (Figure 3) shows a rhombic signal indicative of an *S* = 1/2 system, which could be well simulated with *g* = [1.994, 2.070, 2.149] (*g*_av_ = 2.07) and hyperfine coupling with two ^14^N atoms *A*(^14^N) = [36, 58, 33] MHz (*A*_iso_(^14^N) = 42 MHz), indicating a significant contribution of the N_2_ unit to the singly occupied molecular orbital (SOMO). We attribute this to the pronounced preference of Ni^II^ (low-spin d^8^) character in dinickel complexes LNi_2_(μ-XY) hosting any substrate XY within the bimetallic pocket, because the ligand scaffold L^3–^ with its two pincer-type tridentate compartments enforces an overall square planar metal ion coordination environment.^24–29^

**Figure 3:**
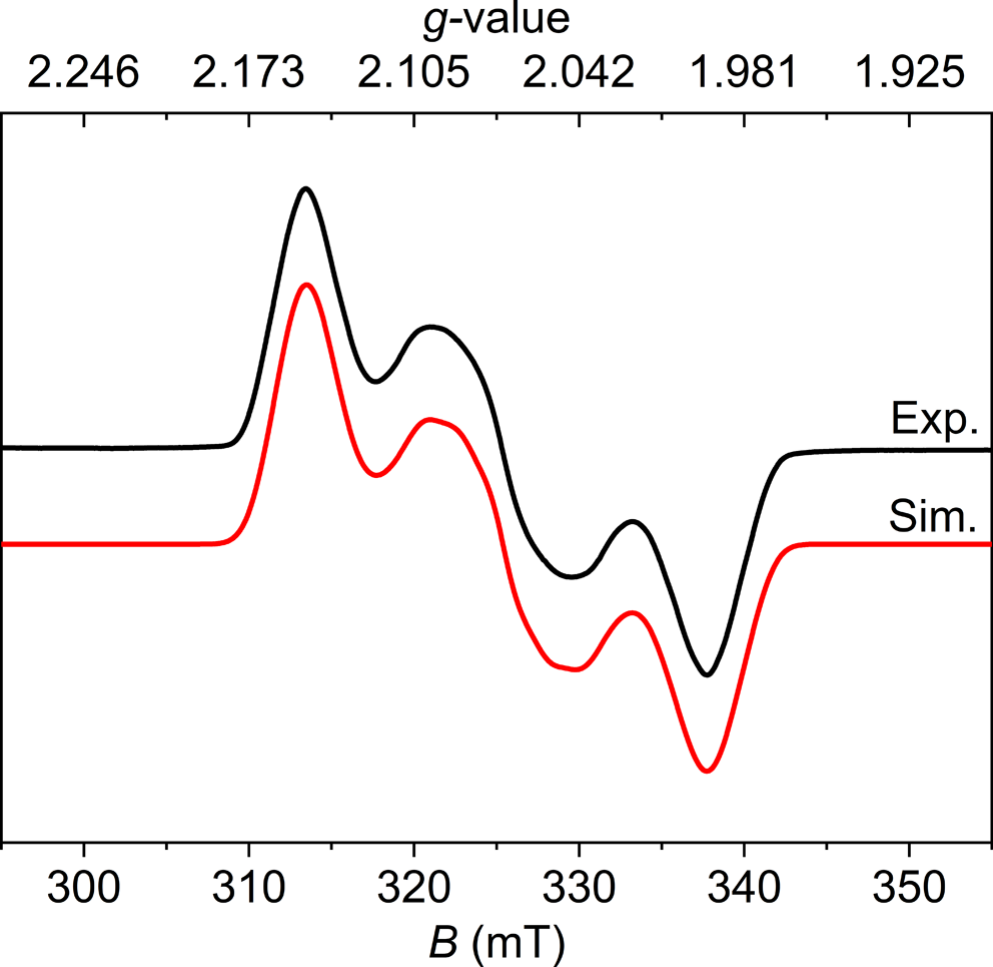
X-band EPR spectrum (9.429 GHz, microwave power 3.93 mW) of **3** ^**N2**^ in toluene (2.5 mM) at 129 K. Experimental data is displayed as black line, while the simulated spectrum is reported in red with *g* = [1.994, 2.070, 2.149], *A*(^14^N_1_) = [36, 58, 33] MHz.

Density functional theory (DFT) calculations predict complex **3** ^**N2**^ as a C_2_-symmetric species (Figure S6). The geometry optimization converges at an N–N bond length in the N_2_-derived ligand of 1.143 Å with Ni–N and Ni···Ni distances of 1.874 and 3.968 Å, respectively, all in consonance with the corresponding bond lengths extracted from X-ray diffraction data (1.132(2), 1.883(2) and 3.962(2) Å, respectively). The computed frequency for the N–N stretching mode of 1892.6 cm^–1^, obtained after scaling with the associated factor of 0.959 determined for the r^2^SCAN-3c method according to the recipe of Truhlar and co-workers,^50,51^ is in excellent agreement with the experimentally assigned IR signature (1894 cm^–1^). As inferred from the EPR data, **3** ^**N2**^ has a doublet-spin ground state (*S* = 1/2), and the unpaired electron is found to be evenly delocalized over the {NiNNNi} bridge (natural spin population Ni_2_: 44%, N_2_: 49%; Figure 4a). Assuming an oxidation state of +II for both nickel atoms within the square planar geometry, the *d*_*x* ^2^__–*y* ^2^_ orbitals will be highest in energy. The SOMO in Figure 4b shows an interaction of these nickel *d*-orbitals with an in-plane π_∥_^*^ orbital of the N_2_ moiety. This appears to confirm an N_2_^•–^ radical anion fragment, in which the additional electron resides in an antibonding π* orbital. The alternative description of nickel with an overall formal oxidation state of +I would infer back-bonding from both metal *d*_*x* ^2^–*y* ^2^_-orbitals into an empty π* orbital of a neutral N_2_ moiety. The unpaired electron, however, is not strongly or even exclusively located at the metal centers, in accordance with the EPR spectroscopic evidence. The computed *g*-tensor components of *g*_iso_ = 2.057 with *g*_*x*,*y*,*z*_ = [1.993, 2.062, 2.117] are in good agreement with the low-temperature EPR spectrum, and also the computed HFC parameters with anisotropy resulting from the spin-dipole part of the *A* tensor, i.e., *A*_*x*,*y*,*z*_(^14^N) = [30.2, 70.5, 33.2] MHz, are in reasonable agreement with the results from simulation of the experimental spectrum. This corroborates that the computational model represents the electronic structure of **3** ^**N2**^.

**Figure 4:**
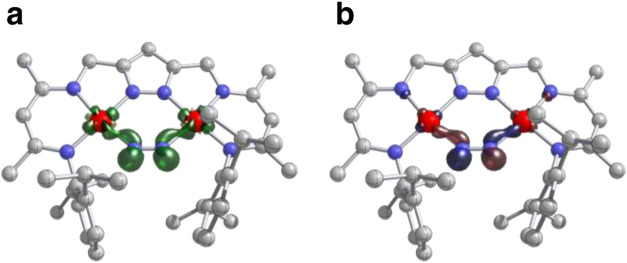
Isocontour surface plots computed at the PBE0/ZORA-def2-QZVPP//r^2^SCAN-3c level for the doublet (*S* = 1/2) ground state spin electromer of complex **3** ^**N2**^; (a) spin density at 0.01 *a*_0_^–3^; natural spin populations from NBO analysis: Ni: 0.220; N: 0.246. (b) Singly occupied molecular orbital (α SOMO) at ± 0.1 *a*_0_^–3/2^.

According to a natural bond orbital (NBO) analysis, the two Ni centers have four doubly occupied lone-pair orbitals each, supporting the notion of Ni^II^ d^8^ configurations (Figure S7). The {NiNNNi} bridge is only mildly polarized with a charge distribution of *q*(Ni): + 0.90 and *q*(N): −0.15. The Wiberg bond index (WBI) in the natural atomic orbital (NAO) basis indicates a moderately activated N_2_ moiety with a bond order between a double and a triple bond, WBI­(N,N): 2.49, and an accordingly low Ni–N bond order of roughly 1/2, WBI­(Ni,N): 0.41. In the localized NBO picture, the symmetry of the complex forces the delocalized single electron to distribute into two α spin and one β spin natural localized molecular orbital (NLMO). The metal–nitrogen bridge therefore features two singly occupied Ni–N σ-bond NLMOs, which are clearly polarized toward nitrogen, and which result from a *d*_*x* ^2^–*y* ^2^_(Ni) orbital interacting with the in-plane π_∥_ orbital of N. The N_2_ ligand features two doubly occupied lone-pair NLMOs, a σ- and an out-of-plane π_⊥_-bond, and a singly occupied π_∥_ NLMO (Figure 5). The collective spectroscopic and computational evidence thus supports a predominant N_2_^•–^ radical character of the bound substrate.

**Figure 5:**
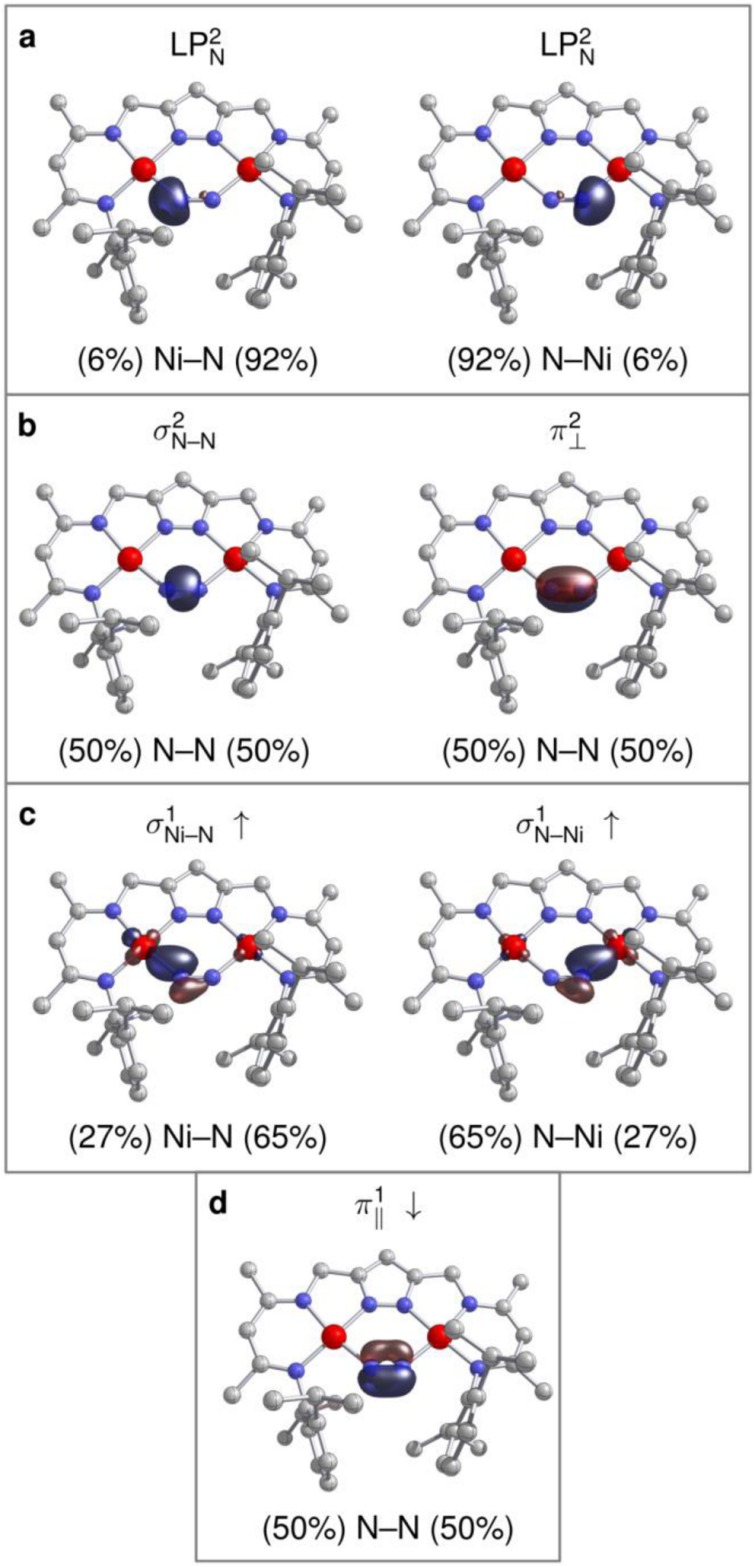
Selected NLMOs from NBO analysis of **3** ^**N2**^ at the PBE0/ZORA-def2-QZVPP//r^2^SCAN-3c level. Isosurfaces at ±0.1 *a*_0_^–3/2^; for doubly occupied NLMOs, the average of α and β spins is shown. (a) Doubly occupied N-centered lone pairs; (b) doubly occupied N–N σ, π orbitals; (c) singly occupied Ni–N σ orbitals (α spin); (d) singly occupied N–N π orbital (β spin).

Since neither the dinickel­(II) dihydride complex **1** ^**K**^, which serves as a masked dinickel­(I) complex, nor the isolated dinickel­(I) complex **2** ^**K**^ reacted with N_2_, we assumed a mixed-valent Ni^II^Ni^I^ species to be responsible for N_2_ binding en route to **3** ^**N2**^. To verify this hypothesis, the oxidative chemistry of complex **2** ^**K**^ was investigated by cyclic voltammetry (CV); these experiments were challenging because **2** ^**K**^ is highly sensitive. The CV of **2** ^**K**^ in THF under an Ar atmosphere exhibits a quasireversible first oxidation at *E*_1/2_ = −1.52 V and a second, reversible oxidation wave at *E*_1/2_ = −1.05 V (*v* = 0.1 V s^–1^, all potentials are referenced internally against the Fc^+|0^ couple, Figure 6, top). The oxidation processes are assigned to consecutive metal-centered Ni^I^Ni^I^→Ni^II^Ni^I^ and Ni^II^Ni^I^→Ni^II^Ni^II^ oxidations, likely accompanied by THF binding to furnish the common 4-fold coordination of the Ni^II^ ions (hence the resulting Ni^II^Ni^I^ and Ni^II^Ni^II^ species are denoted **3** ^**THF**^ and **4** ^**THF**^, respectively). Upon changing the atmosphere to N_2_, the CV shows distinct differences (Figure 6, middle). The first oxidation is slightly shifted to more negative potentials (*E*_pa,1,Ar_ = −1.44 V, *E*_pa,1,N2_ = −1.46 V), the reverse feature vanishes and a new feature appears at *E*_pc,2_ = −1.80 V, indicating a fast chemical follow-up reaction of the mixed-valent Ni^II^Ni^I^ intermediate with N_2_.^52^ Indeed, IR-spectroelectrochemical (IR-SEC) measurements of **2** ^**K**^ under N_2_ atmosphere display the rise of a prominent band at 1898 cm^–1^ upon initial oxidation, which is in excellent agreement with *ṽ*_NN_ found for solid **3** ^**N2**^ (vide infra, Figure S28). This indicates the formation of **3** ^**N2**^ upon 1e^–^ oxidation of **2** ^**K**^ under N_2_. At *E*_pa,2,N2_ = −0.81 V, a further oxidation process appears in the CV data of **2** ^**K**^ under N_2_, which supports formation of **3** ^**N2**^ upon oxidation of **2** ^**K**^, as this process was assigned to the oxidation of **3** ^**N2**^ by investigation of an independently synthesized sample of **3** ^**N2**^ (vide infra).^53^

**Figure 6:**
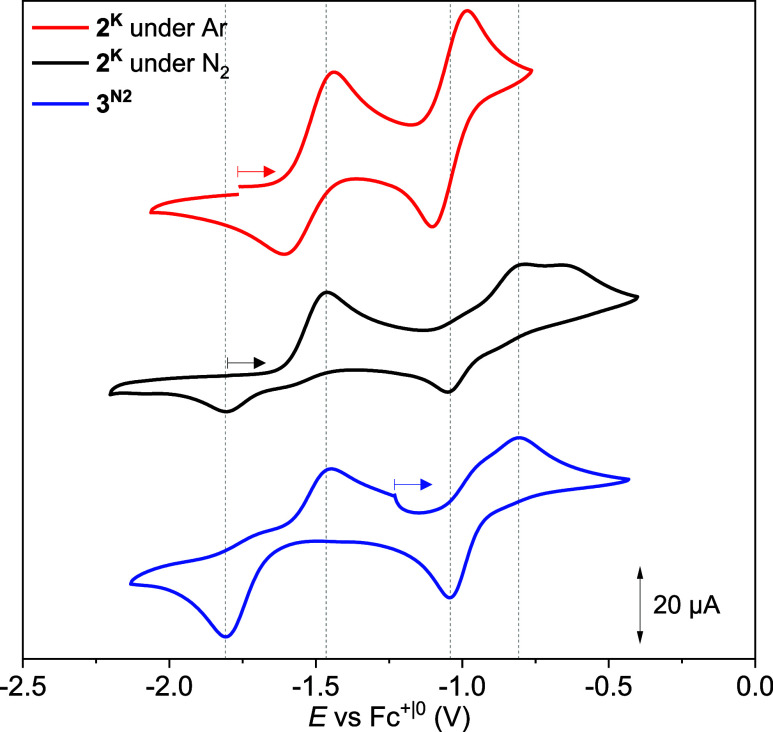
CVs of **2** ^**K**^ under Ar (red trace) and under N_2_ (black trace) and of **3** ^**N2**^ under N_2_ (blue trace), *v* = 0.1 V s^–1^, *c* = 3 mM, 0.2 M ^*n*^Bu_4_NPF_6_ in THF.

Having determined the first oxidation potential of **2** ^**K**^, we investigated the oxidative formation of **3** ^**N2**^ starting with **2** ^**K**^ and using a chemical oxidant. Addition of 1 equiv of ferrocenium hexafluorophosphate (FcPF_6_) to a solution of **2** ^**K**^ under N_2_ atmosphere indeed gives **3** ^**N2**^, as confirmed by ^1^H NMR spectroscopy (Figure S19). Similarly, the reaction of the dihydrido complex **1** ^**K**^ with 1 equiv of FcPF_6_ leads to the release of 1 equiv of H_2_ (identified and quantified by GC headspace analysis) and clean formation of **3** ^**N2**^ according to ^1^H NMR spectroscopy (Table S5 and Figure S20). This synthetic route from **1** ^**K**^ to **3** ^**N2**^ via oxidation with FcPF_6_ (Scheme 4) is more convenient and provides cleaner and more quantitative product formation compared to the protonation of **1** ^**K**^ (Scheme 3); however, the product contains ferrocene as side product which is difficult to separate. During crystallization, due to the highly moisture-sensitive nature of **3** ^**N2**^, partial decomposition giving trace amounts of LNi_2_(μ–OH) can hardly be avoided. For these reasons, the characterizations and reactivity studies were carried out with **3** ^**N2**^ generated *in situ* by adding a solution of **1** ^**K**^ to FcPF_6_ under a N_2_ atmosphere and vigorous stirring. For electrochemical studies, the presence of Fc provides the convenient benefit of an internal standard.

**Scheme 4:**
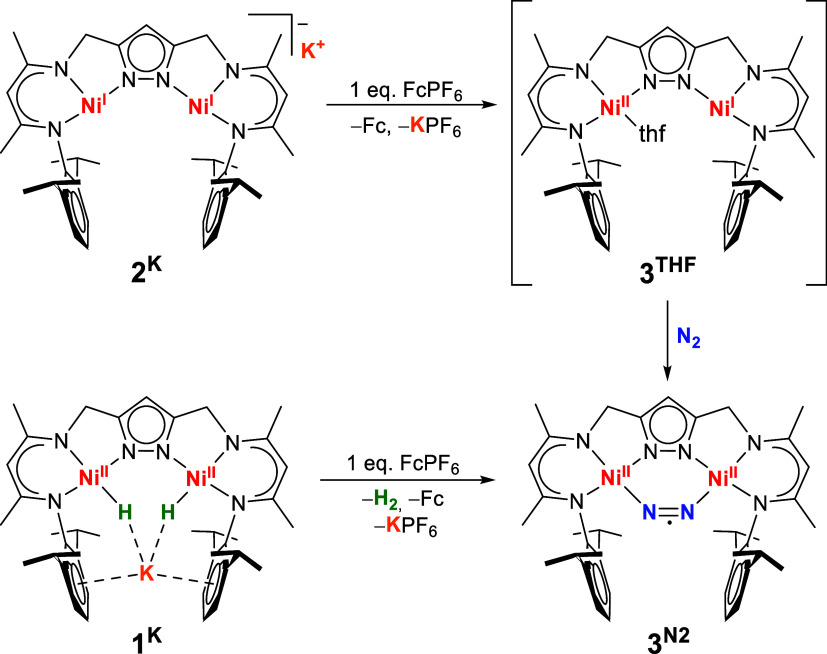
Overview on the One-Electron Oxidation Chemistry of **2^K^** and **1^K^**, Giving the Radical Complex **3^N2^**

Subsequently, the electrochemical properties of *in situ* prepared **3** ^**N2**^ and **3** ^**THF**^ were investigated by CV. The CV data of **3** ^**THF**^ display the same redox events as observed in the CV data of **2** ^**K**^ under Ar (Figure S28, **3** ^**THF**^: *E*_1/2_ = −1.05 V, *E*_pa,1_ = −0.98 V and *E*_pc,2_ = −1.61 V, *E*_1/2_ = −1.52 V). **3** ^**N2**^ exhibits an irreversible oxidation process at *E*_pa,1_ = −0.81 V, which is shifted by 170 mV to a more positive potential with respect to the oxidation of **3** ^**THF**^ (Figure 6, bottom). The potential shift can likely be attributed to the π-accepting character of the μ_1,2_-N_2_ ligand and increased valence delocalization in **3** ^**N2**^ compared to **3** ^**THF**^. Further, **3** ^**N2**^ shows an irreversible reduction process at *E*_p,c_ = −1.80 V (*v* = 0.1 V s^–1^, Figure 6, bottom), which is shifted by −190 mV to more negative potentials compared to mixed-valent Ni^II^Ni^I^ species **3** ^**THF**^. The irreversibility of both oxidative and reductive redox events of **3** ^**N2**^ points to fast follow-up chemical reactions, and thus was investigated in more detail via IR-SEC experiments. The *ṽ*_NN_ stretching of **3** ^**N2**^ at 1898 cm^–1^ readily decreases upon both reduction and oxidation, but it recovers when the formally mixed-valent Ni^II^Ni^I^ species is reformed by oxidation and reduction, respectively (Figure 7). This finding, together with the CV data of **3** ^**N2**^ strongly resembling the CV data of **2** ^**K**^ under a N_2_ atmosphere, indicates that N_2_ binding by the {LNi_2_} platform to form **3** ^**N2**^ only occurs in the regime of the mixed-valent Ni^II^Ni^I^ species. Thus, N_2_ is released from **3** ^**N2**^ both upon 1e^–^ oxidation to give a Ni^II^Ni^II^ species (**4** ^**THF**^, see below) and, somewhat surprisingly, upon 1e^–^ reduction giving the Ni^I^Ni^I^ species **2** ^**–**^ (Scheme 5).

**Figure 7:**
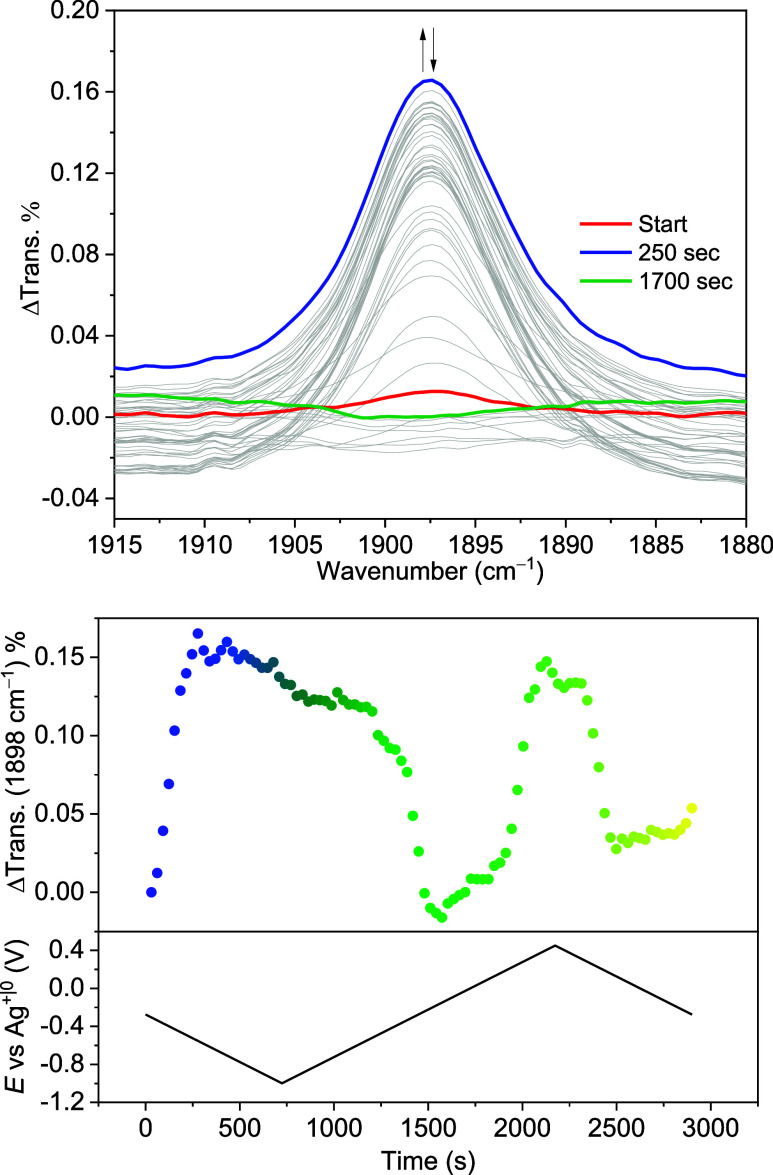
Top: IR spectra of reductive scan of the IR-SEC experiment using a 5 mM solution of **3** ^**N2**^ in THF. Bottom: difference in transmittance at 1898 cm^–1^ vs time for the IR-SEC experiment and applied potential vs time of the experiment, starting from the OCP.

**Scheme 5:**
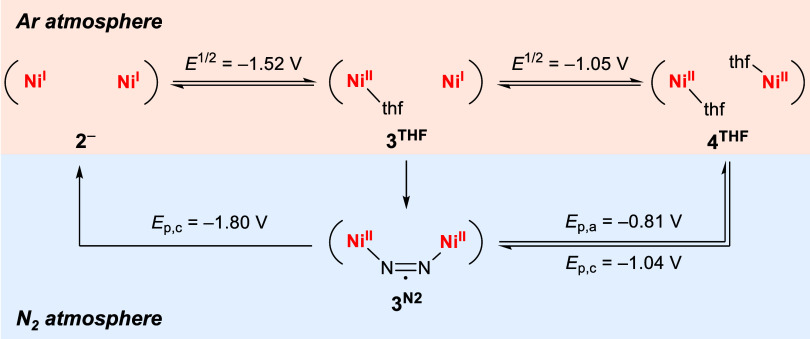
Simplified Overview of Electrochemical Redox Interconversions of the Species Reported in This Work; the Ligand Scaffold Is Omitted for Clarity

DFT calculations confirm that among the dinickel platforms with Ni^I^ and Ni^II^ cores [LNi_2_^I^]^−^ (**2** ^**–**^), [LNi^I^Ni^II^] (**3**) and [LNi_2_^II^]^+^ (**4** ^**+**^), only the mixed-valent Ni^I^Ni^II^ species **3** binds dinitrogen exergonically with a binding energy Δ_r_ *G*(**3** ^**N2**^) of −3 kcal mol^–1^ with respect to the corresponding isolated fragments N_2_ and **3** in their ground states (Table S4). NBO analysis of the NLMOs in the dinickel­(I) anion [LNi_2_^I^(N_2_)]^−^ (**2** ^**N2–**^) reveals no Ni–N_2_ bonding interaction, but rather a full triple bond in the N_2_ unit and two singly occupied *d*_*x* ^2^__–*y* ^2^_ lone-pair NLMOs with negligible contributions from the N_2_ fragment (Figure S8). The cationic dinickel­(II) congener [LNi_2_^II^(N_2_)]^+^ (**4** ^**N2+**^) features a closed-shell dinickel­(II) fragment with unoccupied *d*_*x* ^2^–*y* ^2^_ orbitals and a triply bonded N_2_ moiety. Concomitantly, the WBI­(Ni,N) drops from 0.41 in **3** ^**N2**^ to 0.33 in **2** ^**N2–**^ and 0.32 in **4** ^**N2+**^ while WBI­(N,N) increases from 2.49 to 2.60 and 2.72 in the same series, respectively. This is mirrored in the Ni–N and N–N distances (Figure S9).

Attempts were then made to identify and trap the chemically generated species **3** ^**THF**^ and **4** ^**THF**^. NMR analysis of a solution of **3** ^**N2**^ after chemical reduction with 1 equiv of CoCp*_2_ confirmed the formation of anionic **2** ^**–**^, while a new paramagnetic species is obtained upon addition of 1 equiv of FcPF_6_ to **3** ^**N2**^ (Figures S21 and S22). The same paramagnetic species was obtained from the reaction of **1** ^**K**^ with 2 equiv of FcPF_6_ under either N_2_ or Ar atmosphere in THF. We therefore assume that this new oxidized species is [LNi^II^_2_(THF)_2_]^+^ (**4** ^**THF**^) containing two high-spin (*S* = 1) Ni^II^ ions whose fourth coordination sites are filled by THF solvent molecules (Scheme 5). **4** ^**THF**^ is sensitive and rather unstable, and it could not be isolated. However, addition of 2 equiv of PMe_3_ to a solution of **4** ^**THF**^ yielded the corresponding complex [LNi^II^_2_(PMe_3_)_2_]^+^ (**4** ^**PMe3**^) that crystallized as the PF_6_^–^ salt upon diffusing pentane into the THF solution at −35 °C. The molecular structure of **4** ^**PMe3**^ is shown in Figure 8 and indirectly corroborates the identity of **4** ^**THF**^. The Ni^II^ ions in **4** ^**PMe3**^ have a slightly distorted square planar geometry (τ_4_ = 0.3 for both Ni^II^)^54^ and a wide Ni···Ni separation of 4.369(2) Å because of the steric demand of the two PMe_3_ units. SQUID magnetometry of a solid sample of **4** ^**PMe3**^ shows it to be diamagnetic (*S* = 0 ground state; see Figure S14), in accordance with low-spin d^8^ Ni^II^ ions.

**Figure 8:**
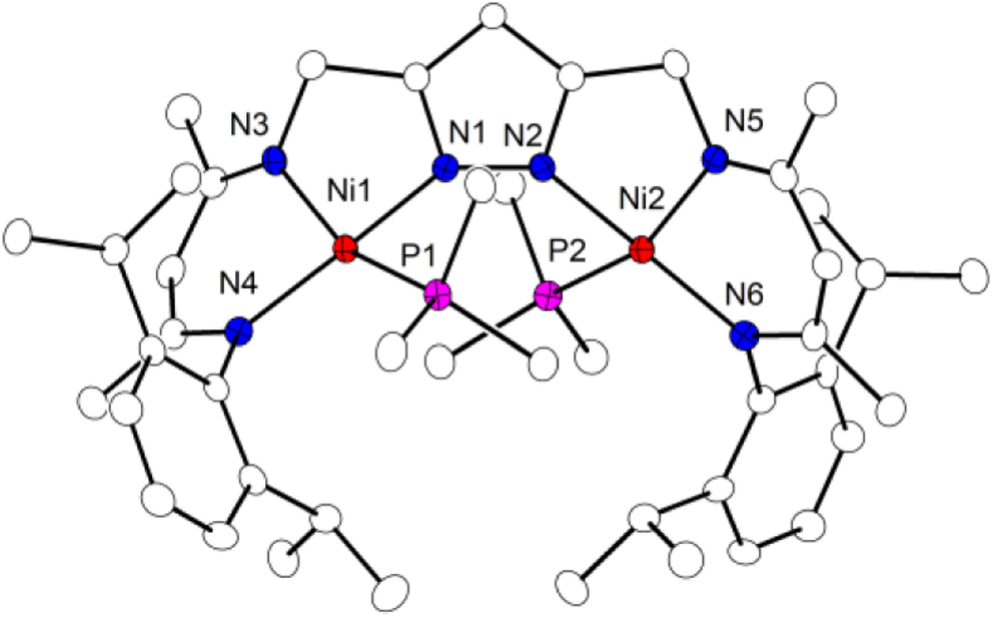
Molecular structure of **4** ^**PMe3**^ (30% thermal ellipsoids). The PF_6_^–^ counterion and hydrogen atoms are omitted for clarity. Selected bond lengths [Å] and angles [°]: Ni1···Ni2 4.3685(12), Ni1–N1 1.929(5), Ni1–N3 1.885(5), Ni1–N4 1.912(5), Ni1–P1 2.2707(17), Ni2–N2 1.932(5), Ni2–N5 1.883(5), Ni2–N6 1.897(5), Ni2–P2 2.2624(17); N4–Ni1–N1 163.5(2), N3–Ni1–P1 154.41(16), N4–Ni1–P1 103.52(15), N1–Ni1–P1 90.41(15), N6–Ni2–N2 163.0(2), N5–Ni2–P2 152.57(15), N6–Ni2–P2 103.49(15), N2–Ni2–P2 89.80(15).

The fleeting mixed-valent Ni^II^Ni^I^ intermediate **3** ^**THF**^ can be accessed by oxidation of **1** ^**K**^ with 1 equiv of FcPF_6_ under argon atmosphere, triggering H_2_ release (Scheme 6). The EPR spectrum of the reaction mixture displays a rhombic signal with *g* = [2.045, 2.143, 2.328] in accordance with a Ni^II^Ni^I^ species with *S* = 1/2 ground state (Figure S24). The ^1^H NMR spectrum in THF-*d*_8_ shows broad resonances in the range of +14 to −16 ppm. The paramagnetic species remains stable in solution under Ar at room temperature for about 1 day, and if exposed to N_2_ atmosphere, it forms **3** ^**N2**^ (Figure S23). The mixed valence complex could be trapped by the addition of 1 equiv of 2,6-lutidinium tetrafluoroborate ([HLut]­BF_4_), giving the new Ni^II^Ni^I^ complex **5** that was crystallized by layering the THF reaction mixture with hexanes at −35 °C.

**Scheme 6:**
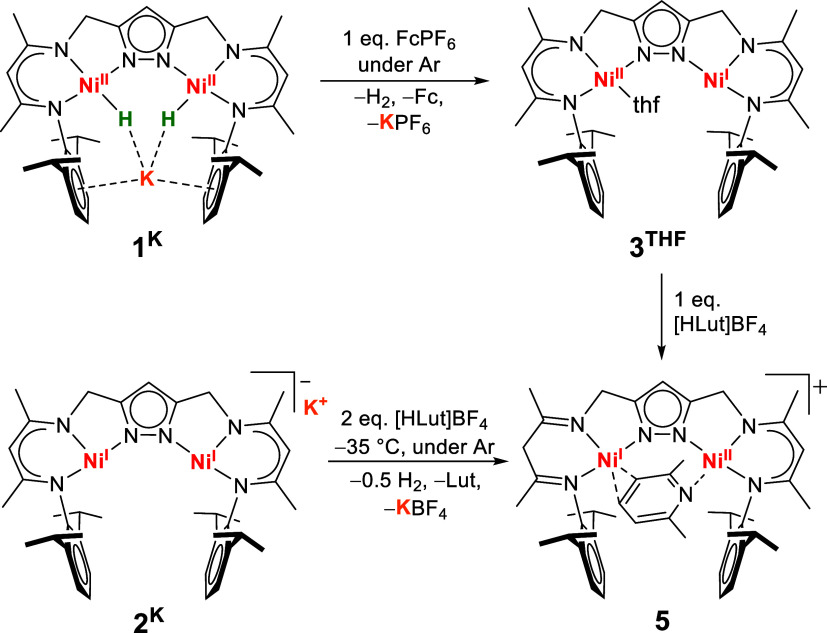
Synthesis of Mixed-Valent **3^THF^** and **5**

The molecular structure of **5** (Figure 9) shows C protonation of one of the β-diketiminato arms and the incorporation of lutidine in the bimetallic cavity. 2-fold γ-C protonation of the L^3–^ ligand platform was previously observed upon treatment of **1** ^**K**^ with 2 equiv of the strong acid [H­(OEt_2_)_2_]­BAr_4_^F^ (accompanied by the formation of H_2_).^30^ Protonation in the γ-position of one β-diketiminato arm to give a β-diimine subunit in **5** is evidenced by the short C=N bonds involving N3 and N4 (1.279(4) and 1.284(3) Å) compared to the longer C=N bonds involving N5 and N6 in the unperturbed β-diketiminato arm (1.320(4) and 1.339(4) Å). The IR spectrum of solid **5** (Figure S16) shows bands at 1654 and 1627 cm^–1^ assigned to *ṽ*_CN_ stretching of the β-diimine unit and likely also lutidine (**IX** shows *ṽ*_CN_ at 1670 cm^–1^). Lutidine is found N-bound to Ni2, which adopts a roughly square planar coordination geometry and is thus identified as the Ni^II^ (d^8^; *S* = 0) ion, while Ni1 shows weak interaction with a C=C edge of the lutidine and is assigned as a Ni^I^ (d^9^; *S* = 1/2) ion. Complex **5** is also accessible directly by reacting the Ni^I^Ni^I^ complex **2** ^**K**^ with 2 equiv of [HLut]­BF_4_ under Ar atmosphere (Scheme 6); in this case, one equivalent of acid serves as an oxidant liberating H_2_ (detected in the headspace via GC; Figure S26), and one equivalent traps generated **3** ^**THF**^ to form **5**.

**Figure 9:**
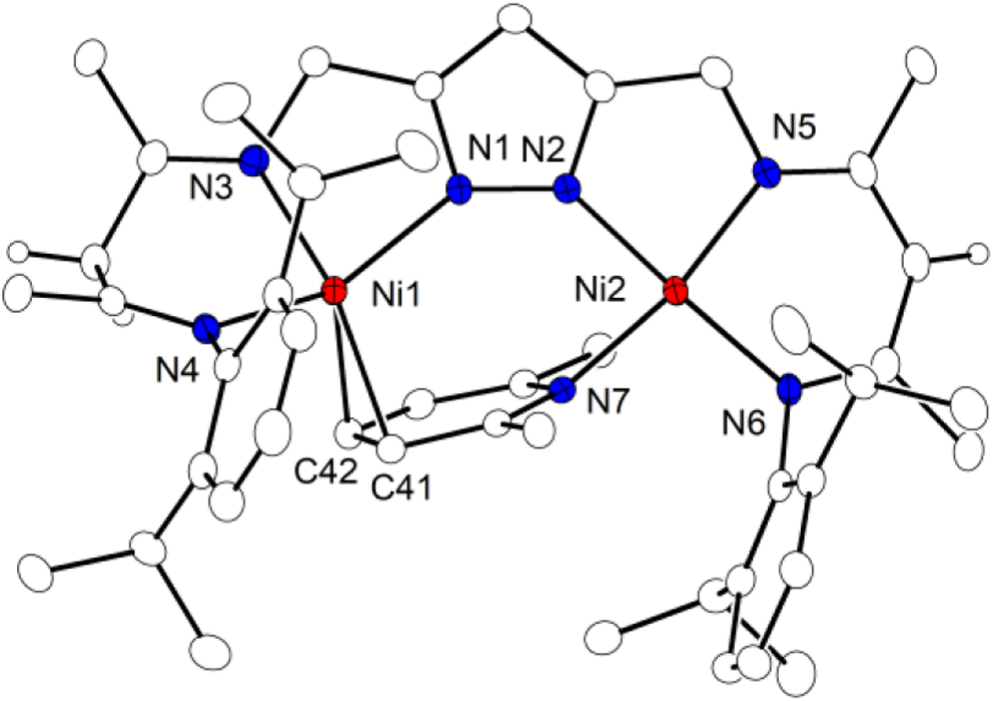
Molecular structure of **5** (30% thermal ellipsoids). The BF_4_^–^ counterion and most hydrogen atoms (except hydrogens at the γ-C of the β-diimine subunit) have been omitted for clarity. Selected bond lengths [Å] and angles [°]: Ni1···Ni2 4.3141(5), Ni1–N1 2.043(2), Ni1–N3 2.008(3), Ni1–N4 2.075(2), Ni1–C41 2.122(3), Ni1–C42 2.152(3), C41–C42 1.403(5), Ni2–N2 1.895(3), Ni2–N5 1.878(3), Ni2–N6 1.898(2), Ni2–N7 1.934(2); N3–Ni1–N1 81.10(10), N3–Ni1–N4 88.00(10), N1–Ni1–N4 144.40(10), N3–Ni1–C41 162.03(12), N1–Ni1–C41 109.50(11), N4–Ni1–C41 90.75(11), N3–Ni1–C42 124.60(12), N1–Ni1–C42 113.12­(11), N4–Ni1–C42 100.99(11), C41–Ni1–C42 38.32(12), N5–Ni2–N2 84.44(11), N5–Ni2–N6 93.27(11), N2–Ni2–N6 176.04(11), N5–Ni2–N7 164.62(11), N2–Ni2–N7 85.62(10), N6–Ni2–N7 97.27(10).

The electronic structure of **5** proposed on the basis of the crystallographic data with an *S* = 1/2 ground state was confirmed by EPR spectroscopy and SQUID magnetometry (Figures 10 and S18). The X-band EPR spectrum of crystalline **5** recorded at room temperature shows a rhombic signal with *g* = [2.010, 2.186, 2.294]. The value *g*_av_ = 2.16 and the *g* anisotropy are significantly larger than for **3** ^**N2**^, indicative of a mostly metal-based radical and typical for Ni^I^ (d^9^) complexes with T-shaped pincer-type ligation.^55,56^

**Figure 10:**
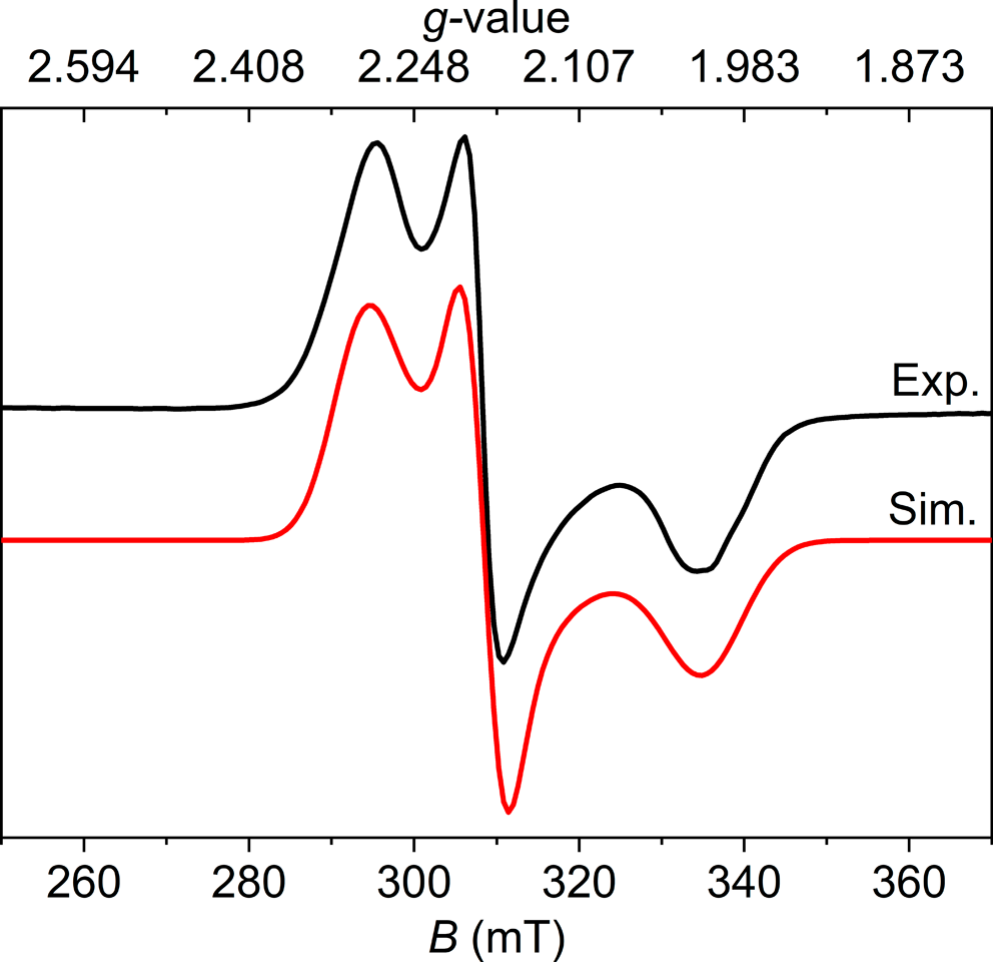
X-band EPR spectrum (9.438 GHz, microwave power 9.88 mW) of crystalline **5** at room temperature. Experimental data is displayed as a black line, while the simulated spectrum is reported in red with *g* = [2.010, 2.186, 2.294].

## Conclusions

Here we describe the unusual dinickel complex **3^N2^** that hosts a reductively activated N_2_ substrate in a highly constrained and extremely bent μ_1,2_-bridging mode within the bimetallic cleft. Spectroscopic and computational evidence suggests that the electronic structure of **3^N2^** is best described as Ni^II^–(N_2_^•–^)–Ni^II^ with predominant spin density on the bound N_2_; this clearly differs from the situation in linear VII featuring a Ni^I^–(N_2_^•–^)–Ni^I^ core, where the three-coordinate Ni retains its +I oxidation state. We attribute the unusual electronic structure of **3^N2^** to the bis­(tridentate) ligand scaffold L^3–^ enforcing a square planar coordination environment with a preferred d^8^ metal ion configuration in its dinuclear complexes that host a substrate within the bimetallic pocket. Consequently, the related neutral complex [LCo_2_(N_2_)] complex is best described as a mixed-valent [LCo^I^Co^II^(N_2_)] with significantly less activated N_2_ substrate (*v*_~NN_ = 1953 cm^–1^ vs 1894 cm^–1^ for **3^N2^**).^31^

Beyond these unique molecular dinickel complexes **VII** and **3^N2^**, well characterized systems with singly reduced N_2_ are very rare. An N_2_^•–^ radical absorbed on MgO/CaO at low temperatures has been observed by EPR spectroscopy after N_2_ reduction by trapped surface electrons,^57^ and a linear bridging N_2_^•–^ radical intermediate has recently been detected during photocatalytic nitrogen fixation at a zinc-based coordination polymer, where unsaturated [Zn^2+^···Zn^+^] sites are proposed to sequester the N_2_ substrate in a Zn^II^–(N_2_^•–^)–Zn^II^ motif.^58^ It remains to be demonstrated how the radical character of the activated and constrained N_2_ opens up new reactivity.

Starting from the dihydride complex **1** ^**K**^, binding of N_2_ to give **3** ^**N2**^ has been achieved via oxidation, either electrochemically or chemically with ferrocenium or H^+^ (which is accompanied by the release of 1 or >1 equiv of H_2_, respectively). Such coupling of N_2_ binding to the reductive release of H_2_ bears some reminiscence of the key step of N_2_ fixation by nitrogenase. In the present case, however, the redox-mediated process has uncovered a very unusual and counterintuitive scenario of N_2_ activation, viz., only the oxidation of a dinickel­(II) dihydride (or of a reduced dinickel­(I) scaffold) triggers the reductive binding of the N_2_ substrate. These findings may be thought-provoking for our detailed understanding of the multi-e^–^/H^+^ scenarios of biological N_2_ fixation via FeMoco dihydride intermediates, and also for the development of synthetic platforms that enable smooth N_2_ activation while avoiding strongly reducing conditions.

On the other hand, the release of N_2_ from **3** ^**N2**^ is triggered by both oxidation and reduction, the latter representing an interesting case of redox-induced electron transfer (RIET)^59^ where metal reduction leads to oxidation of the bound N_2_^•–^ due to intramolecular back electron transfer onto the dinickel framework. This is likely because the resonance energy resulting from partial electron delocalization over the Ni–(μ_1,2_-N_2_)–Ni core in **3** ^**N2**^ is lost upon reduction, and because of the preference of Ni^I^ (d^9^) complexes for T-shaped 3-fold coordination.^30,55,56^ In contrast, for the {LCo_2_} platform with bound N_2_, the reduced [LCo_2_^I^(N_2_)]^−^ has two square planar low-spin Co^I^ (d^8^) ions and hence is the most stable form.^31^ The present findings indicate, however, that only a narrow potential/p*K*_a_ window is available for further functionalization of the N_2_^•–^ ligand in **3** ^**N2**^ via proton-coupled electron transfer (PCET) reagents. Studies in that direction are ongoing in our laboratories.

## Supplementary Material



## Notes

Synthetic procedures and materials; NMR, IR, Raman, EPR, and ESI mass spectra; additional (spectro)­electrochemical data; detailed crystallographic information; details about DFT calculations. (PDF)

## ∥

Key Laboratory for Special Functional Materials of Ministry of Education, National and Local Joint Engineering Research Center for High-Efficiency Display and Lighting Technology, School of Nanoscience and Materials Engineering, Henan University, Kaifeng 475004, China
