# Supplementary material for: Oxidatively Induced Reductive N2 Binding: A Dinickel-Bridging Bent N2 Radical Anion and Its Redox-Triggered N2 Release
Source: J Am Chem Soc. 2025 Sep 6;147(37):33679–90. doi: 10.1021/jacs.5c09334 (PMC12447506; doi:10.1021/jacs.5c09334)
Supplement: Supplementary file 1 [file ja5c09334_si_001.pdf]

Supporting Information for

Oxidatively Induced Reductive N_2 Binding: a Dinickel-Bridging Bent N_2 Radical Anion and its Redox-Triggered N_2 Release

Sara I. Mozzi,[a] Dennis-Helmut Manz,[a] Nils Ostermann,[a] Roland A. Schulz,[a] Peng-Cheng Duan,[a,†] Thomas Kothe,[a] Martin Diefenbach,[b] Sebastian Dechert,[a] Serhiy Demeshko,[a] Vera Krewald,[b] Inke Siewert,[a,c] Franc Meyer[a,c]*

[a] University of Göttingen, Institute of Inorganic Chemistry, Tammannstr. 4, D-37077 Göttingen, Germany

[b] TU Darmstadt, Department of Chemistry, Quantum Chemistry, 64287 Darmstadt, Germany

[c] International Center for Advanced Studies of Energy Conversion (ICASEC), Tammannstr. 4, Universität Göttingen, D-37077, Germany

[†] present address: Henan University, Key Laboratory for Special Functional Materials of Ministry of Education, National and Local Joint Engineering Research Center for High-Efficiency Display and Lighting Technology, School of Nanoscience and Materials Engineering, 475004, Kaifeng, China

* Corresponding Author:
E-Mail: franc.meyer@chemie.uni-goettingen.de

Table of Contents

1. Materials and Methods S2
2. Synthetic Procedures and Analytical Data of Complexes, DFT Calculations S7
3. Additional Data S45
4. References S55

1. Materials and Methods

1.1 General Considerations

Manipulations involving air- and moisture-sensitive compounds were conducted under an atmosphere of dried (phosphorus pentoxide on solid support [Sicapent, Merck]) argon or nitrogen using standard Schlenk techniques, or in a N_2-filled MBraun glovebox or Ar-filled GS glovebox. Toluene and hexane were dried with an MBraun Solvent Purification System (SPS). THF and pentane were dried over sodium in presence of benzophenone. Deuterated solvents were dried over potassium sodium alloy and vacuum distilled. All the solvents were stored over molecular sieves (3 Å). All reagents used were purchased from commercial suppliers and used without further purification or synthesized according to reported literature as for lutidinium salts ([HLut]OTf and [HLut]BF_4).[1,2] NMR spectroscopy experiments of air- and moisture-sensitive compounds were conducted in J. Young NMR tubes.

1.2 Nuclear Magnetic Resonance Spectroscopy

^{1}H and ^{13}C{^{1}H} NMR spectra were recorded on Bruker Avance III 300 or Bruker Avance III 400 NMR spectrometers with THF-d_8 as solvent at 25 °C unless otherwise stated. The ^{1}H NMR spectra were calibrated against the residual ^{1}H and natural-abundance resonances of the deuterated solvent (THF-d_8 δ_H = 1.73 ppm).

1.3 Infrared Spectroscopy

IR spectra of solids were recorded on an Agilent Technologies Cary 630 ATR-FTIR spectrometer with Dial Path Technology and analyzed with FTIR MicroLab software. The position of the bands is given in wavenumbers (cm^{-1}) and the following abbreviations were applied for the intensity of band: s (strong), m (medium) and w (weak).

1.4 UV–vis Spectroscopy

UV–vis spectra were recorded with an Agilent Cary 60 spectrometer in a 1 cm path quartz Schlenk cuvettes or inside a MBraun glovebox with 0.2 cm path cuvettes with an Avantes AvaSpec-ULS2048L-StarLine spectrometer and an Avantes AvaLight-DH-S-BAL light source.

1.5 Raman Spectroscopy

Raman spectra of compounds were recorded using a HORIBA Scientific LabRAM HR 800 (400-1100 nm) spectrometer with open-electrode CCD detector and a confocal pinhole with user controlled variable aperture in combination with a free space optical microscope, and a He:Ne-laser (633 nm) or diode laser (457 nm). All spectra were recorded at room temperature. Raman measurements were performed under air at room temperature with crystalline sample mounted on a glass slide.

1.6 Electron Spin Resonance

EPR spectra were measured with a Bruker E500 ELEXSYS X-band spectrometer equipped with a standard cavity (ER4102ST, 9.45 GHz). The sample temperature was maintained constant with an Oxford instrument nitrogen flow cryostat (ESP910) and an Oxford temperature controller (ITC-4). The microwave frequency was measured with the built-in frequency counter, and the magnetic field was calibrated by using an NMR field probe (Bruker ER035M). EPR spectra were simulated using EasySpin.[3]

1.7 Magnetic Measurements

Temperature-dependent magnetic susceptibility measurements were carried out with a *Quantum-Design* MPMS3 (for **4^{PMe3}**) or a MPMS-XL-5 (for **5**) SQUID magnetometer in the range from 295 to 2.0 K at a magnetic field of 0.5 T. The powdered sample was contained in a polycarbonate capsule (for **4^{PMe3}**) or a Teflon bucket (for **5**) and fixed in a non-magnetic sample holder. Each raw data file for the measured magnetic moment was corrected for the diamagnetic contribution of the polycarbonate capsule or Teflon bucket according to M^{dia}(bucket) = $\chi_g \cdot m \cdot H$, with an experimentally obtained gram susceptibility of the polycarbonate capsule or Teflon bucket. The molar susceptibility data were corrected for the diamagnetic contribution using Pascal's constants (for **4^{PMe3}**) or according to χ_M^{dia}(sample) = $-0.5 \cdot M \cdot 10^{-6}$ cm^3·mol^{-1} (for **5**). Temperature-independent paramagnetism (*TIP*) was included according to $\chi_{calc} = \chi + TIP$. Before simulation, the experimental data were corrected for $TIP = -110 \cdot 10^{-6}$ cm^{3}mol^{-1} (for **4^{PMe3}**) or $TIP = 80 \cdot 10^{-6}$ cm^{3}mol^{-1} (for **5**). The negative sign for *TIP* of **4^{PMe3}** may indicate the slight underestimation of diamagnetic correction. Experimental data for **5** were modelled with the *julX* program[4] using a fitting procedure to the spin Hamiltonians:

$$\hat{H} = g\mu_B \vec{B} \cdot \vec{S} \qquad \text{(Eq. S1)}$$

Intermolecular interactions were considered in a mean field approach by using a Weiss temperature Θ.[5] The Weiss temperature $\Theta = -0.1$ K (defined as $\Theta = zJ_{inter}S(S+1)/3k$) relates to intermolecular interactions zJ_{inter}, where J_{inter} is the interaction parameter between two nearest neighbour magnetic centers, k is the Boltzmann constant (0.695 cm^{-1} K^{-1}) and z is the number of nearest neighbours.

1.8 Gas Chromatography

H_2 detection/quantification was performed with a Shimadzu GC-2014 gas chromatograph equipped with a thermal conductivity detector and a molecular sieves column (5 Å 80/100). Argon was used as carrier gas.

1.9 Electrochemical Measurements

Electrochemical measurements were performed in N_2 or Ar filled gloveboxes using Gamry Instruments Reference 600 or Reference 600+. The solvent and electrolyte dependent ohmic resistance drop (i*R*) was compensated by the positive feedback method, which is implemented in the Gamry PHE200 software. Measurements were performed with 0.2 M ^{n}Bu$_4$NPF$_6$ in dry THF solutions.

The electrolyte was purchased in electrochemical grade purity and dried for at least 3 days at 150 °C in *vacuo* before use. If not otherwise stated, all potentials are reported against the $Fc^{+|0}$ couple.

A three-electrode setup was used with a glassy carbon (GC, ALS Japan, 3 mm diameter) working electrode and a Pt-wire as a counter electrode. A separate compartment Ag-wire in a fritted sample holder with electrolyte solution was used as a pseudo reference electrode. After each measurement, ferrocene was added as an internal reference if not already present in the reaction mixture.

It was found that $\mathbf{1^K}$ upon dissolving in THF containing a high concentration of electrolyte (0.2 M $^n Bu_4NPF_6$ or 0.2 M $^n Bu_4NBAr^F_4$ in dry THF) readily loses H_2 and converts to $\mathbf{2^K}$. Hence, the CVs obtained after dissolving $\mathbf{1^K}$ or $\mathbf{2^K}$ in THF/0.2 M $^n Bu_4NPF_6$ are identical.

1.10 IR-Spectroelectrochemistry

IR-SEC measurements were performed using a CaF_2 windowed OTTLE cell[6] with platinum mesh working and counter electrodes and a silver pseudo reference electrode. The IR-SEC cell was prepared with 0.2 mL of THF solution (0.2 M $TBAPF_6$, 5 mM complex) under N_2 atmosphere. The sealed cell was prepared inside a glovebox and subsequently transferred outside the glovebox to be placed in the IR spectrometer and connected to the potentiostat for the measurements. A CV with scan rate of 2 mV/s was performed while recording an IR spectrum every 10 seconds. The potential was applied using Gamry Interface 1010E potentiostat without resistance correction and the IR spectra were recorded using a Bruker Vertex 70 spectrometer and evaluated with the software OPUS 7.8.

1.11 Computational Details

The isolated dinickel complexes $\mathbf{2}^-$, **3**, $\mathbf{4}^+$ and the corresponding dinickel dinitrogen complexes $\mathbf{2^{N2-}}$, $\mathbf{3^{N2}}$, $\mathbf{4^{N2+}}$ weres studied computationally at the density functional theory (DFT) level employing Grimme's r2SCAN-3c[7] composite scheme which makes use of the meta-generalized-gradient approximation (mGGA) and a specifically tailored valence triple-zeta basis set (mTZVPP),[7] the D4 London dispersion correction,[8] and a geometrical counterpoise (gCP) correction[9] to account for inter- and intramolecular basis set superposition errors (BSSE). Geometry optimizations and harmonic frequency calculations for all energetically relevant spin multiplicities were performed with the ORCA[10,11] program package under standard gas-phase conditions at 298.15 K and 1 atm adopting the standard DefGrid2 integration grid in conjunction with tight convergence criteria (VeryTightSCF, VeryTightOpt).

Molecular properties, relative energies, hyperfine coupling (HFC) parameters and g-tensor components were obtained with the PBE0[12,13] hybrid density functional with Grimme's atom-pairwise dispersion correction D3BJ[14] in conjunction with the scalar relativistic zeroth-order regular approximation (ZORA)[15,16] including picture-change effects, and an effective spin-orbit mean field operator.[17] The relativistically re-contracted ZORA-def2-QZVPP basis sets were used together with the corresponding SARC/J auxiliary Coulomb fitting basis sets,[18–20] employing the RIJCOSX

approximation[21] for the Coulomb and exchange terms, and the high-density integration grid DefGrid3. Natural bond orbital (NBO) analysis was performed on these wavefunctions with the NBO7[22] program.

1.12 X-Ray Crystallography

Crystal data and details of the data collections are given in Table S1, molecular structures are shown in Figure S1, Figure S12 and Figure S15. X-ray data were collected on a STOE IPDS II or a BRUKER D8-QUEST diffractometer (monochromated Mo-Kα radiation, λ = 0.71073 Å) by use of ω or ω and φ scans at low temperature. The structures were solved with SHELXT and refined on F^2 using all reflections with SHELXL.[23,24] Non-hydrogen atoms were refined anisotropically. Hydrogen atoms were placed in calculated positions and assigned to an isotropic displacement parameter of 1.5/1.2 U_{eq}(C).

One THF solvent molecule was found to be disordered in **5** (occupancy factors: 0.580(12) / 0.420(12)). SAME, RIGU and DELU restraints were applied to model the disordered parts. One THF solvent molecule was found to be disordered in $\mathbf{3^{N2}}$. The THF is disordered about a center of inversion and the carbon atoms were refined at ½ occupancy; the oxygen atom showed an additional positional disorder and was refined 0.35/0.15 occupancy. DFIX ($d(H_2C–CH_2)$ = 1.51 Å, d(C–O) = 1.43 Å) restraints were applied to model the disordered parts. Face-indexed absorption corrections were performed numerically with the program X-RED[25] or by the multi-scan method with SADABS.[26]

CCDC 2367279-2367281 contains the supplementary crystallographic data for this paper. This data can be obtained free of charge from The Cambridge Crystallographic Data Centre *via* www.ccdc.cam.ac.uk/structures.

Table S1. Crystal data and refinement details.

**compound**	**3^{N2}**	**4^{PMe3}**	**5**
empirical formula	$C_{41}H_{57}N_8Ni_2O_{0.50}$	$C_{49}H_{79}F_6N_6Ni_2OP_3$	$C_{58}H_{87}BF_4N_7Ni_2O_3$
moiety formula	$C_{39}H_{53}N_8Ni_2$, 0.5(C_4H_8O)	$C_{49}H_{79}F_6N_6Ni_2OP_3$	$C_{46}H_{63}N_7Ni_2^+$, BF_4^-, 3(C_4H_8O)
formula weight	787.36	1092.51	1134.57
T [K]	133(2)	100(2)	133(2)
crystal size [mm³]	0.310 x 0.280 x 0.240	0.335 x 0.278 x 0.157	0.500 x 0.180 x 0.110
crystal system	monoclinic	triclinic	triclinic
space group	$P2_1/c$ (No. 14)	P–1 (No. 2)	P-1 (No. 2)
a [Å]	17.1250(4)	13.0706(19)	13.3610(4)
b [Å]	14.2676(3)	14.159(2)	13.7556(5)
c [Å]	17.2804(4)	15.719(2)	16.8590(6)
α [°]	90	96.523(5)	74.184(3)
β [°]	112.654(2)	94.604(5)	73.891(3)
γ [°]	90	113.114(4)	79.539(3)
V [Å³]	3896.42(16)	2633.2(7)	2845.26(18)
Z	4	2	2
ρ [$g \cdot cm^{-3}$]	1.342	1.378	1.324
F(000)	1676	1156	1210
μ [mm^{-1}]	1.008	0.869	0.725
T_{min} / T_{max}	0.4572 / 0.5967*	0.72 / 0.88$	0.9829 / 0.9947*
θ-range [°]	1.915 - 26.816	2.010 - 27.103	1.293 - 26.846
hkl-range	±21, ±18, ±21	±16, ±18, ±20	–16 to 15, ±17, ±21
measured refl.	53170	60886	38213
unique refl. [R_{int}]	8265 [0.0276]	11599 [0.0996]	12075 [0.0486]
observed refl. ($I > 2\sigma(I)$)	7202	8281	8919
data / restr. / param.	8265 / 5 / 508	11599 / 0 / 622	12075 / 110 / 736
goodness-of-fit (F^2)	1.039	1.080	1.068
$R1$, $wR2$ ($I > 2\sigma(I)$)	0.0243 / 0.0607	0.0809 / 0.1938	0.0499 / 0.1222
$R1$, $wR2$ (all data)	0.0314 / 0.0642	0.1144 / 0.2128	0.0778 / 0.1380
res. el. dens. [$e \cdot Å^{-3}$]	–0.180 / 0.365	–1.257 / 1.541	–0.508 / 0.698

*) X-RED. $) SADABS.

2. Synthetic Procedures and Analytical Data of Complexes, DFT Calculations

2.1 Preparation of $LNi_2(\mu_{1,2}\text{-}N_2)$ (3^{N2})

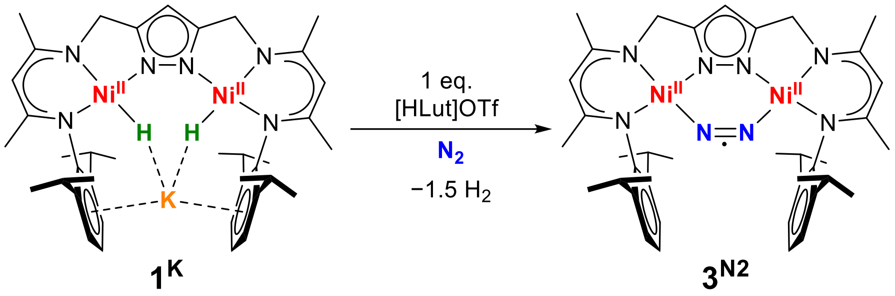

First procedure: Complex $\mathbf{1^K}$ (0.318 g, 0.400 mmol, 1.00 eq.) and [HLut]OTf (64.5 mg, 0.400 mmol, 1.00 eq.) were dissolved in THF (2 mL), and the reaction mixture were stirred for 2 h at room temperature under an atmosphere of dry dinitrogen. All volatiles were subsequently removed under vacuum, and the resulting solid was dried under vacuum. Layering hexane onto a solution of the crude product in THF at –30°C yielded block shaped crystals. The molecular structure of $\mathbf{3^{N2}}$ could be obtained by XRD analysis of selected crystals. However, ^{1}H NMR spectroscopy analysis of the bulk crystalline material revealed significant (though variable) amounts of the degradation side product $LNi_2(\mu\text{-}OH)$. For this reason, the yield in $\mathbf{3^{N2}}$ of the reaction cannot be provided.

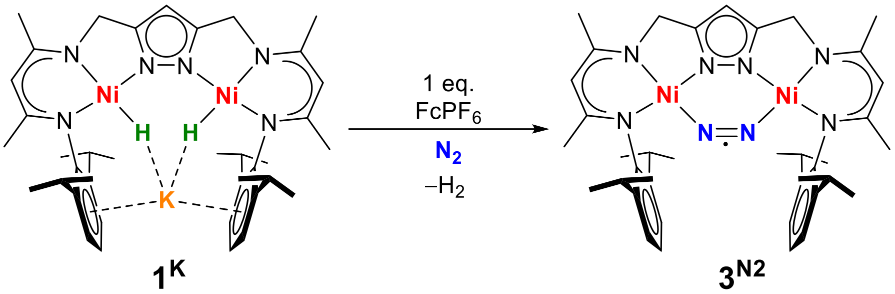

Second procedure: A solution of $\mathbf{1^K}$ (7.00 mg, 9.16 µmol, 1.00 eq.) in THF (1 mL) was added to $FcPF_6$ (3.03 mg, 9.16 µmol, 1.00 eq.) under dinitrogen atmosphere and vigorous stirring. The solution immediately changed color from dark red to dark brown with gas evolution. ^{1}H NMR analysis indicated essentially quantitative formation of the product $\mathbf{3^{N2}}$, but separation of $\mathbf{3^{N2}}$ from ferrocene proved difficult, and crystallization by layering hexane onto the THF reaction mixture solution at –30 C resulted in partial decomposition to $LNi_2(\mu\text{-}OH)$. The characterization of $\mathbf{3^{N2}}$ and reactivity studies were therefore carried out on the *in situ* generated $\mathbf{3^{N2}}$.

IR [cm^{-1}]: $\tilde{\nu}$ = 3056 (w), 2954 (m), 2924 (m), 2865 (m), 1894 ν_{N2} (m), 1551 (s), 1529 (s), 1455 (s), 1435 (s), 1396 (s), 1360 (w), 1317 (w), 1310 (m), 1277 (w), 1251 (w), 1232 (w), 1187 (w), 1092 (w), 1053 (w), 1031 (w), 1010 (w), 915 (w), 865 (w), 844 (s) 792 (s), 755 (s), 746 (s).

Raman [cm^{-1}]: $\tilde{\nu}$ = 1898 (ν_{14N2}).

UV-vis (THF) [nm]: λ = 370, 430, 495, 700.

EPR (129 K, 9.429 GHz, toluene, X-band): g = [1.994, 2.070, 2.149], $A(^{14}N)$ = [36, 58, 33] MHz. (290 K, 9.427 GHz, toluene, X-band): g = 2.075 $A(^{14}N)$ = 43 MHz.

Molecular Structure Determined by X-Ray Diffraction

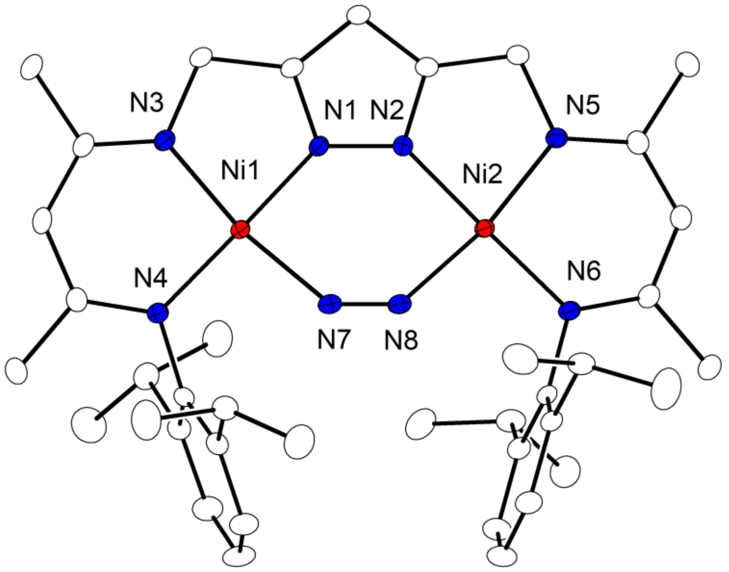

Figure S1. Plot (30% probability thermal ellipsoids) of the molecular structure of $\mathbf{3^{N2}}$ (hydrogen atoms omitted for clarity). Selected bond lengths [Å] and angles [°]: Ni1–N1 1.8650(12), Ni2–N2 1.8737(12), Ni1–N3 1.8859(11), Ni1–N4 1.8978(12), Ni2–N5 1.8988(12), Ni2–N6 1.9073(12), Ni1–N7 1.8844(13), Ni2–N8 1.8828(13), N7–N8 1.1324(18), Ni1···Ni2 3.9621(5); N1–Ni1–N7 87.07(5), N1–Ni1–N3 83.80(5), N7–Ni1–N3 169.39(5), N1–Ni1–N4 175.35(5), N7–Ni1–N4 94.54(5), N3–Ni1–N4 94.98(5), N2–Ni2–N8 87.37(5), N2–Ni2–N5 83.13(5), N8–Ni2–N5 170.35(5), N2–Ni2–N6 178.25(5), N8–Ni2–N6 94.01(5), N5–Ni2–N6 95.51(5), N8–N7–Ni1 139.43(11), N7–N8–Ni2 137.93(11).

IR, Raman, UV-Vis and EPR Spectroscopic Data

Sample preparation for IR and Raman spectroscopy: **3**^{N2} was prepared as described above, according to the second procedure (5 mg of **1**^{K} and 0.5 mL of THF scale reaction). The reaction mixture was subsequently cooled to −35 °C for 20 minutes. Solid **3**^{N2} precipitated out of the solution, the solvent was decanted and the solid dried. Contamination of the product by KPF_6 salts is expected. The UV-Vis spectra were recorded after re-dissolving the solid in THF.

Sample preparation for labelling experiment. Important remarks: the reaction and the work-up of the reaction were performed in an argon filled glovebox to prevent contamination and exchange of $^{15}N_2$ with $^{14}N_2$. The $^{15}N_2$ gas was dried in a young flask over molecular sieves (3 Å) for at least 3 days prior usage.

The reaction of **1**^{K} with Fc (5 mg of **1**^{K} and 0.5 mL of THF scale reaction), was performed in a Schlenk tube in an argon filled glovebox. The tube was transferred outside of the glovebox and connected to a Schlenk line and to a $^{15}N_2$ containing young flask via a three-way valve. The reaction mixture was frozen with liquid nitrogen and vacuum applied. The headspace was filled with $^{15}N_2$ and the tube allowed to reach room temperature. The Schlenk tube was transferred into a glovebox. The work-up was performed following the standard procedure as for the $^{14}N_2$ labeled samples.

Sample preparation for EPR spectroscopy: **3**^{N2} was prepared as described above, according to the second procedure (5 mg of **1**^{K} and 0.5 mL of THF scale reaction). The solvent was removed, the residue re-dissolved in toluene and diluted to a suitable concentration.

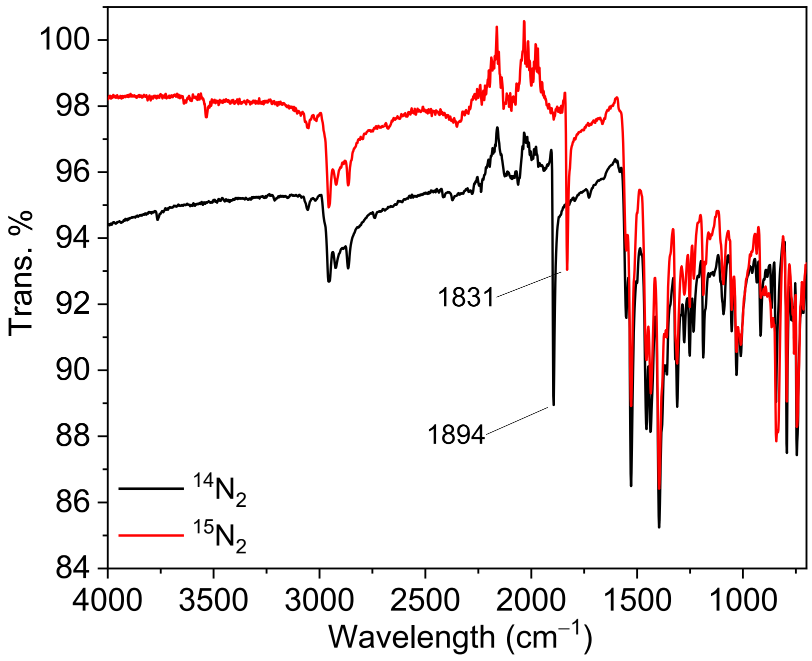

Figure S2. ATR-IR spectrum of solid **3**^{N2} (black line) and $^{15}N_2$ labelled **3**^{N2} (red line).

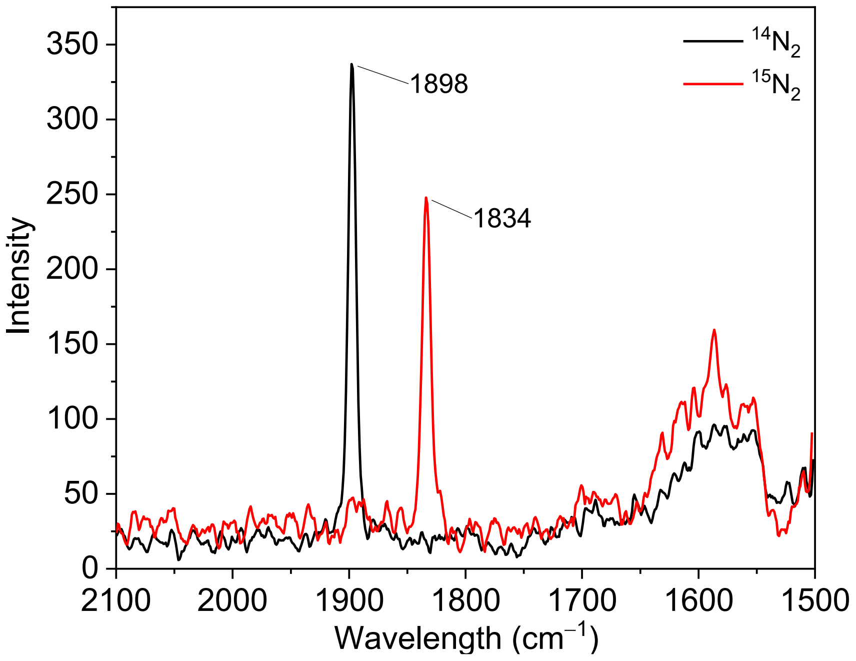

Figure S3. rRaman spectrum of solid $\mathbf{3}^{\mathbf{N2}}$ (black line) and $^{15}N_2$ labelled $\mathbf{3}^{\mathbf{N2}}$ (red line). Baseline is corrected using the software SpectraGryph 1.2.[27]

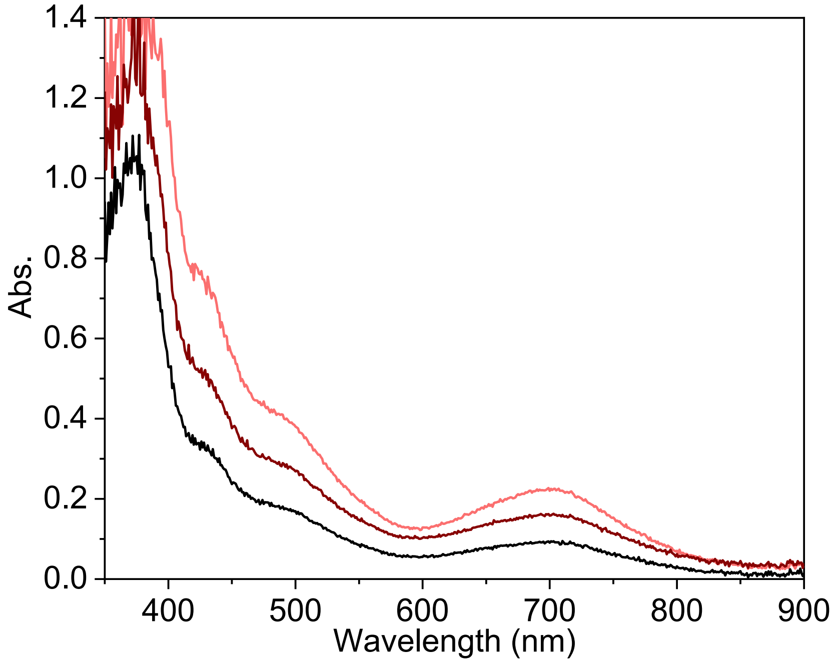

Figure S4. UV-vis spectra of $\mathbf{3}^{\mathbf{N2}}$ in THF at room temperature at different concentrations (0.49, 0.66 and 0.99 mM from black to light red line). Extinction coefficients could not be calculated due to the presence of KPF_6 salts.

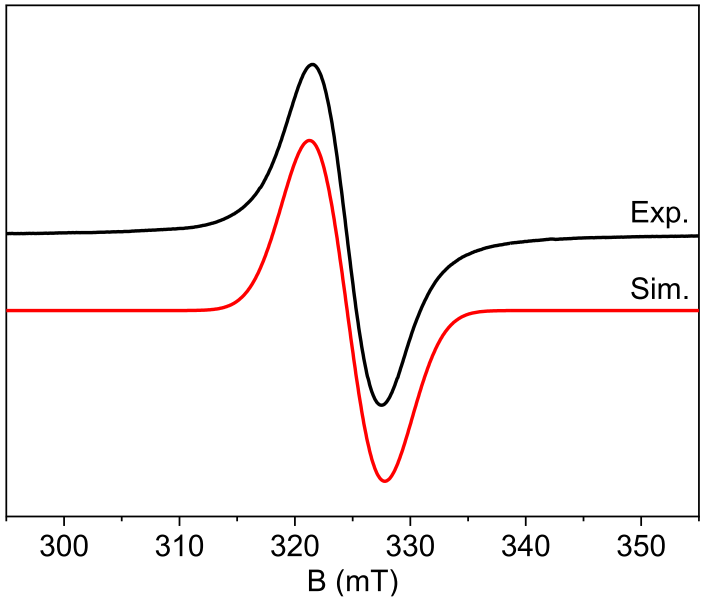

Figure S5. X-band EPR spectrum (9.426 GHz) of $\mathbf{3}^{\mathbf{N2}}$ in toluene (2.5 mM) at 290 K. Experimental data is displayed as black line, while simulated spectrum is shown in red with $g = 2.075$, $A(^{14}N) = 43$ MHz.

DFT calculations

Species $\mathbf{3^{N_2}}$ possesses a doublet $S = 1/2$ ground state. A high-spin quartet isomer is $\Delta E_{\mathrm{tot}} = +18.6\,\mathrm{kcal\,mol^{-1}}$, $\Delta H^{298} = +16.8\,\mathrm{kcal\,mol^{-1}}$ and $\Delta G^{298} = +13.9\,\mathrm{kcal\,mol^{-1}}$ higher in energy than the doublet ground-state species (PBE0-D3BJ/ZORA-def2-QZVPP // r^{2}SCAN-3c). Alternative electronic structures based on broken-symmetry wavefunctions were probed, either starting from the quartet state or by using a dedicated fragment guess in which the {NiNNNi} bridge forms an α, β, α spin sequence, all of which collapse to the low-spin doublet configuration.

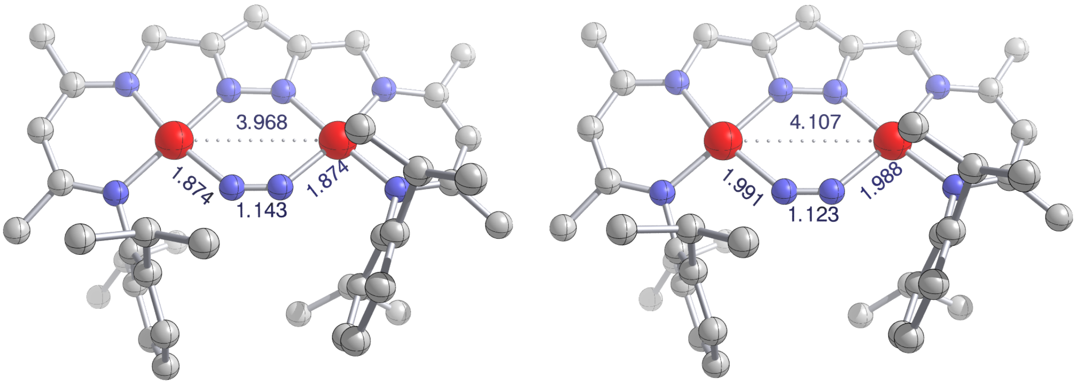

Figure S6: Molecular geometries for doublet ground-state $\mathbf{3^{N_2}}$ (left) and its quartet isomer (right), optimized at the r^{2}SCAN-3c level. Selected interatomic distances are given in Å.

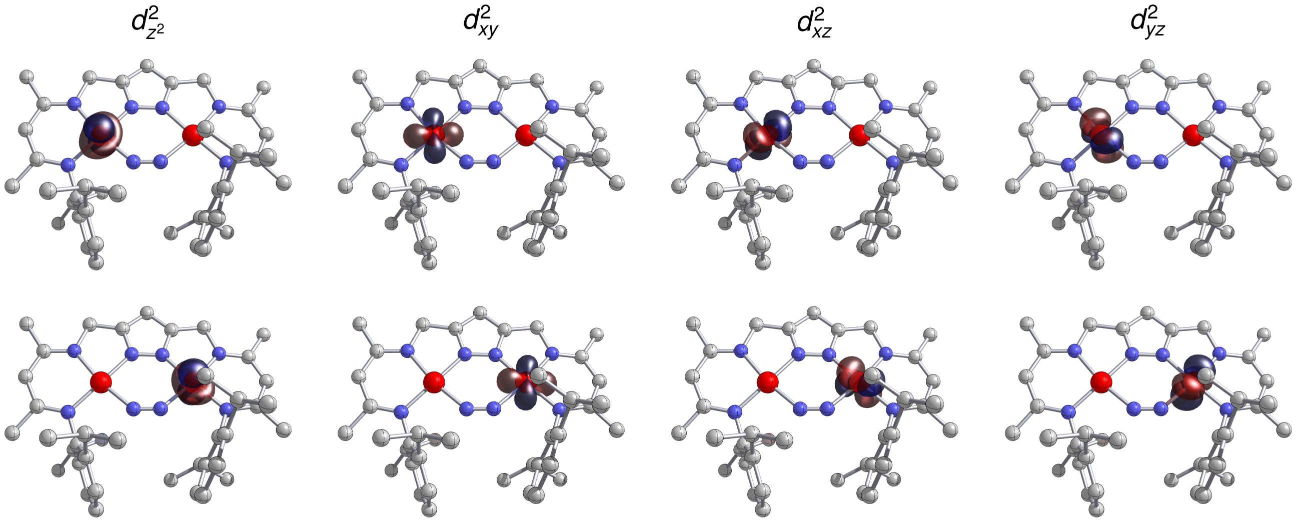

Figure S7: Doubly occupied Ni-centred d lone pair NLMOs from NBO analysis of $\mathbf{3^{N_2}}$ computed at the PBE0/ZORA-def2-QZVPP // r^{2}SCAN-3c level. Isosurfaces at $\pm 0.1\, a_0^{-3/2}$; only the α spin surface is shown.

Table S2: Computed hyperfine coupling (HFC) parameters A in MHz for N7 and N8, and g-tensor components for $\mathbf{3^{N_2}}$ in its $S = 1/2$ ground state, obtained at r^{2}SCAN-3c geometries using the PBE0 hybrid functional in conjunction with the relativisic ZORA-def2-SVP, ZORA-def2-TZVP, ZORA-def2-TZVPP, and ZORA-def2-QZVPP basis sets. Corresponding parameters extracted from EPR spectroscopy are given for comparison.

Method	A_x(^{14}N)	A_y(^{14}N)	A_z(^{14}N)	A_{iso}(^{14}N)[a]	g_x	g_y	g_z	g_{iso}
ZORA-def2-SVP	55	94	57	69	1.987	2.068	2.104	2.053
ZORA-def2-TZVP	33	72	36	47	1.994	2.066	2.117	2.059
ZORA-def2-TZVPP	32	71	35	46	1.994	2.063	2.116	2.057
ZORA-def2-QZVPP	30	70	33	45	1.993	2.062	2.117	2.057
X-band EPR (129 K)	36	58	33	42	1.994	2.070	2.149	2.071
X-band EPR (290 K)				43				2.075

[a]computed HFC parameters for N7 and N8 are equivalent due to symmetry.

Table S3: Spin expectation value $\langle S^2 \rangle$, total energy (E_{tot}), zero-point vibrational energy contribution (E^{ZPE}), thermal correction contributions to energy (U^{corr}), enthalpy (H^{corr}) and Gibbs energy (G^{corr}) for electromers of **3**, **2**$^-$, **4**$^+$, and $\mathbf{3^{N_2}}$, $\mathbf{2^{N_2}}^-$, $\mathbf{4^{N_2}}^+$. The total energies are composed of the sum of individual correction contributions, e.g. $H_{tot} = E_{tot} + E^{ZPE} + U^{corr} + H^{corr}$; $G_{tot} = E_{tot} + E^{ZPE} + U^{corr} + H^{corr} + G^{corr}$. Relative energies E_{rel}, enthalpies H_{rel} and Gibbs energies G_{rel} are given in kcal mol^{-1}.

Species	$\langle S^2 \rangle$	E_{tot}	E^{ZPE}	U^{corr}	H^{corr}	G^{corr}	E_{rel}	H_{rel}	G_{rel}
	PBE0-D3BJ/ZORA-def2-QZVPP // r^{2}SCAN-3c								
^{4}**3**	3.828	−4901.622 553	0.845 048	0.050 490	0.000 944	−0.126 378	0.0	0.0	0.0
^{2}**3**	1.190	−4901.619 599	0.845 586	0.050 290	0.000 944	−0.125 122	1.9	2.1	2.9
^{BS}**2**$^{-[a]}$	1.027	−4901.699 166	0.842 123	0.050 655	0.000 944	−0.125 233	0.0	0.0	0.0
^{3}**2**$^-$	2.027	−4901.695 942	0.842 842	0.050 451	0.000 944	−0.125 753	2.0	2.4	2.0
^{5}**2**$^-$	6.042	−4901.640 432	0.839 257	0.050 998	0.000 944	−0.127 491	36.9	35.3	33.9
^{1}**2**$^{-[b]}$	0.000	−4901.632 707	0.843 653	0.048 468	0.000 944	−0.120 455	41.7	41.3	44.3
^{3}**4**$^+$	2.360	−4901.452 218	0.846 815	0.050 330	0.000 944	−0.125 829	0.0	0.0	0.0
^{5}**4**$^+$	6.033	−4901.451 420	0.846 647	0.050 122	0.000 944	−0.125 680	0.5	0.3	0.4
^{1}**4**$^+$	0.000	−4901.447 844	0.847 957	0.050 023	0.000 944	−0.123 854	2.7	3.3	4.5
$^1\mathbf{N_2}^{[c]}$	0.000	−109.550 903	0.005 567	0.002 361	0.000 944	−0.021 735			
$^2\mathbf{3^{N_2}}$	0.876	−5011.203 234	0.856 969	0.051 683	0.000 944	−0.127 337	0.0	0.0	0.0
$^4\mathbf{3^{N_2}}$	3.981	−5011.173 653	0.852 599	0.053 317	0.000 944	−0.132 058	18.6	16.9	13.9
$^3\mathbf{2^{N_2}}^-$	2.033	−5011.256 364	0.850 652	0.053 336	0.000 944	−0.131 927	0.0	0.0	0.0
$^{BS}\mathbf{2^{N_2}}^{-[d]}$	0.976	−5011.256 774	0.851 636	0.052 668	0.000 944	−0.128 947	−0.3	−0.1	1.8
$^1\mathbf{2^{N_2}}^-$	0.000	−5011.227 485	0.853 172	0.051 970	0.000 944	−0.127 227	18.1	18.9	21.8
$^1\mathbf{4^{N_2}}^+$	0.000	−5011.012 455	0.859 096	0.051 642	0.000 944	−0.126 712	0.0	0.0	0.0
$^3\mathbf{4^{N_2}}^+$	2.031	−5011.005 441	0.856 773	0.052 274	0.000 944	−0.129 283	4.4	3.3	1.7
$^5\mathbf{4^{N_2}}^+$	6.038	−5010.992 246	0.853 921	0.053 503	0.000 944	−0.133 473	12.7	10.6	6.4

[a]broken-symmetry singlet with antiferromagnetically coupled Ni centers, $J = -43$ cm^{-1};
[b]2 imaginary modes $\nu_{imag} = 18.4\,i$ cm^{-1} and $17.9\,i$ cm^{-1};
[c]N–N distance: 1.094 Å;
[d]broken-symmetry singlet with antiferromagnetically coupled Ni centers, $J = -780$ cm^{-1}

Table S4: Binding of N_2 towards **3**, **2**$^-$, **4**$^+$ to yield $\mathbf{3}^{\mathbf{N_2}}$, $\mathbf{2}^{\mathbf{N_2}-}$, $\mathbf{4}^{\mathbf{N_2}+}$, respectively, computed at PBE0-D3BJ/ZORA-def2-QZVPP // r^{2}SCAN-3c. Relative energies are given in kcal mol^{-1}.

Valence	Reaction	$\Delta_r E^0$	$\Delta_r H^{298}$	$\Delta_r G^{298}$
$Ni^I Ni^{II}$	$^{4}\mathbf{3}$ + $^{1}\mathbf{N_2}$ $\longrightarrow$ $^{2}\mathbf{3}^{\mathbf{N_2}}$	−18.7	−16.0	−3.0
$Ni^I Ni^I$	$^{\mathbf{BSS}}\mathbf{2}^-$ + $^{1}\mathbf{N_2}$ $\longrightarrow$ $^{3}\mathbf{2}^{\mathbf{N_2}-}$	−4.0	−2.5	7.0
$Ni^{II} Ni^{II}$	$^{3}\mathbf{4}^+$ + $^{1}\mathbf{N_2}$ $\longrightarrow$ $^{1}\mathbf{4}^{\mathbf{N_2}+}$	−5.9	−2.9	10.2

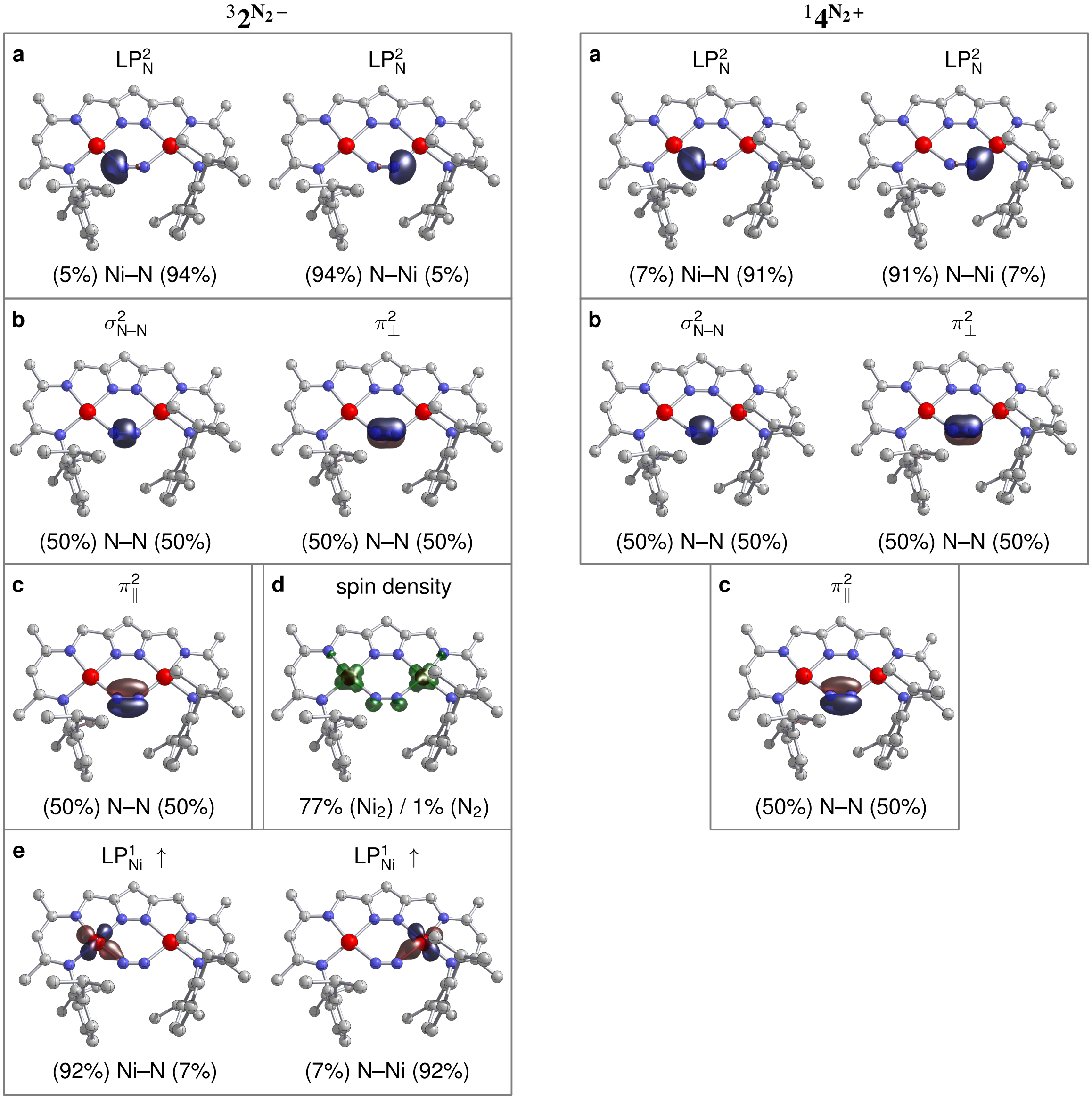

Figure S8: Selected NLMOs from NBO analysis of $Ni^I Ni^I$ species $^{3}\mathbf{2}^{\mathbf{N_2}-}$ (left) and the $Ni^{II} Ni^{II}$ singlet cation isomer $^{1}\mathbf{4}^{\mathbf{N_2}+}$ (right), at the PBE0/ZORA-def2-QZVPP // r^{2}SCAN-3c level. Isosurfaces at ±0.1 $a_0^{-3/2}$; for doubly occupied NLMOs the average of α and β spin is shown. a) doubly occupied N-centred lone pairs; b, c) doubly occupied N–N σ, π orbitals; d) spin density (isosurface at 0.01 a_0^{-3}) with NPA spin population contributions; e) singly occupied Ni $d_{x^2-y^2}$ orbitals (α spin).

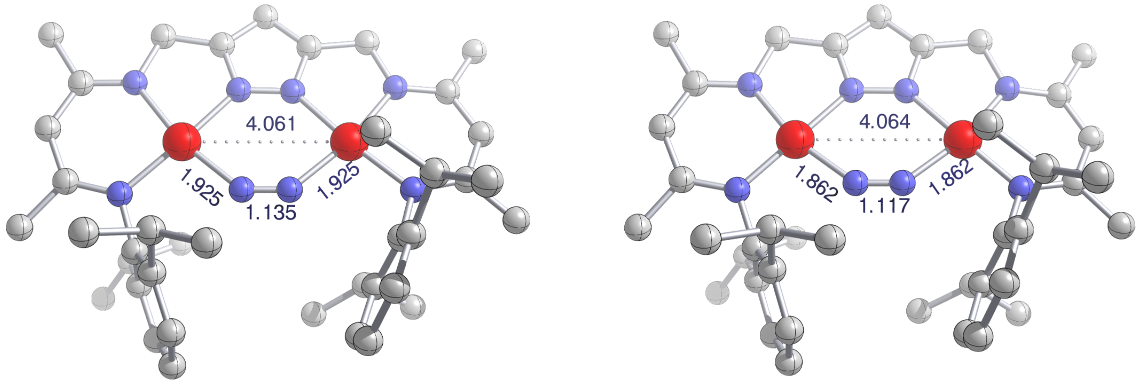

$^{3}\mathbf{2}^{\mathrm{N_2}-}$ $^{1}\mathbf{4}^{\mathrm{N_2}+}$

Figure S9: Molecular geometries for triplet anion dinitrogen complex $\mathbf{2}^{\mathrm{N_2}-}$ (left) and the singlet cation isomer $\mathbf{4}^{\mathrm{N_2}+}$ (right), optimized at the r^{2}SCAN-3c level. Selected interatomic distances are given in Å.

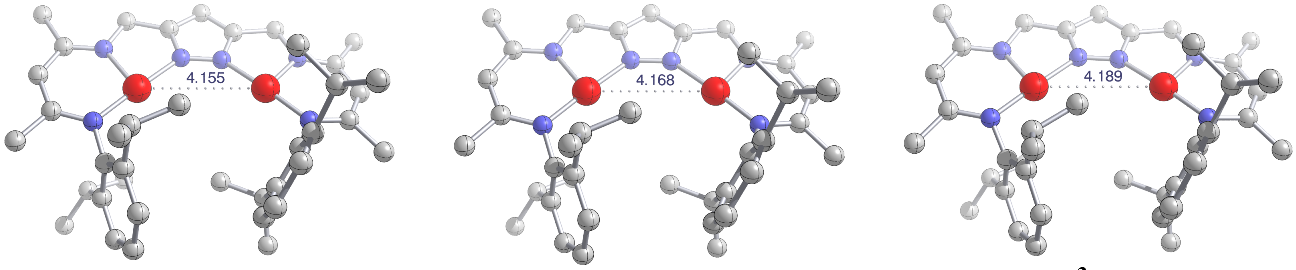

$^{4}\mathbf{3}$ $^{\mathrm{BSS}}\mathbf{2}^{-}$ $^{3}\mathbf{4}^{+}$

Figure S10: Molecular geometries for isolated dinickel complexes **3**, $\mathbf{2}^{-}$ and $\mathbf{4}^{+}$, optimized at the r^{2}SCAN-3c level. Ni$\cdots$Ni internuclear distances are given in Å.

Cartesian coordinates of optimized geometries (Å)

100

$^{2}\mathbf{3}$ E_{tot}(r^{2}SCAN-3c) = -4862.940026 $\langle S^2 \rangle$ = 0.954

Ni	-2.0370241272	-0.0000000000	0.0000000000
Ni	2.0370241272	0.0000000000	-0.0000000000
N	-0.6689562881	1.2552305092	-0.2870474031
N	-3.2158950457	1.4568542631	-0.1827145026
N	-3.3260995393	-1.2925227922	0.2600967781
N	0.6689483317	1.2309070112	-0.3779727969
N	3.2158890452	1.3535224269	-0.5690565607
N	3.3261171723	-1.2497740740	0.4199100977
C	-1.0961385280	2.4998089288	-0.5805634259
C	-2.5696741416	2.7292066464	-0.5151501874
C	-4.5168265744	1.4037416903	0.0534640682
C	-4.6327908741	-1.0746178654	0.4044202444
C	-2.8229702532	-2.6113044785	0.0956652797
C	1.0961273063	2.4558117533	-0.7450416534
C	-0.0000061457	3.3084841813	-0.8850070115
C	-5.1920636377	0.2059639624	0.3535322101
C	-5.3340453577	2.6727646979	-0.0000000000
C	-5.5468555965	-2.2546328946	0.6230064251
C	-2.2274749256	-3.2670334016	1.1943073220
C	-2.9164279532	-3.2360751413	-1.1689592029
C	2.5696600539	2.6219190624	-0.9162636832
C	-1.7376223031	-4.5585187241	1.0058906447
C	-2.1219493585	-2.5656353796	2.5349412761
C	-2.4169089808	-4.5297395677	-1.3048293347
C	-3.4874498888	-2.4892809207	-2.3599009969

C	4.5168135090	1.1895825999	-0.7472209921
C	4.6328038955	-1.1330160673	0.1860372907
C	2.8230281922	-2.3103449111	1.2208234922
C	-1.8357025107	-5.1893411420	-0.2281587749
C	-0.8696144037	-1.6776864645	2.5763590083
C	-2.1178732565	-3.5277458594	3.7260452745
C	-2.3655403441	-1.7244961665	-3.0806972052
C	-4.2421633953	-3.3903551689	-3.3414100759
C	5.1920591005	0.0019539364	-0.4092280923
C	5.3340167973	2.3158106500	-1.3345150766
C	5.5468877448	-2.2645615242	0.5857696201
C	2.9165021891	-2.2202722211	2.6284813826
C	2.2275545897	-3.4270406781	0.5963150869
C	2.4169905000	-3.2733170025	3.3921120630
C	3.4875222486	-0.9786013750	3.2874961809
C	1.7377152289	-4.4519646806	1.4043865473
C	2.1220448879	-3.4886921186	-0.9154598714
C	1.8357884549	-4.3823883758	2.7885737360
C	2.3656069634	0.0439099561	3.5301819926
C	4.2422472739	-1.2692761368	4.5877984245
C	0.8697190247	-2.7400137175	-1.3947041344
C	2.1179590820	-4.9170052706	-1.4671007633
H	-2.7918955973	3.5043273793	0.2360163736
H	-2.9355780013	3.1107152186	-1.4825882179
H	-0.0000054266	4.3521759492	-1.1641936715
H	-6.2576602608	0.2799538683	0.5323752792
H	-5.0001502133	3.3870932322	0.7619586362
H	-6.3902345567	2.4603043163	0.1713755775
H	-5.2355448591	3.1680172361	-0.9729705076
H	-5.6016079489	-2.8788899842	-0.2770380472
H	-6.5554061956	-1.9220794722	0.8745540667
H	-5.1699640057	-2.8970786447	1.4266081782
H	2.7918707977	2.9184779884	-1.9541076571
H	2.9355679072	3.4354925812	-0.2685134571
H	-1.2812204320	-5.0845091765	1.8380251633
H	-2.9974967434	-1.9098813324	2.6293641054
H	-2.4798538943	-5.0330579630	-2.2649892635
H	-4.1954962640	-1.7413099880	-1.9846290162
H	-1.4591323409	-6.2012574667	-0.3516764674
H	0.0342397287	-2.2715875314	2.4072061669
H	-0.7808549228	-1.1753525204	3.5462009245
H	-0.9107814525	-0.8896014666	1.8070002768
H	-1.1846555873	-4.0996552640	3.7750847647
H	-2.9539944434	-4.2336417716	3.6786248111
H	-2.1990574624	-2.9609107939	4.6592296619
H	-1.8485142904	-1.0455672059	-2.3894545567
H	-2.7722711639	-1.1252366043	-3.9031201655
H	-1.6275007321	-2.4229305659	-3.4914205883
H	-3.5670392370	-4.0632365147	-3.8815921912
H	-4.7526432964	-2.7768188346	-4.0911090345
H	-4.9934126875	-4.0026119038	-2.8308447305
H	6.2576504026	-0.0232257601	-0.6011575339
H	5.0000909834	2.5543017571	-2.3513474201
H	6.3902040417	2.0461591680	-1.3769580318
H	5.2355401899	3.2307057716	-0.7387644984
H	5.6017162153	-2.3560340312	1.6772831337
H	6.5554134611	-2.1020346470	0.2017075202
H	5.1699638348	-3.2224334551	0.2103126778
H	2.4799464592	-3.2300269585	4.4753305732
H	4.1955601103	-0.5178994676	2.5888877420
H	1.2813318919	-5.3231843384	0.9460125483
H	2.9975999379	-2.9676724193	-1.3246719209
H	1.4592222635	-5.1974827291	3.4008281100
H	1.8485479567	0.2869932358	2.5922923631
H	2.7723350069	0.9737724719	3.9435208144
H	1.6275953154	-0.3561679252	4.2347992074
H	3.5671287287	-1.5825824656	5.3917960120
H	4.7527285500	-0.3633711082	4.9310339162
H	4.9934976058	-2.0546732891	4.4511072308
H	-0.0341351807	-3.1700970483	-0.9515839035
H	0.7809405716	-2.7890516245	-2.4858154654
H	0.9109213269	-1.6730464507	-1.1216315632

H	2.9540705517	-5.5049524247	-1.0735620384
H	2.1991507690	-4.8918117960	-2.5586587735
H	1.1847312822	-5.4369949662	-1.2240468803

100

^{4}3 E_{tot}(r^{2}SCAN-3c) = -4862.945806 $\langle S^2 \rangle$ = 3.786

Ni	-2.0772563990	0.0000000000	-0.0000000000
Ni	2.0772563990	-0.0000000000	0.0000000000
N	-0.6801879226	1.2432158230	-0.2670977428
N	-3.2510210538	1.5157110031	-0.1471303773
N	-3.3835667669	-1.2857334914	0.3013850884
N	0.6801982562	1.2189058754	-0.3622076750
N	3.2510410304	1.4004358000	-0.5981036910
N	3.3835458201	-1.2726620182	0.3525771758
C	-1.0994928395	2.4939820024	-0.5467646218
C	-2.5722188862	2.7631371795	-0.4869535501
C	-4.5481464900	1.4778407875	0.0807747723
C	-4.6806429399	-1.0113408512	0.5010664004
C	-2.9402743147	-2.6389282345	0.2494185522
C	1.0995147474	2.4504950368	-0.7169065570
C	0.0000138483	3.3009343895	-0.8436104766
C	-5.2169627185	0.2770801023	0.4132209545
C	-5.3689886642	2.7422653625	0.0000000000
C	-5.6145696039	-2.1458754756	0.8458312696
C	-2.2590224565	-3.1904284043	1.3526402915
C	-3.1368090728	-3.3845642341	-0.9338336812
C	2.5722478499	2.6579478588	-0.8984859924
C	-1.7910528603	-4.5010960967	1.2558749025
C	-2.0056433795	-2.3640486710	2.5987140644
C	-2.6581904459	-4.6929809541	-0.9793874402
C	-3.7761887060	-2.7467505988	-2.1547241494
C	4.5481815995	1.2578696903	-0.7797934091
C	4.6806365088	-1.1277337217	0.0457894789
C	2.9401888639	-2.4349712401	1.0474235105
C	-1.9933295136	-5.2520953792	0.1057999011
C	-0.6248370975	-1.6950568910	2.5214395577
C	-2.1256682193	-3.1731225414	3.8942675657
C	-2.6882726197	-2.1040015325	-3.0291157452
C	-4.6257671074	-3.7185002551	-2.9786718293
C	5.2169837430	0.0448424204	-0.4953039251
C	5.3690608585	2.4060151459	-1.3155101306
C	5.6145416056	-2.2885752369	0.2876924643
C	3.1366687758	-2.5214612598	2.4433487663
C	2.2588964005	-3.4481283756	0.3440720437
C	2.6579477732	-3.6475443811	3.1110801400
C	3.7760970383	-1.3761101670	3.2085178099
C	1.7908144845	-4.5516116776	1.0578170657
C	2.0055774621	-3.3209560681	-1.1457129179
C	1.9930352510	-4.6587265102	2.4272036099
C	2.6882433034	-0.3925330175	3.6672141122
C	4.6255728199	-1.8334079539	4.3977375310
C	0.6246956270	-2.6971171017	-1.3989569204
C	2.1258493200	-4.6524043021	-1.8942114167
H	-2.7714530403	3.5528218669	0.2565635380
H	-2.9193195400	3.1561149773	-1.4575885728
H	0.0000155469	4.3482072469	-1.1112379569
H	-6.2790311633	0.3639408964	0.6098337975
H	-5.0300395858	3.4764654157	0.7408795368
H	-6.4255547223	2.5384706336	0.1801876851
H	-5.2720250844	3.2119798126	-0.9860086095
H	-5.7035108961	-2.8533710810	0.0135440547
H	-6.6096416686	-1.7709078189	1.0907237305
H	-5.2283694936	-2.7159019491	1.6986256555
H	2.7715101336	2.9940622633	-1.9297164159
H	2.9193305008	3.4684471863	-0.2354124250
H	-1.2604772188	-4.9420764967	2.0942140722
H	-2.7619041460	-1.5700479096	2.6275539240
H	-2.8004614613	-5.2852765636	-1.8785058731
H	-4.4341159195	-1.9399080439	-1.8138276396
H	-1.6301250552	-6.2748746590	0.0526409518
H	0.1694724748	-2.4476955125	2.4662527358
H	-0.4490471765	-1.0656840227	3.4008778754

H	-0.5376127562	-1.0486919076	1.6351584697
H	-1.3016239275	-3.8869218670	4.0044946577
H	-3.0676721698	-3.7303061298	3.9333429720
H	-2.0873277472	-2.5003709970	4.7576102668
H	-2.0981682841	-1.3802196182	-2.4536429423
H	-3.1345340842	-1.5804822435	-3.8822917762
H	-2.0054537237	-2.8718822268	-3.4108761310
H	-4.0116101490	-4.4709146229	-3.4852612772
H	-5.1698511918	-3.1704743661	-3.7552427025
H	-5.3567563346	-4.2431691790	-2.3539576778
H	6.2790637534	0.0267143763	-0.7094246754
H	5.0301810673	2.6947546411	-2.3178221560
H	6.4256292446	2.1407521532	-1.3757630128
H	5.2720640467	3.2911972074	-0.6757508194
H	5.7034358848	-2.5099908611	1.3573819525
H	6.6096321186	-2.0770929780	-0.1070373650
H	5.2283527802	-3.1978692030	-0.1870522574
H	2.8001714125	-3.7358196554	4.1841360509
H	4.4341078385	-0.8318392181	2.5223215289
H	1.2601828884	-5.3407060019	0.5338434906
H	2.7617574414	-2.6380390378	-1.5519303574
H	1.6297473125	-5.5305554706	2.9645649199
H	2.0981900010	-0.0336092604	2.8150036535
H	3.1345655385	0.4761326172	4.1645544553
H	2.0053673747	-0.8829918040	4.3706023161
H	4.0113342200	-2.2504231300	5.2031918259
H	5.1696966874	-0.9800114404	4.8161454911
H	5.3565191524	-2.5935428841	4.1014240187
H	-0.1695431676	-3.3310790505	-0.9894240144
H	0.4489199613	-2.5669344613	-2.4725397920
H	0.5373238687	-1.7047698066	-0.9315150395
H	3.0679395255	-5.1598429653	-1.6611368075
H	2.0875028036	-4.4763856851	-2.9744766140
H	1.3019253944	-5.3317144071	-1.6484569509

102

$^{2}\mathbf{3}_{N_2}$ E_{tot}(r^{2}SCAN-3c) = -4972.487112 $\langle S^2 \rangle$ = 0.782

Ni	-1.9837710000	0.0000000000	0.0000000000
Ni	1.9837710000	0.0000000000	0.0000000000
N	-0.6751560000	1.2374428286	-0.5416635984
N	-3.1929100000	1.3818908267	-0.4963239923
N	-3.3388880000	-1.2588416971	0.4542325419
N	-0.5711920000	-1.1207356649	0.5096550699
N	0.5711990000	-1.1108098691	0.5309339089
N	0.6751590000	1.2103445457	-0.5997760538
N	3.1929140000	1.2684507877	-0.7395969411
N	3.3388980000	-1.1571399413	0.6723350271
C	-1.0987630000	2.4011033711	-1.0607689206
C	-2.5736910000	2.5897662276	-1.0662641492
C	-4.5088510000	1.3599529181	-0.3320019342
C	-4.6454630000	-1.0064831999	0.4644296192
C	-2.9183590000	-2.6056705062	0.6873425881
C	1.0987680000	2.3559901395	-1.1575145579
C	0.0000030000	3.1587536239	-1.4729510050
C	-5.1975480000	0.2400899322	0.1454016087
C	-5.3272570000	2.5825269847	-0.6745512661
C	-5.6217750000	-2.1184359302	0.7781183396
C	-2.4225520000	-2.9639648969	1.9544482768
C	-2.9635960000	-3.5321795413	-0.3711856247
C	2.5736960000	2.4814605779	-1.2985263647
C	-2.0194120000	-4.2846585071	2.1559021763
C	-2.3392070000	-1.9505608936	3.0819029630
C	-2.5530070000	-4.8419844476	-0.1211949748
C	-3.3817060000	-3.1079862550	-1.7685561950
C	4.5088500000	1.1284466162	-0.8284593359
C	4.6454690000	-1.0027622294	0.4724163650
C	2.9183790000	-2.2013964029	1.5542780735
C	-2.0933590000	-5.2202728989	1.1333254255
C	-1.0662470000	-2.1071159028	3.9189751108
C	-3.5850270000	-2.0098428127	3.9787888038
C	-2.1413740000	-2.7221914551	-2.5895575573
C	-4.2046640000	-4.1709070848	-2.5033763699

C	5.1975450000	0.0428908745	-0.2774813043
C	5.3272520000	2.1766818921	-1.5448675799
C	5.6218030000	-1.9578051195	1.1225614642
C	2.9636680000	-1.9860028674	2.9444258538
C	2.4225530000	-3.4023883830	1.0143336263
C	2.5531130000	-3.0193971920	3.7871619795
C	3.3818090000	-0.6428647124	3.5176111393
C	2.0194470000	-4.4055970204	1.8966125849
C	2.3392160000	-3.6147549580	-0.4866840486
C	2.0934460000	-4.2235974972	3.2706222709
C	2.1414840000	0.2340304067	3.7498866554
C	4.2048700000	-0.7631452418	4.8041263441
C	1.0662970000	-4.3567106429	-0.9047947429
C	3.5850800000	-4.3398705487	-1.0177209837
H	-2.8379650000	3.4875232521	-0.4852523843
H	-2.9292590000	2.7638959704	-2.0948279798
H	0.0000030000	4.1376281465	-1.9294087947
H	-6.2730470000	0.3297059992	0.2327280277
H	-5.0313530000	3.4399916243	-0.0589913068
H	-6.3880570000	2.3930212075	-0.5068172971
H	-5.1923610000	2.8742471032	-1.7223710719
H	-5.7864910000	-2.7339367547	-0.1149977993
H	-6.5849980000	-1.7034679284	1.0827212001
H	-5.2502810000	-2.7879324505	1.5568851844
H	2.8379530000	2.6134411586	-2.3597203844
H	2.9292770000	3.3813144475	-0.7707792476
H	-1.6333740000	-4.5837248546	3.1258054613
H	-2.3147690000	-0.9540400946	2.6236349289
H	-2.5819720000	-5.5760522119	-0.9212913303
H	-4.0017850000	-2.2088910272	-1.6817899344
H	-1.7758200000	-6.2443530903	1.3094752861
H	-1.0864270000	-3.0166767144	4.5303281380
H	-0.9670390000	-1.2584925119	4.6047523562
H	-0.1723010000	-2.1471395268	3.2897128516
H	-3.7069350000	-3.0125706802	4.4055567450
H	-4.4936170000	-1.7625619033	3.4220514180
H	-3.4937280000	-1.2959563042	4.8053595731
H	-1.5762070000	-1.9225580544	-2.0970777082
H	-2.4309600000	-2.3713549121	-3.5867485148
H	-1.4755410000	-3.5846515812	-2.7059943817
H	-3.5991390000	-5.0445680286	-2.7682554033
H	-4.6010450000	-3.7575432099	-3.4369401485
H	-5.0478410000	-4.5186492042	-1.8966761099
H	6.2730390000	0.0335703111	-0.4023086105
H	5.0313260000	2.2562763071	-2.5973941795
H	6.3880490000	1.9263665968	-1.5075305786
H	5.1923770000	3.1668870763	-1.0948381174
H	5.7867180000	-1.6691354970	2.1680759787
H	6.5849460000	-1.9245433129	0.6087205224
H	5.2502400000	-2.9846892956	1.1350428813
H	2.5821300000	-2.8782979773	4.8637773514
H	4.0018220000	-0.1314235042	2.7730270101
H	1.6333960000	-5.3408420773	1.5023256055
H	2.3147290000	-2.6231737213	-0.9555450081
H	1.7759210000	-5.0167676963	3.9419341801
H	1.5762130000	0.3707037544	2.8208262803
H	2.4310810000	1.2234626227	4.1220360307
H	1.4757410000	-0.2311388064	4.4855020984
H	3.5994230000	-1.1217979655	5.6437143974
H	4.6012620000	0.2177259179	5.0874935833
H	5.0480480000	-1.4514190074	4.6804807800
H	1.0865230000	-5.4096686156	-0.6009318919
H	0.9671010000	-4.3366414521	-1.9956897134
H	0.1723220000	-3.9004095067	-0.4696922175
H	4.4936370000	-3.7543920699	-0.8492670597
H	3.4938200000	-4.5142090685	-2.0958981457
H	3.7070150000	-5.3113176586	-0.5238807063

102

43N2 E_{tot}(r^{2}SCAN-3c) = -4972.462319 $\langle S^2 \rangle$ = 3.798

Ni	-2.0537380000	0.0000000000	0.0000000000
Ni	2.0537380000	0.0000000000	0.0000000000

N	-0.6739630000	1.2522000171	-0.5371404743
N	-3.2446490000	1.4465068656	-0.5435959220
N	-3.4734130000	-1.2386282833	0.3961412857
N	-0.5602280000	-1.1978786337	0.5476201275
N	0.5632340000	-1.1978930770	0.5441534289
N	0.6739970000	1.2183801371	-0.6101643433
N	3.2434870000	1.3431330371	-0.7638029368
N	3.4725040000	-1.0921956145	0.7088373522
C	-1.1003160000	2.4151372783	-1.0520588392
C	-2.5770280000	2.6468660411	-1.0554151018
C	-4.5646760000	1.4284249917	-0.4576927319
C	-4.7765930000	-0.9615751238	0.3341383708
C	-3.0677910000	-2.5792006425	0.6641510763
C	1.0998630000	2.3591275750	-1.1724299517
C	-0.0004060000	3.1677994819	-1.4771691579
C	-5.2806260000	0.2976443953	-0.0213492118
C	-5.3681750000	2.6475249388	-0.8455420978
C	-5.7890200000	-2.0500659985	0.6122173247
C	-2.5641500000	-2.9052951830	1.9395881016
C	-3.0613160000	-3.5195853538	-0.3844808140
C	2.5764340000	2.5080984338	-1.3517893346
C	-2.1086740000	-4.2059890395	2.1576762384
C	-2.5077910000	-1.8655954122	3.0452454781
C	-2.5954780000	-4.8072128195	-0.1178827722
C	-3.4545020000	-3.1209059397	-1.7966624363
C	4.5635010000	1.2624754509	-0.8093797615
C	4.7749120000	-0.8665224148	0.5348869376
C	3.0651770000	-2.1458437553	1.5792954428
C	-2.1321790000	-5.1545171974	1.1442901771
C	-1.3165180000	-2.0616231420	3.9879935275
C	-3.8212910000	-1.8184320061	3.8405910479
C	-2.2058470000	-2.6784506245	-2.5761467421
C	-4.2015670000	-4.2233854607	-2.5534417899
C	5.2786120000	0.2054152867	-0.2163406893
C	5.3670760000	2.3356384857	-1.5056700344
C	5.7909690000	-1.7632138184	1.2065607012
C	3.0496560000	-1.9231379051	2.9699879412
C	2.5693550000	-3.3446064657	1.0280864669
C	2.5829690000	-2.9422164379	3.8003263857
C	3.4320970000	-0.5736898065	3.5529781768
C	2.1118790000	-4.3340593166	1.8987331228
C	2.5293520000	-3.5557154475	-0.4751583562
C	2.1265600000	-4.1433747738	3.2738390722
C	2.1686110000	0.2794929142	3.7463279462
C	4.2192670000	-0.6809085724	4.8629234575
C	1.3194710000	-4.3792300374	-0.9285029058
C	3.8328900000	-4.1893318939	-0.9841224470
H	-2.8144010000	3.5299272350	-0.4391065793
H	-2.9136470000	2.8837582051	-2.0780135699
H	-0.0008280000	4.1467938587	-1.9358686282
H	-6.3595000000	0.3979988815	0.0011369256
H	-5.0811390000	3.5154426375	-0.2404130913
H	-6.4363220000	2.4733432333	-0.7088519233
H	-5.1966530000	2.9162729971	-1.8945420633
H	-5.9062410000	-2.6924126714	-0.2694572864
H	-6.7648900000	-1.6195400168	0.8468736534
H	-5.4716190000	-2.6993420044	1.4321561001
H	2.8088690000	2.6056277939	-2.4254518393
H	2.9168990000	3.4424646051	-0.8761299625
H	-1.7125710000	-4.4780978407	3.1311095038
H	-2.3891300000	-0.8863539436	2.5613245259
H	-2.5810790000	-5.5475498340	-0.9125389234
H	-4.1181940000	-2.2513304717	-1.7381817215
H	-1.7697180000	-6.1611323281	1.3331056537
H	-1.4486360000	-2.9345389346	4.6378722589
H	-1.2142990000	-1.1873526132	4.6400339461
H	-0.3793120000	-2.1914879954	3.4363644358
H	-4.0411860000	-2.7993586843	4.2784483998
H	-4.6639880000	-1.5284131742	3.2065136816
H	-3.7482500000	-1.0886852838	4.6550237597
H	-1.7050860000	-1.8427412894	-2.0732061517
H	-2.4743130000	-2.3534971458	-3.5878993580

H	-1.4896240000	-3.5043719778	-2.6559657113
H	-3.5476070000	-5.0683834786	-2.7949275298
H	-4.5860560000	-3.8319684424	-3.5013272568
H	-5.0469090000	-4.6082901777	-1.9726768776
H	6.3573790000	0.2494217003	-0.3105359674
H	5.0745380000	2.4238729636	-2.5585005577
H	6.4345930000	2.1152139107	-1.4645300914
H	5.2014770000	3.3156765822	-1.0428879539
H	5.9493420000	-1.4412750639	2.2436540779
H	6.7522230000	-1.7107763129	0.6904081820
H	5.4552750000	-2.8020964767	1.2465413609
H	2.5614260000	-2.7904276954	4.8758840709
H	4.0646800000	-0.0519441206	2.8269475013
H	1.7223060000	-5.2634209725	1.4946907017
H	2.4495020000	-2.5636740860	-0.9398027097
H	1.7633500000	-4.9248074748	3.9354892736
H	1.6357450000	0.4129551797	2.7973649085
H	2.4243340000	1.2717742377	4.1352540891
H	1.4843830000	-0.2044250878	4.4527795446
H	3.5969740000	-1.0530594783	5.6840018809
H	4.5894810000	0.3062746084	5.1597038272
H	5.0777780000	-1.3535964023	4.7613797657
H	1.4160400000	-5.4346809043	-0.6488707385
H	1.2340320000	-4.3415715965	-2.0199887774
H	0.3868060000	-4.0013735047	-0.4966419701
H	4.6954550000	-3.5464378928	-0.7859510782
H	3.7803020000	-4.3566173996	-2.0660188010
H	4.0063780000	-5.1559406063	-0.4963725897

100

BSS 2^- E_{tot}(r^2SCAN-3c) = -4863.010142 $\langle S^2 \rangle$ = 1.022

Ni	-2.0840951789	0.0000000000	-0.0000000000
Ni	2.0840951789	-0.0000000000	0.0000000000
N	-0.6757684480	1.2559461119	-0.2430659699
N	-3.2518652622	1.5093290010	-0.1426898869
N	-3.3870206692	-1.3078411815	0.2772444883
N	0.6753880421	1.2435559346	-0.3009520974
N	3.2521861903	1.4361656993	-0.4933106286
N	3.3897223942	-1.3014235354	0.2931079274
C	-1.1008571670	2.5138830172	-0.4883411004
C	-2.5784660800	2.7674174602	-0.4497431153
C	-4.5525950234	1.4630088979	0.0684354652
C	-4.6855743857	-1.0358304225	0.4512352386
C	-2.9494117228	-2.6498685191	0.1681722118
C	1.0997970387	2.4906443991	-0.5971888971
C	-0.0006754512	3.3428919050	-0.7245134727
C	-5.2261349161	0.2563265032	0.3729970687
C	-5.3806517850	2.7282509776	0.0000000000
C	-5.6306169700	-2.1759623482	0.7635716772
C	-2.2494086671	-3.2504629842	1.2372843784
C	-3.1635062438	-3.3612429686	-1.0376103340
C	2.5773423291	2.7071817910	-0.7364270438
C	-1.7819949843	-4.5554013976	1.0847329455
C	-1.9759617050	-2.4692373799	2.5072450019
C	-2.6872222714	-4.6667444828	-1.1395643394
C	-3.8067824381	-2.6689057649	-2.2264763682
C	4.5534231555	1.3087885059	-0.6643155548
C	4.6885651844	-1.1235715374	0.0239807838
C	2.9554980254	-2.4801702437	0.9472248065
C	-2.0035081540	-5.2678339332	-0.0874449928
C	-0.5895521864	-1.8120919163	2.4444786556
C	-2.1027033475	-3.3206638518	3.7751929527
C	-2.7279524100	-1.9395127038	-3.0415164181
C	-4.6193267934	-3.6090844166	-3.1208573307
C	5.2282295510	0.0854225891	-0.4399418192
C	5.3804544032	2.4900696748	-1.1251759841
C	5.6358295066	-2.2884861703	0.2152584575
C	3.1668756859	-2.6272475729	2.3397873735
C	2.2644042666	-3.4740867573	0.2206255951
C	2.6948232420	-3.7756972675	2.9721720274
C	3.8046986276	-1.5027842266	3.1366655620
C	1.8007510031	-4.6010564172	0.8986730503

C	1.9977691456	-3.2917266372	-1.2603496908
C	2.0182294429	-4.7620873993	2.2615743225
C	2.7205501898	-0.5102643630	3.5832866399
C	4.6245994798	-1.9866935910	4.3357923501
C	0.6121943028	-2.6686713724	-1.4822518521
C	2.1291032300	-4.5927963570	-2.0594414358
H	-2.7993548520	3.5461444558	0.3019436491
H	-2.9147659405	3.1739256980	-1.4208133447
H	-0.0007208751	4.4001183937	-0.9529242714
H	-6.2910320940	0.3388325492	0.5623717052
H	-5.0593144372	3.4530109469	0.7590701253
H	-6.4397529356	2.5135747146	0.1600187524
H	-5.2725719713	3.2198548526	-0.9751166457
H	-5.7290415280	-2.8643228519	-0.0850222425
H	-6.6235867951	-1.7972010636	1.0167495177
H	-5.2533730270	-2.7718748607	1.6035033977
H	2.7985390627	3.1041437078	-1.7433253238
H	2.9119499016	3.4806178485	-0.0214196396
H	-1.2286356897	-5.0214021612	1.8952368566
H	-2.7219548069	-1.6666696592	2.5587048746
H	-2.8394285794	-5.2233624529	-2.0604694223
H	-4.4887780862	-1.9016479042	-1.8433412209
H	-1.6348594671	-6.2857376684	-0.1861945336
H	0.1985078479	-2.5686084157	2.3586552924
H	-0.4043604637	-1.2163049362	3.3457588771
H	-0.5080616354	-1.1325580145	1.5834243678
H	-1.2894980520	-4.0517050478	3.8544550792
H	-3.0544501369	-3.8636866155	3.8010055487
H	-2.0461835832	-2.6783330509	4.6614395191
H	-2.1756429662	-1.2371370825	-2.4033487792
H	-3.1796944664	-1.3719988089	-3.8643107249
H	-2.0160868544	-2.6602750746	-3.4616283859
H	-3.9775897508	-4.3138478091	-3.6623194054
H	-5.1659949047	-3.0276705700	-3.8720485808
H	-5.3454752121	-4.1895984587	-2.5401160533
H	6.2935494678	0.0834714503	-0.6443057277
H	5.0597703301	2.8348503417	-2.1165987123
H	6.4399883226	2.2298736002	-1.1806169147
H	5.2702799196	3.3411586475	-0.4413363303
H	5.7335720713	-2.5611937536	1.2733915256
H	6.6287581640	-2.0471743827	-0.1712600701
H	5.2612724492	-3.1806658864	-0.3010258368
H	2.8447740714	-3.9000574312	4.0413435383
H	4.4808369649	-0.9565481838	2.4696311465
H	1.2543126813	-5.3646330812	0.3522020163
H	2.7449700099	-2.5824928911	-1.6372005944
H	1.6528402046	-5.6495845712	2.7720677139
H	2.1603558973	-0.1387250514	2.7151439474
H	3.1683507409	0.3500098283	4.0956478953
H	2.0163549611	-0.9986153447	4.2676561767
H	3.9885456596	-2.4115242889	5.1210529550
H	5.1668522518	-1.1441981043	4.7800531255
H	5.3551684961	-2.7493317694	4.0422488452
H	-0.1773364043	-3.3218360051	-1.0938727294
H	0.4316612494	-2.4999491575	-2.5502525620
H	0.5280357518	-1.6933958686	-0.9805340612
H	3.0800748540	-5.0970797068	-1.8527634464
H	2.0777367563	-4.3765200729	-3.1326517441
H	1.3150920027	-5.2910866617	-1.8319269472

100

^{3}2^{-} E_{tot}(r^{2}SCAN-3c) = -4863.004843 $\langle S^2 \rangle$ = 2.030

Ni	-2.1058624208	-0.0000000000	0.0000000000
Ni	2.1058624208	0.0000000000	-0.0000000000
N	-0.6784287979	1.2467165943	-0.2071370147
N	-3.2642873785	1.5175345099	-0.1361322259
N	-3.4273728827	-1.2970888596	0.2738776086
N	0.6784646451	1.2441205291	-0.2223013440
N	3.2642994175	1.4757430545	-0.3796584896
N	3.4278544787	-1.3133591150	0.1774632701
C	-1.1004485973	2.5134140320	-0.4134379710
C	-2.5766235803	2.7719444379	-0.4191159611

C	-4.5660545964	1.4829279787	0.0680432108
C	-4.7197623204	-1.0115353276	0.4680896338
C	-2.9926618094	-2.6409301556	0.2343626130
C	1.1004668295	2.5069357087	-0.4513160820
C	-0.0000073080	3.3570547702	-0.5781483696
C	-5.2505906859	0.2840309557	0.3741348606
C	-5.3816464640	2.7563456966	0.0000000000
C	-5.6824168681	-2.1273097950	0.8239302874
C	-2.1536664257	-3.1272299321	1.2656845971
C	-3.3354159226	-3.4696303519	-0.8610199527
C	2.5766019094	2.7525126816	-0.5326224705
C	-1.7363967089	-4.4560856418	1.2174771436
C	-1.7317924449	-2.2065522093	2.3938250373
C	-2.9004812374	-4.7941601201	-0.8593868142
C	-4.0588767759	-2.8909270645	-2.0655857803
C	4.5661166013	1.3749825989	-0.5601880613
C	4.7202615154	-1.1091807151	-0.1009243609
C	2.9934872509	-2.5658297739	0.6663438137
C	-2.1211371708	-5.2948014063	0.1773649736
C	-0.4099746079	-2.6020016741	3.0471118189
C	-2.8442576124	-2.0635085606	3.4416661679
C	-3.0324775580	-2.3237316872	-3.0586999791
C	-4.9926929653	-3.8858887151	-2.7602877306
C	5.2508532347	0.1429420587	-0.4469212915
C	5.3815986614	2.5978346890	-0.9221679160
C	5.6832166414	-2.2793994463	-0.0614273853
C	3.3363289350	-2.9781371402	1.9765077532
C	2.1545999076	-3.3706100821	-0.1415391436
C	2.9015476003	-4.2262217831	2.4201580788
C	4.0596427986	-2.0281602494	2.9165349299
C	1.7374947114	-4.6059898733	0.3505019902
C	1.7326770948	-2.8827055132	-1.5134930658
C	2.1222887753	-5.0462834148	1.6120119414
C	3.0331023063	-1.1604952988	3.6614094235
C	4.9937296705	-2.7316355262	3.9050569230
C	0.4110497540	-3.4750438162	-1.9959809550
C	2.8452532584	-3.0998260951	-2.5484043573
H	-2.8163105391	3.5521896831	0.3250029368
H	-2.8803660411	3.1805652712	-1.4003263184
H	-0.0000271845	4.4229634103	-0.7616240684
H	-6.3135742500	0.3773191740	0.5700628184
H	-5.0710175609	3.4685133852	0.7753312187
H	-6.4455510933	2.5485204548	0.1349409442
H	-5.2479647729	3.2587510573	-0.9661487022
H	-5.9766108714	-2.7106608105	-0.0576737642
H	-6.5903760499	-1.7150016112	1.2714456298
H	-5.2281482949	-2.8331981862	1.5271930066
H	2.8161761152	3.2387936869	-1.4949563912
H	2.8804488291	3.4659008663	0.2552580684
H	-1.0822334661	-4.8390968561	1.9947680025
H	-1.5894493912	-1.2053457924	1.9454264731
H	-3.1583030131	-5.4450084231	-1.6908773663
H	-4.6637340461	-2.0429960530	-1.7295753682
H	-1.7872708621	-6.3291815554	0.1591978822
H	-0.4961372992	-3.5227823658	3.6382793387
H	-0.0855269272	-1.8059466683	3.7253541390
H	0.3772823328	-2.7517569971	2.3018844454
H	-3.0877043907	-3.0399747657	3.8799129356
H	-3.7507893924	-1.6432800929	2.9965990030
H	-2.5233730693	-1.3944940771	4.2493516092
H	-2.3977846345	-1.5775915676	-2.5665400086
H	-3.5385932831	-1.8420536574	-3.9044349934
H	-2.3910481121	-3.1242590290	-3.4460561376
H	-4.4361702125	-4.6797795300	-3.2720090088
H	-5.5902101687	-3.3693745382	-3.5201312849
H	-5.6774596530	-4.3607121312	-2.0482304048
H	6.3138547224	0.1653642168	-0.6626816273
H	5.0706818070	3.0097697435	-1.8909007035
H	6.4454903345	2.3568664796	-0.9801354931
H	5.2481166505	3.3943646610	-0.1795674866
H	5.9776556024	-2.5325531341	0.9648763503
H	6.5910257994	-2.0411213077	-0.6215797616

H	5.2291533520	-3.1806613884	-0.4865884636
H	3.1594282783	-4.5596729060	3.4220368451
H	4.6642625352	-1.3422623403	2.3151030538
H	1.0834331054	-5.2280840222	-0.2528246420
H	1.5900185465	-1.7890728771	-1.4276342622
H	1.7885200261	-6.0144312828	1.9767381184
H	2.3983776450	-0.6231069474	2.9471756848
H	3.5390786932	-0.4226465679	4.2962353916
H	2.3917078805	-1.7844697334	4.2951184455
H	4.4374347990	-3.3075117564	4.6538845393
H	5.5911926986	-1.9897134383	4.4470592926
H	5.6785496216	-3.4180054425	3.3938739701
H	0.4974789947	-4.5409515955	-2.2431927942
H	0.0866365741	-2.9534375075	-2.9024411813
H	-0.3763311838	-3.3657090530	-1.2438978696
H	3.7516612569	-2.5543041489	-2.2703752473
H	2.5243534083	-2.7411690070	-3.5339457852
H	3.0889212295	-4.1667247933	-2.6330440365

100

^{5}2$^-$ E_{tot}(r^{2}SCAN-3c) = -4862.949064 $\langle S^2 \rangle$ = 6.032

Ni	-2.0996579709	-0.0000000000	0.0000000000
Ni	2.0996579709	0.0000000000	-0.0000000000
N	-0.6837374625	1.2677369872	0.1374219049
N	-3.2696904696	1.5327532097	0.0611812516
N	-3.3694948208	-1.3108527083	-0.3039083906
N	0.6800566746	1.2666792240	0.1102721547
N	3.2526383162	1.5267241627	-0.1117491290
N	3.4138840682	-1.3166879280	-0.1776283402
C	-1.1123699119	2.5534067534	0.1532313961
C	-2.5913515804	2.8062550174	0.1605575975
C	-4.6245878299	1.4661907960	-0.0533214628
C	-4.7495906514	-1.0513595050	-0.3487092951
C	-2.8995437062	-2.5128529343	-0.8583658121
C	1.0936922232	2.5457856080	0.1049543016
C	-0.0130323045	3.4050786067	0.1361713608
C	-5.2788229814	0.2356423022	-0.2204940743
C	-5.4404556156	2.7328506501	0.0000000000
C	-5.6685647800	-2.2386429889	-0.4175100503
C	-2.1401237755	-3.4076892860	-0.0557974170
C	-3.1482801264	-2.8378052404	-2.2269847507
C	2.5672962164	2.8051095225	0.0332132558
C	-1.6884008244	-4.6019433474	-0.6083245758
C	-1.8382928822	-3.0599416535	1.3888544974
C	-2.6878095750	-4.0518052036	-2.7257700758
C	-3.7583482717	-1.7978596873	-3.1482410578
C	4.5475665326	1.4679511283	-0.3554213570
C	4.6999552743	-1.0557953382	-0.4340777853
C	2.9928055625	-2.6525393619	0.0506421082
C	-1.9663342081	-4.9413698608	-1.9312014351
C	-0.4737572067	-2.3670139370	1.5110911991
C	-1.8942094687	-4.2656174889	2.3329885622
C	-2.6761622631	-0.7946279480	-3.5811767674
C	-4.4621924744	-2.3813671024	-4.3751510102
C	5.2282197569	0.2408128025	-0.5226449567
C	5.3554551130	2.7407400973	-0.4843006277
C	5.6449370726	-2.2158184955	-0.6563171447
C	3.2069212518	-3.2414833540	1.3180344493
C	2.3098944324	-3.3559386183	-0.9639275275
C	2.7305710894	-4.5315984621	1.5448914115
C	3.8605789221	-2.4437779905	2.4324038140
C	1.8421930921	-4.6404405243	-0.6870554158
C	2.0572779794	-2.7089965714	-2.3121788170
C	2.0524125766	-5.2310385274	0.5525482294
C	2.7891992643	-1.6712189896	3.2172016071
C	4.7127634309	-3.2970563038	3.3765214863
C	0.6769778116	-2.0361479097	-2.3419988166
C	2.1898219111	-3.6922416759	-3.4804831373
H	-2.8563518255	3.3588909722	1.0848144034
H	-2.8364188907	3.4922026758	-0.6742072408
H	-0.0134514902	4.4864705749	0.1329664674
H	-6.3651515368	0.2884510517	-0.2468277677

H	-5.2603099824	3.3055360562	0.9219465685
H	-6.5081424307	2.4998916032	-0.0437519360
H	-5.2176730637	3.4121991754	-0.8369573121
H	-5.6589358278	-2.7584575367	-1.3904943526
H	-6.6992448855	-1.9324590034	-0.2135650242
H	-5.3783837947	-2.9985373907	0.3240526300
H	2.7740969606	3.4858459022	-0.8108416127
H	2.8974520009	3.3357740780	0.9445796996
H	-1.1140084834	-5.2885133139	0.0074798285
H	-2.6096441161	-2.3463176281	1.7057975618
H	-2.8703330489	-4.3042456134	-3.7671941542
H	-4.4999403377	-1.2268252337	-2.5745544683
H	-1.6168294041	-5.8851654219	-2.3417717773
H	0.3355224815	-3.0505199295	1.2319465854
H	-0.2986500549	-2.0146205609	2.5344051272
H	-0.3998841765	-1.4918445608	0.8481595689
H	-1.0637093494	-4.9595190640	2.1568162288
H	-2.8331069789	-4.8178392187	2.2162556186
H	-1.8174966288	-3.9281230236	3.3733566854
H	-2.1829820916	-0.3552518499	-2.7066702065
H	-3.1151849311	0.0221396891	-4.1674253721
H	-1.9140790808	-1.2942485892	-4.1916395197
H	-3.7520255346	-2.8280688357	-5.0818145903
H	-4.9942386004	-1.5867225486	-4.9106975754
H	-5.1902915767	-3.1506850241	-4.0934406784
H	6.2859204765	0.3088192944	-0.7520221062
H	5.0070070229	3.3435894203	-1.3324885359
H	6.4142179054	2.5197918316	-0.6358022102
H	5.2583906630	3.3650128303	0.4126798374
H	5.7486870056	-2.8298852980	0.2465030989
H	6.6350901013	-1.8595819194	-0.9489448703
H	5.2617862554	-2.8808231356	-1.4399321239
H	2.8788076727	-4.9959808818	2.5160740558
H	4.5185275855	-1.6980922490	1.9723123698
H	1.2929700003	-5.1838588258	-1.4502780242
H	2.8130278312	-1.9241711152	-2.4413460647
H	1.6788671739	-6.2326595974	0.7492153585
H	2.2182597551	-1.0162351956	2.5466992674
H	3.2502745057	-1.0490253361	3.9938687122
H	2.0899506024	-2.3678345564	3.6951486057
H	4.0984408062	-3.9669523881	3.9890810130
H	5.2688071837	-2.6495791534	4.0639288039
H	5.4327019394	-3.9108776922	2.8232891152
H	-0.1219105356	-2.7688643316	-2.1821462120
H	0.5108883575	-1.5445402414	-3.3073662354
H	0.5951502926	-1.2633224102	-1.5643215265
H	3.1326987908	-4.2490212797	-3.4326140883
H	2.1574477260	-3.1464886469	-4.4301587045
H	1.3650887649	-4.4140463810	-3.4954287234

100

^{1}2^{-} E_{tot}(r^{2}SCAN-3c) = -4862.975024 $\langle S^2 \rangle$ = 0.000

Ni	-1.9962277346	0.0000000000	0.0000000000
Ni	1.9962277346	-0.0000000000	-0.0000000000
N	-0.6676929237	1.2835589470	-0.2587976349
N	-3.2119861960	1.4781768934	-0.1619692286
N	-3.2985896903	-1.3037661339	0.2749241445
N	0.6677012271	1.2700001629	-0.3187198140
N	3.2120096280	1.4041057233	-0.4895567595
N	3.2986120966	-1.2951356606	0.3131260886
C	-1.0986793675	2.5361201446	-0.5164350454
C	-2.5760234031	2.7546897494	-0.4782751911
C	-4.5119064169	1.4211099219	0.0603061370
C	-4.6017274840	-1.0631638524	0.4497198157
C	-2.8534871170	-2.6463253452	0.2264711693
C	1.0987119747	2.5116550764	-0.6246189822
C	0.0000262996	3.3643685067	-0.7604124993
C	-5.1715510716	0.2148779861	0.3697206163
C	-5.3508424065	2.6795400883	0.0000000000
C	-5.5438578220	-2.2156376347	0.7351138175
C	-2.1417141821	-3.1795030206	1.3226420759
C	-3.1429772906	-3.4450616126	-0.9072029729

C	2.5760632386	2.6925562673	-0.7530808349
C	-1.7694578028	-4.5220100079	1.2873007139
C	-1.8292370638	-2.3022252304	2.5177838854
C	-2.7641575974	-4.7855850399	-0.8885607453
C	-3.7807641293	-2.8197255712	-2.1369308419
C	4.5119502931	1.2569608162	-0.6654994389
C	4.6017627779	-1.1531255860	0.0519642593
C	2.8534341565	-2.4859087655	0.9350284670
C	-2.0961092749	-5.3307473225	0.2037383905
C	-0.4572792755	-2.5832171120	3.1226244988
C	-2.9328291517	-2.4005036768	3.5804967177
C	-2.7014337617	-2.1829507308	-3.0260314984
C	-4.6393636443	-3.7939575610	-2.9477381286
C	5.1715882907	0.0349526705	-0.4258096196
C	5.3509501857	2.4189691302	-1.1522440133
C	5.5439240264	-2.3161699361	0.2905421793
C	3.1429478284	-2.7186233375	2.3021609530
C	2.1415603542	-3.4391179057	0.1753347250
C	2.7640356948	-3.9364719718	2.8626204130
C	3.7808487596	-1.6247368186	3.1427210406
C	1.7692022538	-4.6355072530	0.7853808754
C	1.8291195890	-3.1619831695	-1.2810938551
C	2.0958720582	-4.8988184716	2.1115870191
C	2.7016162435	-0.6670622638	3.6709793444
C	4.6394383994	-2.1549266715	4.2940065291
C	0.4572286054	-3.6761231451	-1.7060744775
C	2.9327999078	-3.7081779841	-2.1978747876
H	-2.8170853903	3.5296531686	0.2700612798
H	-2.9269161624	3.1417720721	-1.4514916839
H	0.0000354609	4.4190242557	-0.9986741801
H	-6.2372974587	0.2796486052	0.5595030014
H	-5.0373387124	3.4016695588	0.7646165610
H	-6.4069419571	2.4506997811	0.1601790497
H	-5.2503479578	3.1793051153	-0.9715247132
H	-5.8051803894	-2.7572092366	-0.1828705735
H	-6.4702260759	-1.8425351632	1.1796593570
H	-5.0936062937	-2.9494796465	1.4102714590
H	2.8171371418	3.0702760362	-1.7619908251
H	2.9269589686	3.4606092451	-0.0409912114
H	-1.2107831209	-4.9423216504	2.1188127276
H	-1.8146868110	-1.2639207294	2.1514331926
H	-2.9829892290	-5.4174474639	-1.7452591821
H	-4.4300879338	-2.0029148674	-1.8040222306
H	-1.8038239226	-6.3777256041	0.1967960714
H	-0.3954109873	-3.5750654760	3.5857561861
H	-0.2414629645	-1.8428893485	3.9006261486
H	0.3275928491	-2.5152238338	2.3638419806
H	-3.0328377118	-3.4325088275	3.9409922056
H	-3.9006136606	-2.0796360746	3.1823462577
H	-2.6929599718	-1.7601686122	4.4382565593
H	-2.1006467332	-1.4666066128	-2.4520056364
H	-3.1613484261	-1.6487508220	-3.8664859104
H	-2.0336693859	-2.9542646131	-3.4263758883
H	-4.0299197793	-4.5565665973	-3.4467423647
H	-5.1814439002	-3.2505411122	-3.7300577351
H	-5.3716805440	-4.3084485440	-2.3147655911
H	6.2373502726	0.0117790449	-0.6249143295
H	5.0374851362	2.7421265572	-2.1530945684
H	6.4070399591	2.1434447319	-1.1984145695
H	5.2504782917	3.2879559829	-0.4900558780
H	5.8055227683	-2.4095330701	1.3522100916
H	6.4701577532	-2.1709297459	-0.2715834377
H	5.0935756074	-3.2692361484	-0.0026014297
H	2.9828802747	-4.1378494736	3.9079075947
H	4.4302054799	-1.0309575522	2.4905012389
H	1.2104395239	-5.3728796043	0.2159429730
H	1.8145076524	-2.0671105836	-1.3975229577
H	1.8035281437	-5.8407306299	2.5687308817
H	2.1008714504	-0.2676508886	2.8444363697
H	3.1616205939	0.1769173930	4.1995126282
H	2.0338009451	-1.1907618013	4.3644297878
H	4.0299647943	-2.6282002543	5.0728221249

H	5.1816562139	-1.3276419262	4.7659974327
H	5.3716326690	-2.8919622177	3.9442758136
H	0.3954546363	-4.7707420147	-1.6970299513
H	0.2414242853	-3.3429395562	-2.7270364488
H	-0.3277116905	-3.2881331516	-1.0505370343
H	3.9005473820	-3.2471012072	-1.9766083326
H	2.6929836197	-3.4995640655	-3.2477730398
H	3.0328519156	-4.7948371227	-2.0788963490

102

$^{3}(\mathbf{2}^{\mathbf{N_2}})^{-}$ $E_{\mathrm{tot}}(\mathrm{r^2SCAN\text{-}3c}) = -4972.539670$ $\langle S^2 \rangle = 2.030$

Ni	-2.0304082432	0.0000000000	0.0000000000
Ni	2.0304082432	-0.0000000000	-0.0000000000
N	-0.6681096220	1.4032469260	0.0353760459
N	-3.2355860461	1.5543989204	0.1285829524
N	-3.4769025996	-1.3091328903	-0.1331708620
N	-0.5675542891	-1.2508736913	0.0049071331
N	0.5675544666	-1.2508739233	-0.0049704648
N	0.6681124083	1.4032465776	-0.0353804297
N	3.2355887302	1.5543977292	-0.1285632510
N	3.4769002526	-1.3091302216	0.1332345285
C	-1.1007107591	2.6725372564	0.0604192516
C	-2.5826376233	2.8671757664	0.1543735322
C	-4.5512536747	1.4895778086	0.2281969721
C	-4.7728164321	-1.0167473903	-0.0523421757
C	-3.0831922841	-2.6269108962	-0.4707342881
C	1.1007139857	2.6725374438	-0.0604174233
C	0.0000016843	3.5389813815	0.0000000000
C	-5.2716038278	0.2785099976	0.1627648616
C	-5.3646206196	2.7544754257	0.4163499740
C	-5.8008540915	-2.1153263336	-0.2413072870
C	-2.5429230743	-3.4692617980	0.5254667491
C	-3.1099262013	-3.0421545514	-1.8190397364
C	2.5826412192	2.8671753076	-0.1543693604
C	-2.0961127728	-4.7387449030	0.1593233960
C	-2.4445627068	-2.9948828584	1.9639571565
C	-2.6545394553	-4.3209300191	-2.1390564311
C	-3.5183090938	-2.0751038632	-2.9162858078
C	4.5512601756	1.4895766614	-0.2281316104
C	4.7728156581	-1.0167428750	0.0524618836
C	3.0831817742	-2.6269038394	0.4708025128
C	-2.1604316158	-5.1727867635	-1.1586383439
C	-1.2055308879	-3.5321631348	2.6856695431
C	-3.7205474758	-3.3297760047	2.7505925167
C	-2.2681359105	-1.3837165906	-3.4823475370
C	-4.3301531335	-2.7335935407	-4.0363618789
C	5.2716098534	0.2785107680	-0.1626512425
C	5.3646346111	2.7544718214	-0.4162702349
C	5.8008443775	-2.1153156790	0.2415076333
C	3.1098568747	-3.0421187949	1.8191163969
C	2.5429767610	-3.4692835607	-0.5254102551
C	2.6544506459	-4.3208847002	2.1391434039
C	3.5182100258	-2.0750445240	2.9163517927
C	2.0961447359	-4.7387564846	-0.1592566199
C	2.4447395115	-2.9949596244	-1.9639273800
C	2.1603882933	-5.1727638489	1.1587209593
C	2.2680110820	-1.3837026490	3.4824101279
C	4.3300829657	-2.7334934132	4.0364306434
C	1.2057631647	-3.5322589435	-2.6857229901
C	3.7207872169	-3.3298951010	-2.7504417625
H	-2.8237080053	3.4206057078	1.0792843845
H	-2.9294551774	3.5013149143	-0.6806655249
H	0.0000010548	4.6210472909	0.0000005872
H	-6.3494553808	0.3607472079	0.2505978110
H	-5.0693244772	3.2792341436	1.3333243797
H	-6.4312763456	2.5288551866	0.4791561242
H	-5.2096080911	3.4540906506	-0.4143349501
H	-5.9331776888	-2.3374162522	-1.3082038260
H	-6.7704172232	-1.8113305638	0.1621715680
H	-5.4875704742	-3.0494493944	0.2341941658
H	2.8237130807	3.4205950539	-1.0792860549
H	2.9294594630	3.5013214179	0.6806634650

H	-1.6678379438	-5.3924854424	0.9138186538
H	-2.3612202722	-1.9007776499	1.9296217202
H	-2.6649652761	-4.6507842812	-3.1749294574
H	-4.1393908971	-1.2926784692	-2.4672504222
H	-1.7979971439	-6.1620486900	-1.4259475426
H	-1.2842969286	-4.6060733699	2.8967124333
H	-1.0862775635	-3.0202008404	3.6474892855
H	-0.2972203951	-3.3689541777	2.0966947725
H	-3.9030071700	-4.4119782917	2.7498008924
H	-4.5937683056	-2.8327493334	2.3172952988
H	-3.6270832310	-2.9984458226	3.7921537135
H	-1.7200946218	-0.8660648623	-2.6862811105
H	-2.5433509486	-0.6451228480	-4.2452658040
H	-1.5948746098	-2.1204614834	-3.9368701900
H	-3.7211641479	-3.4268787856	-4.6281208265
H	-4.7110540350	-1.9692767841	-4.7235555268
H	-5.1833974453	-3.2939613272	-3.6371747529
H	6.3494646462	0.3607482659	-0.2504420066
H	5.0693732550	3.2792215185	-1.3332610974
H	6.4312921237	2.5288490480	-0.4790351988
H	5.2095933779	3.4540970031	0.4144009821
H	5.9331290578	-2.3373606741	1.3084189552
H	6.7704224565	-1.8113366817	-0.1619480867
H	5.4875752507	-3.0494564842	-0.2339687527
H	2.6648305497	-4.6507130949	3.1750252642
H	4.1392614083	-1.2926012708	2.4673029660
H	1.6679199448	-5.3925211237	-0.9137597089
H	2.3614060233	-1.9008526390	-1.9296398804
H	1.7979464466	-6.1620221532	1.4260339593
H	1.7199078881	-0.8661395538	2.6863292593
H	2.5431999409	-0.6450422394	4.2452730499
H	1.5948147818	-2.1204639626	3.9370014208
H	3.7211226943	-3.4267961386	4.6281986862
H	4.7109606677	-1.9691579649	4.7236168212
H	5.1833458037	-3.2938333358	3.6372444557
H	1.2845519597	-4.6061737181	-2.8967358153
H	1.0865794767	-3.0203198911	-3.6475638380
H	0.2974077813	-3.3690425061	-2.0968205808
H	4.5939781436	-2.8328668471	-2.3170855333
H	3.6274176082	-2.9985989277	-3.7920223982
H	3.9032330416	-4.4120997958	-2.7495974329

102

BSS(2^{N_2})$^-$ E_{tot}(r^{2}SCAN-3c) = -4972.543627 $\langle S^2 \rangle$ = 0.836

Ni	-1.9967544755	-0.0000000000	0.0000000000
Ni	1.9967544755	0.0000000000	-0.0000000000
N	-0.6702867713	1.3847507185	0.0352323144
N	-3.2253868907	1.5340993220	0.1369071878
N	-3.4312427584	-1.3183443068	-0.1377845369
N	-0.5707383016	-1.2921165276	-0.0057107887
N	0.5754936819	-1.2933342875	0.0180474226
N	0.6721170566	1.3840274076	-0.0396548366
N	3.2267226355	1.5301613786	-0.1361783385
N	3.4279408095	-1.3210824311	0.1333673912
C	-1.0989857409	2.6567342480	0.0624825811
C	-2.5789698553	2.8505058207	0.1590692042
C	-4.5385607745	1.4624600443	0.2510989483
C	-4.7301767693	-1.0401980576	-0.0420406631
C	-3.0444231507	-2.6361211135	-0.4895666781
C	1.1024871514	2.6558054107	-0.0641870295
C	0.0023015569	3.5207575214	0.0000000000
C	-5.2463438214	0.2455429664	0.1871960322
C	-5.3582200524	2.7205598409	0.4545932278
C	-5.7483970748	-2.1484199098	-0.2307554566
C	-2.5080087995	-3.4914925841	0.4959033358
C	-3.0876022880	-3.0392173810	-1.8402543440
C	2.5826293665	2.8478371624	-0.1588721170
C	-2.0860577402	-4.7664306002	0.1177817498
C	-2.3947634063	-3.0300815129	1.9373666878
C	-2.6578766349	-4.3237182124	-2.1729454848
C	-3.4918309839	-2.0572705729	-2.9258595156
C	4.5399821859	1.4564776862	-0.2509295241

C	4.7274444498	-1.0466503724	0.0355307685
C	3.0395167678	-2.6412492680	0.4777928280
C	-2.1711580064	-5.1905084884	-1.2018074071
C	-1.1499564763	-3.5776371122	2.6404919922
C	-3.6636162657	-3.3683106979	2.7342961258
C	-2.2382429234	-1.3672504106	-3.4861750907
C	-4.3097150598	-2.6967702106	-4.0524988063
C	5.2461497702	0.2386193261	-0.1897137376
C	5.3614140064	2.7137005966	-0.4529649521
C	5.7427815387	-2.1587987412	0.2166247649
C	3.0838813523	-3.0525498936	1.8257211777
C	2.5007823731	-3.4899549960	-0.5120754735
C	2.6522636317	-4.3383566128	2.1513365282
C	3.4912719734	-2.0787382888	2.9173927771
C	2.0773352133	-4.7665218230	-0.1411504511
C	2.3851282483	-3.0205569570	-1.9507184428
C	2.1629367085	-5.1984652792	1.1757661940
C	2.2393596102	-1.3923824473	3.4858594304
C	4.3112721031	-2.7273737120	4.0373344867
C	1.1353313766	-3.5587203259	-2.6524000819
C	3.6496984843	-3.3614419325	-2.7533301923
H	-2.8201704499	3.4056333171	1.0829880938
H	-2.9297619925	3.4813246485	-0.6770064956
H	0.0030916227	4.6023940797	0.0012253852
H	-6.3240974388	0.3130270675	0.2864221755
H	-5.0603855251	3.2397026027	1.3739723833
H	-6.4228122485	2.4870251339	0.5228332975
H	-5.2135349820	3.4278246034	-0.3714707788
H	-5.8841579694	-2.3664652470	-1.2979582941
H	-6.7183168945	-1.8543934041	0.1791941926
H	-5.4247841010	-3.0820785688	0.2382766508
H	2.8263809041	3.4024580701	-1.0823886263
H	2.9336627861	3.4776364513	0.6778283352
H	-1.6621402385	-5.4320184047	0.8643508613
H	-2.3078200353	-1.9362570545	1.9115782648
H	-2.6835098565	-4.6470679266	-3.2105211346
H	-4.1073437506	-1.2762622763	-2.4665629958
H	-1.8295107522	-6.1845770000	-1.4787972922
H	-1.2271148432	-4.6542885430	2.8379363188
H	-1.0218840990	-3.0786070101	3.6080371991
H	-0.2477620451	-3.4057343935	2.0451714419
H	-3.8511053889	-4.4496499703	2.7242935039
H	-4.5398497052	-2.8627579671	2.3171400583
H	-3.5564385253	-3.0487941041	3.7782364210
H	-1.6847749402	-0.8634465288	-2.6850512624
H	-2.5107731938	-0.6178074149	-4.2394753508
H	-1.5713215277	-2.1029955676	-3.9513496475
H	-3.7061848745	-3.3867985461	-4.6535988996
H	-4.6861177179	-1.9213493174	-4.7296423914
H	-5.1664099325	-3.2561218075	-3.6592701633
H	6.3238889942	0.3044474024	-0.2898079524
H	5.0651449160	3.2337714232	-1.3722673023
H	6.4256412526	2.4784055541	-0.5205704820
H	5.2172459374	3.4205390885	0.3734825185
H	5.8780752256	-2.3844899634	1.2822376796
H	6.7133252724	-1.8647187275	-0.1917328755
H	5.4163467200	-3.0883472258	-0.2585565108
H	2.6783302101	-4.6677250216	3.1869795391
H	4.1062739562	-1.2947935964	2.4623982960
H	1.6510275789	-5.4267319709	-0.8911034276
H	2.3035481536	-1.9264954360	-1.9194315278
H	1.8194411853	-6.1934068037	1.4472769429
H	1.6845517100	-0.8818190684	2.6901522652
H	2.5139616616	-0.6494229018	4.2448022757
H	1.5727838828	-2.1314325382	3.9462837525
H	3.7081471754	-3.4205099282	4.6352375484
H	4.6908430559	-1.9573735946	4.7188616024
H	5.1658990925	-3.2855298089	3.6379621156
H	1.2070323522	-4.6345515163	-2.8561177951
H	1.0060663141	-3.0537304756	-3.6166678684
H	0.2358936323	-3.3864241631	-2.0530965775
H	4.5300460137	-2.8628277964	-2.3364262328

H	3.5409212220	-3.0362396009	-3.7953272374
H	3.8313344823	-4.4438138740	-2.7492489655

102

$^{1}(\mathbf{2}^{\mathbf{N_2}})^{-}$ E_{tot}(r^{2}SCAN-3c) = -4972.533131 $\langle S^2 \rangle$ = 0.000

Ni	-1.9436562936	-0.0000000000	0.0000000000
Ni	1.9436562936	0.0000000000	-0.0000000000
N	-0.6739027879	1.3669883221	0.0393967962
N	-3.2033620270	1.4947447707	0.1466670661
N	-3.3498990260	-1.3308083495	-0.1609572781
N	-0.5855765869	-1.3210878800	-0.0132574331
N	0.5855790374	-1.3210909948	0.0131793174
N	0.6739041171	1.3669876027	-0.0393466614
N	3.2033607792	1.4947397019	-0.1466817236
N	3.3498941372	-1.3308099828	0.1609418824
C	-1.1004022715	2.6415231321	0.0664346452
C	-2.5776319961	2.8227138146	0.1632083397
C	-4.5135644349	1.4074764787	0.2810649295
C	-4.6536482425	-1.0839905031	-0.0416593362
C	-2.9671084149	-2.6487211929	-0.5329148343
C	1.1004033701	2.6415218944	-0.0664144236
C	0.0000004688	3.5039865599	-0.0000000000
C	-5.2006348309	0.1829876681	0.2146685900
C	-5.3450004888	2.6528285139	0.5099964556
C	-5.6511166717	-2.2101158663	-0.2372244892
C	-2.4480320044	-3.5245350949	0.4406161906
C	-3.0280893887	-3.0326078782	-1.8862277669
C	2.5776327856	2.8227104361	-0.1632031620
C	-2.0662517624	-4.8083515235	0.0479808021
C	-2.3258109471	-3.0892905289	1.8887402261
C	-2.6392979214	-4.3264962102	-2.2349766787
C	-3.4159247606	-2.0281774021	-2.9570971930
C	4.5135633147	1.4074708752	-0.2810798584
C	4.6536442367	-1.0839958920	0.0416424226
C	2.9670999602	-2.6487281074	0.5328783443
C	-2.1739716301	-5.2164554933	-1.2743770752
C	-1.0473942312	-3.6072972138	2.5518075263
C	-3.5646990566	-3.4953614144	2.7016702060
C	-2.1513863142	-1.3456777799	-3.5021061648
C	-4.2377998349	-2.6373715831	-4.0976319651
C	5.2006326570	0.1829813996	-0.2146845207
C	5.3450013908	2.6528218280	-0.5100120192
C	5.6511115947	-2.2101249244	0.2371979579
C	3.0280749425	-3.0326383930	1.8861850171
C	2.4480322473	-3.5245268078	-0.4406724356
C	2.6392980028	-4.3265376955	2.2349087532
C	3.4158948604	-2.0282221036	2.9570729878
C	2.0662645518	-4.8083547440	-0.0480603056
C	2.3258239306	-3.0892578542	-1.8887897712
C	2.1739841997	-5.2164833105	1.2742900602
C	2.1513441474	-1.3457697267	3.5021091786
C	4.2377967426	-2.6374219180	4.0975845533
C	1.0474337715	-3.6072850850	-2.5518941309
C	3.5647374308	-3.4952784761	-2.7017060709
H	-2.8269905113	3.3785526240	1.0843209919
H	-2.9387993987	3.4425287910	-0.6765694130
H	-0.0000002205	4.5850195540	-0.0000138856
H	-6.2775936841	0.2251139586	0.3309223753
H	-5.0463643302	3.1616668391	1.4347777827
H	-6.4055307498	2.4035063157	0.5838915901
H	-5.2170911906	3.3736743365	-0.3068853278
H	-5.7876235129	-2.4177337229	-1.3061743086
H	-6.6235811902	-1.9365874456	0.1808558262
H	-5.3096005493	-3.1436397493	0.2180731334
H	2.8269807109	3.3785590863	-1.0843126899
H	2.9388136693	3.4425138433	0.6765771723
H	-1.6574355433	-5.4929670203	0.7859160691
H	-2.2770363500	-1.9936335972	1.8865193786
H	-2.6823438829	-4.6398850161	-3.2748398328
H	-4.0235786148	-1.2473199194	-2.4865779082
H	-1.8656769265	-6.2181996540	-1.5631727708
H	-1.0761958913	-4.6918623113	2.7154011440

H	-0.9212226640	-3.1344926700	3.5328971231
H	-0.1673070256	-3.3771362355	1.9446536595
H	-3.7102292540	-4.5828088148	2.6711789796
H	-4.4700842308	-3.0171748717	2.3148724928
H	-3.4482959877	-3.1955632239	3.7504887044
H	-1.5915182733	-0.8657988780	-2.6910964213
H	-2.4122944299	-0.5796003758	-4.2427789398
H	-1.4948599379	-2.0829146943	-3.9791249296
H	-3.6406652564	-3.3246651726	-4.7081095472
H	-4.6006531567	-1.8451665679	-4.7626027724
H	-5.1036835546	-3.1920710115	-3.7181612678
H	6.2775910774	0.2251048547	-0.3309416544
H	5.0463701814	3.1616566724	-1.4347968677
H	6.4055317456	2.4034983422	-0.5839012953
H	5.2170882810	3.3736706574	0.3068663223
H	5.7876247185	-2.4177509998	1.3061452465
H	6.6235743111	-1.9365943878	-0.1808853866
H	5.3095924851	-3.1436442963	-0.2181068516
H	2.6823455727	-4.6399446316	3.2747665292
H	4.0235243925	-1.2473400119	2.4865647622
H	1.6574570489	-5.4929603011	-0.7860096814
H	2.2770192093	-1.9936021480	-1.8865462105
H	1.8657036300	-6.2182382330	1.5630630992
H	1.5914397840	-0.8659119792	2.6911122607
H	2.4122401168	-0.5796809624	4.2427747123
H	1.4948557605	-2.0830299198	3.9791439930
H	3.6406893962	-3.3247469932	4.7080538905
H	4.6006380664	-1.8452243498	4.7625711427
H	5.1036908949	-3.1920889849	3.7180900327
H	1.0762633521	-4.6918482457	-2.7154974087
H	0.9212769743	-3.1344749045	-3.5329833377
H	0.1673225630	-3.3771508763	-1.9447651675
H	4.4701020151	-3.0170746609	-2.3148816961
H	3.4483448440	-3.1954614032	-3.7505202641
H	3.7102978131	-4.5827224715	-2.6712345969

100

^{3}**4**$^{+}$ E_{tot}(r^{2}SCAN-3c) = -4862.760301 $\langle S^2 \rangle$ = 2.136

Ni	-2.0943196488	-0.0000000000	0.0000000000
Ni	2.0943196488	0.0000000000	-0.0000000000
N	-0.6865973019	1.2219443017	-0.3006556108
N	-3.2328597080	1.4318429534	-0.2350769285
N	-3.3937622489	-1.2150766172	0.2847734303
N	0.6683121195	1.1875882514	-0.3759925229
N	3.2602065290	1.3976266906	-0.6413972962
N	3.4168836158	-1.2292268847	0.3288982747
C	-1.0993104771	2.4510864409	-0.6368203478
C	-2.5636702430	2.6920064816	-0.5897320247
C	-4.5313604857	1.4286560754	0.0487096146
C	-4.7002319255	-1.0131635987	0.4366881797
C	-2.8382673513	-2.4917232101	0.0084588655
C	1.0916834735	2.4161465163	-0.7778660485
C	0.0020691123	3.2547448375	-0.9524039190
C	-5.2423193672	0.2685719976	0.3860680637
C	-5.2931367810	2.7292184796	0.0000000000
C	-5.5935831181	-2.2007995093	0.6627512803
C	-2.1331158799	-3.1638964030	1.0334144885
C	-3.0242461785	-3.0696166952	-1.2728486833
C	2.5617543044	2.6431752857	-0.9566265209
C	-1.6725020680	-4.4520407786	0.7733384831
C	-1.8775952358	-2.4825595226	2.3613468742
C	-2.5451993217	-4.3600061086	-1.4772037696
C	-3.6649968214	-2.2851548521	-2.3999399036
C	4.5554196027	1.2769251288	-0.8306186757
C	4.7195201864	-1.0978895424	0.0133682728
C	2.9509686830	-2.4153196340	0.9768272398
C	-1.8904249285	-5.0494922323	-0.4617690859
C	-0.6189071963	-1.6090853013	2.2518530768
C	-1.7296996404	-3.4519066426	3.5366474543
C	-2.5760739419	-1.5010441198	-3.1537640795
C	-4.4715687048	-3.1518857150	-3.3707341758
C	5.2421397740	0.0709844941	-0.5329062153

C	5.3553481027	2.4258552400	-1.3856672455
C	5.6412128005	-2.2630676725	0.2514847385
C	3.0832330660	-2.5254997961	2.3774028610
C	2.3267218766	-3.4153899842	0.2063697868
C	2.5767918713	-3.6700801083	2.9909329193
C	3.7093232287	-1.4072117414	3.1923025325
C	1.8405531213	-4.5421177404	0.8691112306
C	2.1667869052	-3.2549381606	-1.2942146010
C	1.9644674166	-4.6714872648	2.2462413838
C	2.6344139688	-0.3877358716	3.6014268808
C	4.4719113982	-1.9027643523	4.4239104627
C	0.8110047759	-2.6071034942	-1.6191766877
C	2.3168255075	-4.5707790380	-2.0636180579
H	-2.7831172010	3.4818345607	0.1440221549
H	-2.9210840302	3.0508958075	-1.5673377013
H	0.0021722029	4.2902729332	-1.2595861239
H	-6.3002879185	0.3736109292	0.5872791997
H	-4.9350370825	3.4222734402	0.7701718594
H	-6.3553116222	2.5538731580	0.1702498680
H	-5.1778310078	3.2262626579	-0.9691343216
H	-5.6790181892	-2.7980110773	-0.2528071846
H	-6.5929740775	-1.8812617223	0.9604597766
H	-5.1787926839	-2.8574502533	1.4346160765
H	2.7604945133	2.9699208366	-1.9892583971
H	2.8956705664	3.4612701884	-0.2983430143
H	-1.1490783127	-5.0014010673	1.5479638775
H	-2.7287073846	-1.8213230495	2.5683251207
H	-2.6870779275	-4.8416621450	-2.4392566665
H	-4.3503903832	-1.5484069084	-1.9645536872
H	-1.5406838732	-6.0627155450	-0.6397158761
H	0.2544341271	-2.2157453469	1.9868416168
H	-0.4171239682	-1.0879822406	3.1930102923
H	-0.7325186925	-0.8255278851	1.4782492769
H	-0.7997918520	-4.0270658697	3.4708788900
H	-2.5684223253	-4.1530020929	3.5896506897
H	-1.6968418822	-2.8901044075	4.4750350782
H	-2.0197708539	-0.8417160694	-2.4738356819
H	-3.0223873759	-0.8813724060	-3.9386060288
H	-1.8618412402	-2.1888713853	-3.6198314510
H	-3.8268854949	-3.7992727780	-3.9742591427
H	-5.0233576486	-2.5115984300	-4.0655900539
H	-5.1937464396	-3.7851839169	-2.8452635034
H	6.3036299282	0.0645386679	-0.7488100775
H	5.0172856792	2.6807384771	-2.3970322761
H	6.4166843399	2.1814894494	-1.4337400979
H	5.2358959764	3.3230123886	-0.7678821869
H	5.6844687728	-2.5194045351	1.3156799774
H	6.6505760566	-2.0422923501	-0.0969462228
H	5.2719487513	-3.1541343536	-0.2690219877
H	2.6702339559	-3.7886404138	4.0657883701
H	4.4265818097	-0.8804623564	2.5518291597
H	1.3708533157	-5.3368128983	0.2983052782
H	2.9548422003	-2.5765144737	-1.6440974416
H	1.5941550349	-5.5641312574	2.7427518723
H	2.0976274898	0.0016264571	2.7264375657
H	3.0856899735	0.4602442241	4.1272773016
H	1.9012357253	-0.8552484747	4.2681538103
H	3.7973930103	-2.2999219182	5.1894695504
H	5.0202079756	-1.0720420649	4.8789299483
H	5.1919390900	-2.6860841474	4.1657219668
H	-0.0113631637	-3.2567941015	-1.3032880702
H	0.7152755947	-2.4189448118	-2.6939104054
H	0.6850466038	-1.6417881089	-1.1045406703
H	3.2390624589	-5.0928662913	-1.7898459741
H	2.3431555518	-4.3715019256	-3.1395019278
H	1.4752877834	-5.2479165505	-1.8814594501

100

^{5}4$^+$ E_{tot}(r^{2}SCAN-3c) = -4862.757514 $\langle S^2 \rangle$ = 6.042

Ni	-2.0889508340	-0.0000000000	0.0000000000
Ni	2.0889508340	0.0000000000	-0.0000000000
N	-0.6765544041	1.2328565319	-0.2795998175

N	-3.2564253341	1.5341882709	-0.1496450828
N	-3.3998411672	-1.2396292935	0.3156841419
N	0.6765155361	1.2030924389	-0.3880694961
N	3.2563207429	1.3956661598	-0.6546722801
N	3.3998145933	-1.2276850404	0.3595161037
C	-1.0967401033	2.4819167916	-0.5812126928
C	-2.5658077132	2.7707718848	-0.5181648202
C	-4.5488585146	1.5178712862	0.0905180290
C	-4.7007608434	-0.9668546777	0.5403622937
C	-2.9437122143	-2.5964200732	0.2866623911
C	1.0966615969	2.4310640137	-0.7665544590
C	-0.0000517960	3.2807736538	-0.9002065555
C	-5.2235624687	0.3204599126	0.4478566904
C	-5.3582869927	2.7848304374	-0.0000000000
C	-5.6213419015	-2.0971950164	0.9121661731
C	-2.2559645670	-3.1154367816	1.4001592887
C	-3.1470008379	-3.3580370547	-0.8839622851
C	2.5657093348	2.6472950348	-0.9683105734
C	-1.7922531132	-4.4291479819	1.3286909562
C	-1.9951481087	-2.2627168941	2.6274119377
C	-2.6681661609	-4.6667873490	-0.9016574344
C	-3.7968535077	-2.7472910311	-2.1129209652
C	4.5486100212	1.2589017068	-0.8536873728
C	4.7005876005	-1.1078311880	0.0264970119
C	2.9437807041	-2.3801743856	1.0761615791
C	-2.0014845507	-5.2015707538	0.1944744154
C	-0.6139742870	-1.5961303371	2.5235470927
C	-2.0997794485	-3.0433902072	3.9414988998
C	-2.7170109202	-2.1093263219	-3.0018837984
C	-4.6373264383	-3.7413469448	-2.9192845819
C	5.2233076011	0.0466207169	-0.5506009536
C	5.3578818076	2.3946171617	-1.4227153088
C	5.6210708395	-2.2699366913	0.2825834843
C	3.1470585124	-2.4385437320	2.4715141862
C	2.2561959168	-3.3944307933	0.3828105248
C	2.6682085525	-3.5554030606	3.1539768788
C	3.7970958147	-1.2866364418	3.2173120836
C	1.7924549385	-4.4881339592	1.1140605193
C	1.9956104610	-3.2866328835	-1.1077557902
C	2.0015631946	-4.5743154454	2.4836304835
C	2.7174463092	-0.2844114732	3.6568766394
C	4.6375733197	-1.7307758746	4.4177765107
C	0.6145087311	-2.6602126016	-1.3585508806
C	2.1003902744	-4.6282499029	-1.8400823217
H	-2.7501777545	3.5746603317	0.2118518098
H	-2.9135375252	3.1474398016	-1.4935225601
H	-0.0000677281	4.3225669634	-1.1862665481
H	-6.2820222014	0.4201900504	0.6555365472
H	-5.0127807701	3.5199345947	0.7363967788
H	-6.4155803441	2.5922472717	0.1828155869
H	-5.2588951598	3.2448483339	-0.9896556294
H	-5.7085260932	-2.8187053431	0.0924832928
H	-6.6169900476	-1.7268700230	1.1582711226
H	-5.2220911752	-2.6478914947	1.7712025737
H	2.7499318003	2.9664401584	-2.0062666261
H	2.9135767880	3.4687354345	-0.3215105321
H	-1.2671821955	-4.8573620718	2.1764166640
H	-2.7524191173	-1.4691492061	2.6535342676
H	-2.8183101584	-5.2801878727	-1.7844085490
H	-4.4653080648	-1.9425213434	-1.7860679947
H	-1.6466886408	-6.2278412647	0.1638860157
H	0.1791160602	-2.3511471310	2.4898743191
H	-0.4338399787	-0.9362456399	3.3787727551
H	-0.5216135340	-0.9834996027	1.6117621716
H	-1.2731790429	-3.7520869878	4.0600650530
H	-3.0391196483	-3.6017539780	4.0016232219
H	-2.0568241117	-2.3523457165	4.7892256055
H	-2.1238271615	-1.3723570243	-2.4449801134
H	-3.1703711282	-1.6036379942	-3.8610763149
H	-2.0312649836	-2.8778528409	-3.3756435611
H	-4.0160358679	-4.4917535834	-3.4186948797
H	-5.1907597230	-3.2109860858	-3.7004854281

H	-5.3597412726	-4.2660401312	-2.2856262573
H	6.2816543194	0.0263340456	-0.7806057079
H	5.0120943805	2.6508611625	-2.4310843346
H	6.4151546053	2.1356842214	-1.4819406305
H	5.2586720419	3.2953428513	-0.8064710777
H	5.7083466689	-2.4729613750	1.3555349045
H	6.6166927665	-2.0768722048	-0.1180223336
H	5.2216773826	-3.1815252627	-0.1757928268
H	2.8183302999	-3.6330319503	4.2261197232
H	4.4655979807	-0.7610975036	2.5257635033
H	1.2674682273	-5.2887551539	0.6030829626
H	2.7529547314	-2.6172916921	-1.5347297707
H	1.6467365370	-5.4415832460	3.0331693747
H	2.1242477719	0.0657577930	2.8020975608
H	3.1709609506	0.5885998956	4.1381663903
H	2.0316533444	-0.7548972974	4.3702571220
H	4.0162677414	-2.1215950488	5.2300331632
H	5.1911489921	-0.8763018674	4.8193621742
H	5.3598532135	-2.5053284798	4.1401319629
H	-0.1786823047	-3.2924258999	-0.9446268866
H	0.4345326916	-2.5287832397	-2.4307648330
H	0.5222077926	-1.6682065189	-0.8867672759
H	3.0397370692	-5.1391653188	-1.6069684844
H	2.0575863895	-4.4660560000	-2.9216981811
H	1.2737984359	-5.2984071518	-1.5808395963

100

$^{1}\mathbf{4}^{+}$ E_{tot}(r^{2}SCAN-3c) = -4862.760093 $\langle S^2 \rangle$ = 0.000

Ni	-2.1180339713	-0.0000000000	0.0000000000
Ni	2.1180339713	0.0000000000	-0.0000000000
N	-0.6783194181	1.2107850730	-0.2362028277
N	-3.2433071086	1.4391875926	-0.1949579345
N	-3.4353707332	-1.2016083346	0.2289269540
N	0.6784262808	1.2096041142	-0.2423230859
N	3.2433989510	1.4053127694	-0.3665075157
N	3.4354247407	-1.1984318571	0.2456436950
C	-1.0929578437	2.4709484927	-0.4752924657
C	-2.5581386307	2.7083304347	-0.4771671194
C	-4.5517390829	1.4344709189	0.0351484077
C	-4.7464887928	-1.0093010478	0.3430134747
C	-2.8530399056	-2.4628625091	-0.0457977057
C	1.0930450957	2.4661014375	-0.5000051739
C	0.0000319833	3.3116574101	-0.6540656181
C	-5.2864415078	0.2716023901	0.3064518412
C	-5.3020003109	2.7425752383	-0.0000000000
C	-5.6390184844	-2.2066648872	0.5129963475
C	-2.1956700575	-3.1478804482	1.0033572825
C	-2.9666534412	-3.0165337220	-1.3467471323
C	2.5582240021	2.6863783092	-0.5884420553
C	-1.6998241208	-4.4219168793	0.7392482066
C	-2.0407726800	-2.4973085897	2.3624327695
C	-2.4469145755	-4.2904388843	-1.5555354622
C	-3.5904320258	-2.2291419324	-2.4815403943
C	4.5517961238	1.3133865662	-0.5776776086
C	4.7465588511	-1.0638317423	0.0670485231
C	2.8534896394	-2.2600273697	0.9803706431
C	-1.8339006050	-4.9906041128	-0.5215322904
C	-0.8008676308	-1.5893117766	2.3620581489
C	-1.9508626034	-3.4975377256	3.5174991084
C	-2.5042008579	-1.4079012058	-3.1979714387
C	-4.3510446994	-3.1018258710	-3.4839571698
C	5.2864681676	0.1346806329	-0.3864368683
C	5.3020786327	2.5365587187	-1.0426493122
C	5.6392338672	-2.2356711026	0.3655957663
C	2.9674973369	-2.2762706137	2.3940944294
C	2.1960992645	-3.2931988178	0.2715185962
C	2.4480408696	-3.3746050443	3.0726324107
C	3.5913462783	-1.1158676510	3.1431838460
C	1.7005592253	-4.3705766785	1.0012300193
C	2.0408999563	-3.2094628998	-1.2328824713
C	1.8349440643	-4.4159726648	2.3835662274
C	2.5050824663	-0.0838680131	3.4933085374

C	4.3526194925	-1.5408449721	4.4020855736
C	0.8016664606	-2.3687056261	-1.5781959545
C	1.9495696566	-4.5742584168	-1.9196645633
H	-2.8095441032	3.4685255123	0.2767015522
H	-2.8783554027	3.1016691192	-1.4545772170
H	0.0000188329	4.3710007249	-0.8631546827
H	-6.3505848602	0.3768645544	0.4706828321
H	-4.9748375179	3.4035874039	0.8109269069
H	-6.3717209979	2.5685393096	0.1156539745
H	-5.1395875010	3.2744035455	-0.9434212519
H	-5.6691066800	-2.7993354329	-0.4090161982
H	-6.6558349345	-1.8986185282	0.7591546715
H	-5.2603123355	-2.8640399956	1.3029236481
H	2.8097667729	3.1025629116	-1.5748154616
H	2.8783485986	3.4220019758	0.1658505870
H	-1.2215457372	-4.9867062518	1.5317028690
H	-2.9197884253	-1.8618130019	2.5291070762
H	-2.5303253838	-4.7535948769	-2.5334623514
H	-4.3035253198	-1.5147144860	-2.0537896972
H	-1.4603393762	-5.9950240675	-0.7019087172
H	0.1006163851	-2.1619738976	2.1201491194
H	-0.6628883466	-1.1118318401	3.3377882679
H	-0.8946400669	-0.7696058855	1.6250254028
H	-1.0078335838	-4.0544522723	3.4987403028
H	-2.7763865489	-4.2157732079	3.4954066804
H	-1.9914237606	-2.9628028117	4.4711318046
H	-1.9839340558	-0.7412601776	-2.4971645493
H	-2.9486301767	-0.7907900045	-3.9857784082
H	-1.7621611011	-2.0705082336	-3.6570977554
H	-3.6757500370	-3.7260623314	-4.0784624117
H	-4.8990989159	-2.4650683438	-4.1849607402
H	-5.0717773106	-3.7586346831	-2.9860823796
H	6.3506128437	0.1695710576	-0.5783770782
H	4.9751288733	2.8393098536	-2.0441613878
H	6.3718298867	2.3317128713	-1.0831299656
H	5.1393921567	3.3872919412	-0.3725485093
H	5.6701922356	-2.4320926754	1.4438977816
H	6.6557946374	-2.0447754377	0.0198058583
H	5.2600776153	-3.1445047297	-0.1137680387
H	2.5317544406	-3.4302266262	4.1532411605
H	4.3040594635	-0.6180975069	2.4752478870
H	1.2223060364	-5.1947642705	0.4838269919
H	2.9202655768	-2.6861661739	-1.6293610943
H	1.4615400411	-5.2759252615	2.9331058653
H	1.9843556727	0.2655564600	2.5916667003
H	2.9495897774	0.7868406055	3.9865293586
H	1.7634141372	-0.5217973332	4.1705215379
H	3.6777443499	-1.8916886637	5.1898539923
H	4.9007256830	-0.6849933277	4.8074581890
H	5.0733720853	-2.3376614857	4.1915483840
H	-0.1000242379	-2.8047486017	-1.1355400216
H	0.6630512127	-2.2993884296	-2.6621964932
H	0.8968888928	-1.3298562482	-1.2097915061
H	2.7746264507	-5.2306283582	-1.6258914574
H	1.9898414136	-4.4432668773	-3.0051228517
H	1.0061714138	-5.0811443640	-1.6897420744

102

$^{1}(\mathbf{4}^{N_2})^{+}$ E_{tot}(r^{2}SCAN-3c) = -4972.291426 $\langle S^2 \rangle$ = 0.000

Ni	-2.0318147128	0.0000000000	-0.0000000000
Ni	2.0318147128	-0.0000000000	0.0000000000
N	-0.6769869883	1.3421688697	0.0158232592
N	-3.1940689875	1.4478521554	0.1003876811
N	-3.3662514588	-1.2990783682	-0.0451327851
N	-0.5583888821	-1.1385400205	-0.0001743966
N	0.5583893731	-1.1385445483	0.0001185561
N	0.6769829432	1.3421693328	-0.0158345807
N	3.1940695429	1.4478563635	-0.1003641579
N	3.3662565055	-1.2990700907	0.0451940822
C	-1.0964736010	2.6164349709	0.0284403824
C	-2.5667417394	2.7846548809	0.0798285499
C	-4.5080415521	1.3725926456	0.3143208265

C	-4.6710555563	-1.0897429774	0.0930085536
C	-2.9160769847	-2.6044777726	-0.4384360803
C	1.0964684643	2.6164360543	-0.0284435445
C	-0.0000032895	3.4769844517	0.0000000000
C	-5.2152509544	0.1745101017	0.3262356780
C	-5.2889715951	2.6423023221	0.5469616643
C	-5.6348537913	-2.2396908224	-0.0618802136
C	-2.3917556963	-3.4624991818	0.5472498769
C	-2.9960695419	-2.9788396616	-1.7923132797
C	2.5667374507	2.7846571625	-0.0798159718
C	-1.9857378364	-4.7362730482	0.1486246788
C	-2.3009262830	-3.0452108648	2.0049775475
C	-2.5820374113	-4.2665456822	-2.1364872819
C	-3.4599033830	-2.0045090244	-2.8608340180
C	4.5080460689	1.3725995803	-0.3142678287
C	4.6710656492	-1.0897298272	-0.0928975060
C	2.9160753600	-2.6044583139	0.4385255129
C	-2.0891878045	-5.1394825350	-1.1764765720
C	-1.0024360721	-3.5132808260	2.6706493226
C	-3.5179581502	-3.5438072576	2.7998951965
C	-2.2466550047	-1.2864307420	-3.4734392110
C	-4.3027526150	-2.6650957286	-3.9562501150
C	5.2152614245	0.1745197721	-0.3261392944
C	5.2889781646	2.6423085806	-0.5469078733
C	5.6348678333	-2.2396625186	0.0620794248
C	2.9960092974	-2.9787690607	1.7924223428
C	2.3917796393	-3.4625121995	-0.5471486431
C	2.5819657207	-4.2664627937	2.1366273848
C	3.4597453261	-2.0043774811	2.8609235356
C	1.9857482750	-4.7362712525	-0.1484902430
C	2.3009616404	-3.0452668735	-2.0048891736
C	2.0891561906	-5.1394342183	1.1766282149
C	2.2464235294	-1.2863156093	3.4734043880
C	4.3025316212	-2.6648848592	3.9564354067
C	1.0024513043	-3.5133190384	-2.6705390049
C	3.5179692517	-3.5439309969	-2.7998016093
H	-2.8430161418	3.3634408235	0.9723944287
H	-2.9160863930	3.3514516518	-0.7965525623
H	-0.0000042336	4.5566221895	0.0000045866
H	-6.2842396987	0.2302121629	0.4825666387
H	-4.9713841598	3.1348074106	1.4730540935
H	-6.3523420845	2.4193893634	0.6305306104
H	-5.1513507801	3.3572293474	-0.2709615213
H	-5.8845849237	-2.3665512501	-1.1226554504
H	-6.5611070149	-2.0274905970	0.4756177470
H	-5.2162593756	-3.1852808623	0.2854756114
H	2.8430234281	3.3634448959	-0.9723767858
H	2.9160691327	3.3514527293	0.7965713311
H	-1.5910633325	-5.4266599885	0.8876665665
H	-2.3169461520	-1.9481360086	2.0419750955
H	-2.6406349904	-4.5903865880	-3.1709808242
H	-4.0809230958	-1.2382594127	-2.3826708234
H	-1.7767722040	-6.1392264483	-1.4640878789
H	-0.9883212819	-4.5983794502	2.8189018525
H	-0.9056723075	-3.0518681760	3.6589013744
H	-0.1194835011	-3.2507375993	2.0797937439
H	-3.5948741371	-4.6353826283	2.7420341914
H	-4.4516273285	-3.1164899407	2.4236915273
H	-3.4231479091	-3.2642998449	3.8545162096
H	-1.6675045166	-0.7557214284	-2.7072803719
H	-2.5682976332	-0.5536926276	-4.2211591852
H	-1.5802350341	-2.0061988933	-3.9609985682
H	-3.7026814565	-3.3252453498	-4.5909763626
H	-4.7348142060	-1.8984381684	-4.6069494187
H	-5.1215367153	-3.2570552997	-3.5344534837
H	6.2842542626	0.2302236810	-0.4824401595
H	4.9714195836	3.1347937097	-1.4730206111
H	6.3523521736	2.4193975443	-0.6304369939
H	5.1513263311	3.3572508037	0.2709967686
H	5.8845440494	-2.3664908792	1.1228711699
H	6.5611460675	-2.0274648649	-0.4753761914
H	5.2163044773	-3.1852684890	-0.2852698545

H	2.6405196489	-4.5902638252	3.1711365321
H	4.0807738977	-1.2381353891	2.3827630325
H	1.5910872647	-5.4266815272	-0.8875180109
H	2.3170139368	-1.9481943744	-2.0419276400
H	1.7767221214	-6.1391658429	1.4642623281
H	1.6672901543	-0.7556869424	2.7071766732
H	2.5679837665	-0.5535122072	4.2210962358
H	1.5800119831	-2.0060875188	3.9609692383
H	3.7024350286	-3.3250145731	4.5911583158
H	4.7345264268	-1.8981791460	4.6071230837
H	5.1213633477	-3.2568435546	3.5347295467
H	0.9882944849	-4.5984240412	-2.8187401498
H	0.9057029770	-3.0519503021	-3.6588133139
H	0.1195106975	-3.2507107204	-2.0796952617
H	4.4516552884	-3.1166234319	-2.4236318260
H	3.4231494987	-3.2644619727	-3.8544319730
H	3.5948566317	-4.6355062186	-2.7418995217

102

^{3}(**4**^{N_2})$^+$ E_{tot}(r^{2}SCAN-3c) = -4972.282480 $\langle S^2 \rangle$ = 2.027

Ni	-2.0436960525	0.0000000000	-0.0000000000
Ni	2.0436960525	-0.0000000000	0.0000000000
N	-0.6571859737	1.3202686722	0.0130997874
N	-3.2261748597	1.5356856674	0.1031106763
N	-3.4147422997	-1.2723766859	-0.1532243444
N	-0.4680127268	-1.3889079326	0.0508867208
N	0.6310474739	-1.1807940809	0.0540982822
N	0.7000983177	1.3332871109	-0.0583885655
N	3.2141812976	1.4366410710	-0.1284764474
N	3.3772988098	-1.3044153504	0.1267829024
C	-1.0746767032	2.6051136655	0.0479620691
C	-2.5473478901	2.8369951796	0.1366701081
C	-4.5406903052	1.4915350136	0.1683220021
C	-4.7316906787	-1.0144801779	-0.1004110006
C	-3.0068570107	-2.6151038280	-0.4418134435
C	1.1201422349	2.6018309228	-0.0656624243
C	0.0173434721	3.4626872201	0.0000000000
C	-5.2468488189	0.2689439927	0.0913228294
C	-5.3452058792	2.7562900817	0.3155418050
C	-5.7236693764	-2.1347437527	-0.2891214968
C	-2.5312682587	-3.4236163830	0.6083431439
C	-3.0181216250	-3.0563544358	-1.7781395338
C	2.5879116615	2.7723800348	-0.1370093151
C	-2.1165615132	-4.7181756111	0.2941324401
C	-2.4841815288	-2.9142309149	2.0389629784
C	-2.5900248505	-4.3582036821	-2.0378196486
C	-3.4017726604	-2.1260514569	-2.9163445870
C	4.5362397961	1.3578673219	-0.2872058266
C	4.6846653660	-1.0991845867	0.0177857120
C	2.9146415727	-2.6060736821	0.5240439015
C	-2.1542371054	-5.1864306118	-1.0123328867
C	-1.3203048183	-3.5020008562	2.8424600445
C	-3.8140384487	-3.1600047002	2.7692586498
C	-2.1447668946	-1.4382853886	-3.4731667251
C	-4.1732744863	-2.8272501103	-4.0384630564
C	5.2384398494	0.1584268727	-0.2395647419
C	5.3259312806	2.6212113587	-0.5204723054
C	5.6435826240	-2.2431963552	0.2350973163
C	2.9301414378	-2.9474687503	1.8886152607
C	2.4202065403	-3.4819745837	-0.4610601173
C	2.4747590384	-4.2170356972	2.2472352867
C	3.3618407555	-1.9559624894	2.9549721085
C	1.9763346847	-4.7385606986	-0.0492017128
C	2.3765182555	-3.0911677244	-1.9283208661
C	2.0100110132	-5.1074782853	1.2890976922
C	2.1311373253	-1.2260515983	3.5168874504
C	4.1676873531	-2.6017151683	4.0868589201
C	1.1126972981	-3.5962964012	-2.6334512337
C	3.6324444847	-3.5742096206	-2.6705094999
H	-2.7827445503	3.3846483798	1.0627250727
H	-2.8785338564	3.4741233948	-0.6978304115
H	0.0180722140	4.5426523591	0.0122409229

H	-6.3262003458	0.3423868272	0.1490304901
H	-5.0564870442	3.3005276976	1.2218095293
H	-6.4117423995	2.5389756298	0.3730840323
H	-5.1792689579	3.4293133416	-0.5336034669
H	-5.8355862342	-2.3599446693	-1.3566495008
H	-6.7034135765	-1.8510296924	0.0990624595
H	-5.3929455316	-3.0566258441	0.1941026167
H	2.8547000679	3.3277699131	-1.0482286621
H	2.9417874383	3.3687874191	0.7176556141
H	-1.7525101591	-5.3691283388	1.0822778934
H	-2.3377034756	-1.8246720325	1.9968418588
H	-2.5903788820	-4.7285987838	-3.0582814636
H	-4.0478645668	-1.3359432886	-2.5171281621
H	-1.8309424280	-6.1992153957	-1.2343410688
H	-1.4846963624	-4.5578950805	3.0835608399
H	-1.2215454358	-2.9682335178	3.7933106558
H	-0.3711510421	-3.4245877844	2.3018177601
H	-4.0580401461	-4.2282520785	2.7717071681
H	-4.6427985744	-2.6211043107	2.3014840196
H	-3.7437880742	-2.8244468022	3.8094310029
H	-1.6070177970	-0.8914791412	-2.6881258509
H	-2.4101130351	-0.7256246402	-4.2614706734
H	-1.4583457078	-2.1795276983	-3.8962176235
H	-3.5378417150	-3.5190549797	-4.6007628121
H	-4.5516300622	-2.0870526424	-4.7505004402
H	-5.0251642377	-3.3945220524	-3.6492792971
H	6.3122256997	0.2072166994	-0.3627341407
H	5.0206565109	3.1105029686	-1.4523625672
H	6.3893542123	2.3938042423	-0.5902233200
H	5.1832173088	3.3420653475	0.2917998613
H	5.8616500151	-2.3373726240	1.3063000319
H	6.5856342591	-2.0473171958	-0.2807846673
H	5.2351990952	-3.1993195735	-0.0952536449
H	2.4793202404	-4.5129491160	3.2918356563
H	4.0000606970	-1.1987426113	2.4853552153
H	1.5969207447	-5.4395036775	-0.7859856665
H	2.3716947305	-1.9942253810	-1.9820846852
H	1.6637856156	-6.0925728264	1.5882048439
H	1.5868677411	-0.6950475099	2.7262975894
H	2.4290352780	-0.4916299662	4.2729238370
H	1.4408451350	-1.9383696542	3.9823366463
H	3.5457125791	-3.2501265573	4.7125415652
H	4.5822915735	-1.8264749287	4.7388795717
H	4.9967359141	-3.2031359026	3.6998895087
H	1.1307342344	-4.6825878231	-2.7724243532
H	1.0402117050	-3.1459025981	-3.6288710588
H	0.2041692864	-3.3483330101	-2.0748900737
H	4.5414914839	-3.1178304176	-2.2689340636
H	3.5692681683	-3.3153282255	-3.7327808598
H	3.7325497234	-4.6624653083	-2.5900139448

2.2 Preparation of $LNi_2(PMe_3)_2$ (4^{PMe3})

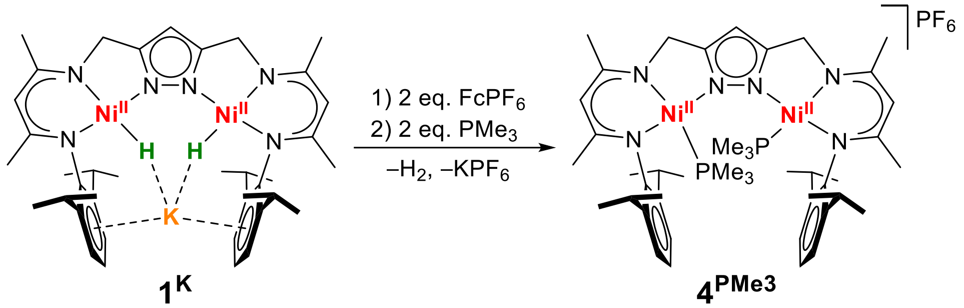

A solution of 1^K (50.0 mg, 65.4 µmol, 1.0 eq.) in THF was added to $FcPF_6$ (43.3 mg, 160.8 µmol, 2.0 eq.) under dinitrogen atmosphere and vigorous stirring. The solution immediately changed color from dark red to dark green with gas evolution. To the reaction mixture PMe_3 (13.9 µl, 10.3 mg, 160.8 µmol, 2.0 eq.) was added and the solution turned dark purple. Dark brown-purple crystals were isolated by diffusing pentane into the THF solution at −35 °C. The crystals were washed with hexane and re-dissolved in THF. All precipitating salts (likely KPF_6) were filtered off and a second crystallization by diffusing pentane into the THF solution at −35 °C gave pure material of 4^{PMe3} (yield 65%).

Further characterization of 4^{PMe3} in solution was not carried out due to its relatively low solubility. However, ^1H NMR spectroscopy in THF-d_8 indicated partial loss of PMe_3 ligands to give paramagnetic species and a dynamic equilibrium between 4^{PMe3} and 4^{THF} (Figure S1111).

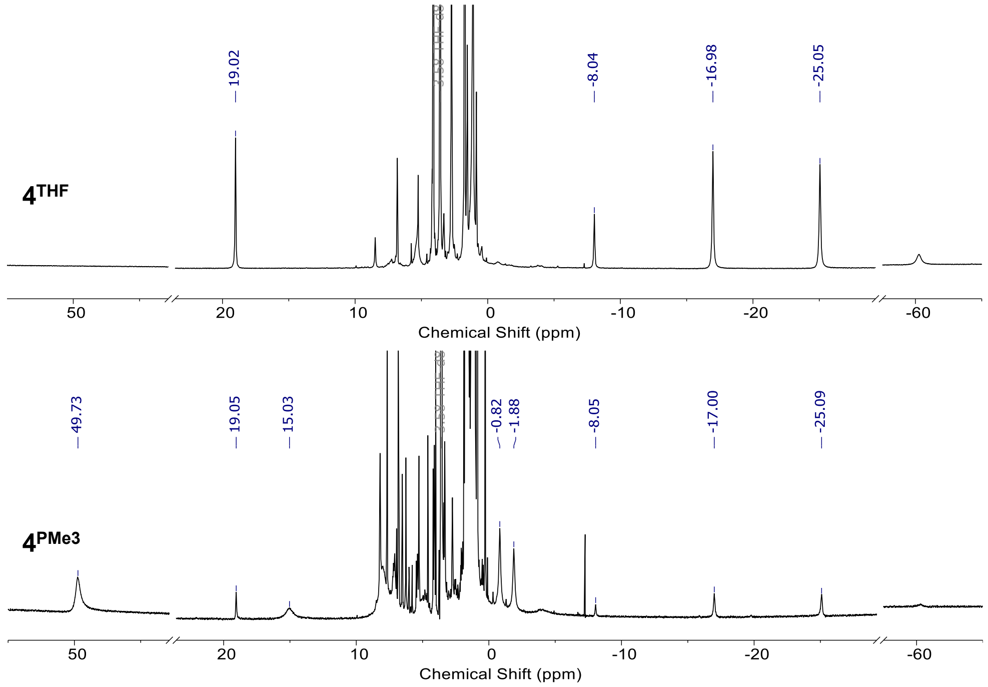

Figure S11. ^1H NMR spectrum in THF-d_8 of crystalline 4^{PMe3} (bottom) and *in situ* generated 4^{THF} (top).

EA: Calc. for **4**$^{\mathbf{PMe3}}$ ($C_{45}H_{71}N_6Ni_2P_3F_6$+1thf, %): C: 53.87, H: 7.29, N: 7.69; Found: C: 53.27, H: 7.29, N: 7.25.

IR [cm^{-1}]: $\tilde{\nu}$ 2960 (w), 2929 (w), 2916 (w), 2867 (w), 1581 (w), 1536 (m), 1514 (m), 1432 (s), 1388 (s), 1357 (s), 1311 (m), 1273 (m), 1249 (m), 1229 (w), 1206 (w), 1187 (w), 1176 (w), 1102 (w), 1096 (w), 1061 (w), 1020 (m), 956 (m), 941 (m), 874 (w), 833 (s) ν_{PF6-}, 803 (s), 768 (m), 751 (m), 729 (m), 716 (m).

SQUID: $S = 0$.

Molecular Structure Determined by X-Ray Diffraction

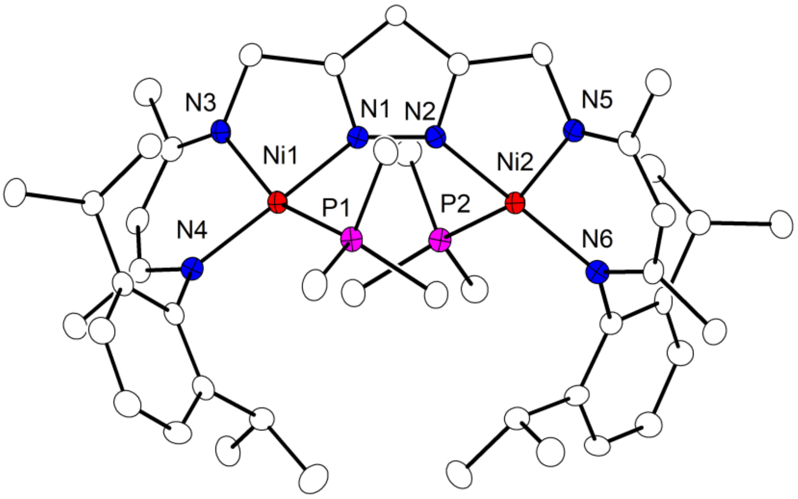

Figure S12. Plot (30% probability thermal ellipsoids) of the cationic part of **4**$^{\mathbf{PMe3}}$ (hydrogen atoms omitted for clarity as well as PF_6^- counterion). Selected bond lengths [Å] and angles [°]: Ni1–N1 1.929(5), Ni1–N3 1.885(5), Ni1–N4 1.912(5), Ni1–P1 2.2707(17), Ni2–N2 1.932(5), Ni2–N5 1.883(5), Ni2–N6 1.897(5), Ni2–P2 2.2624(17), Ni1···Ni2 4.3685(12); N3–Ni1–N4 90.9(2), N3–Ni1–N1 79.6(2), N4–Ni1–N1 163.5(2), N3–Ni1–P1 154.41(16), N4–Ni1–P1 103.52(15), N1–Ni1–P1 90.41(15), N5–Ni2–N6 91.9(2), N5–Ni2–N2 80.4(2), N6–Ni2–N2 163.0(2), N5–Ni2–P2 152.57(15), N6–Ni2–P2 103.49(15), N2–Ni2–P2 89.80(15).

IR Spectroscopy and SQUID Magnetometry Data

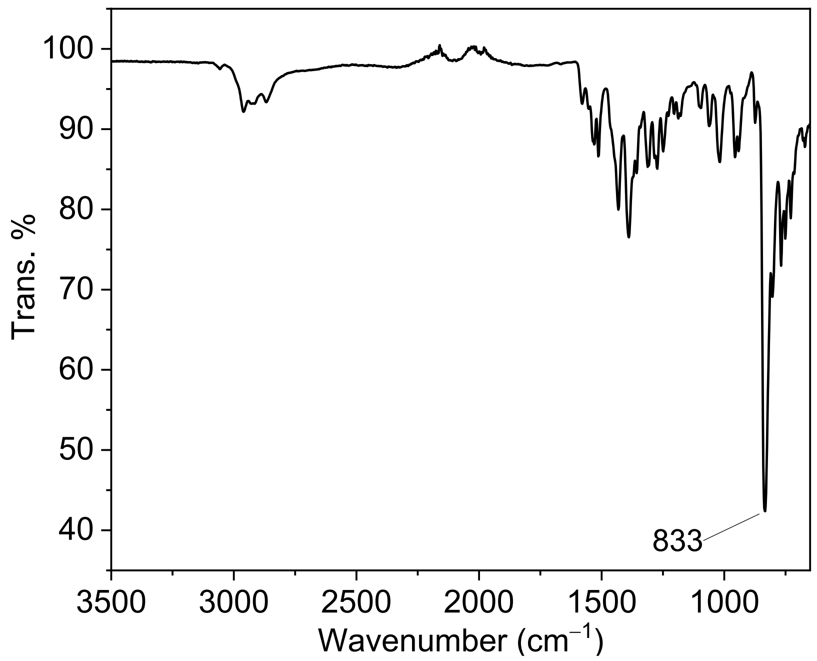

Figure S13. ATR-IR spectrum of solid material of **4**$^{\mathbf{PMe3}}$.

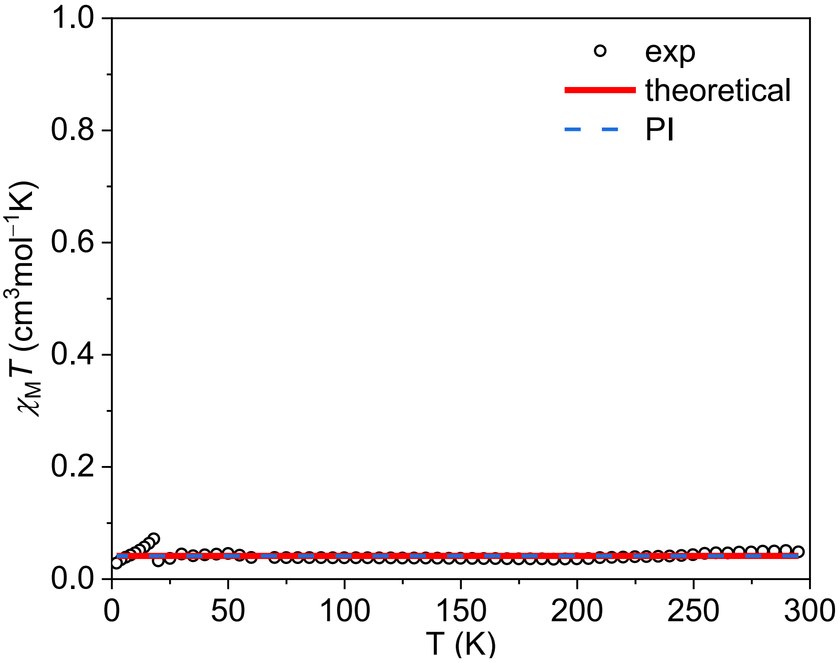

Figure S14. $\chi_M T$ vs. T plot for crystalline **4**$^{\mathbf{PMe3}}$. Experimental data shown as black dots, calculated curve fit assuming $S = 0$ as red line and percentage of impurities in dashed blue line (4.1% with $S = 1$).

2.3 Preparation of [(HL)Ni^{I}Ni^{II}{μ-Me$_2$C$_5$H$_3$N}]X (5)

The following procedures were carried out in a glovebox filled with Ar atmosphere.

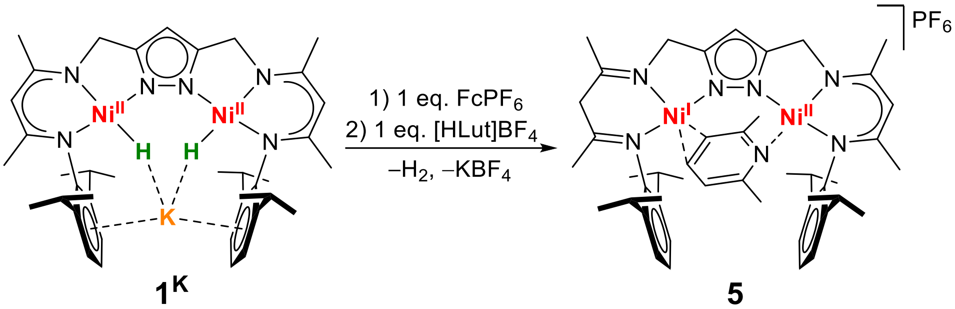

1^{K} **5**

First procedure: A solution of **1**^{K} (7.2 mg, 9.4 µmol, 1.0 eq.) in THF (1 mL) was added to FcPF$_6$ (3.1 mg, 9.4 µmol, 1.0 eq.) under vigorous stirring. The solution immediately changed color from dark red to dark brown with gas evolution, indicating in-situ formation of **3**^{THF}. The suspension was added to [HLut]BF$_4$ (1.8 mg, 9.2 µmol, 0.9 eq.) to give an intense deep red suspension. The reaction mixture was filtered to remove inorganic salts. Crystallization by layering hexane onto the THF solution at –35 °C gave red crystals of **5** (PF$_6^-$ salt) over a period of two weeks (yield: 32%).

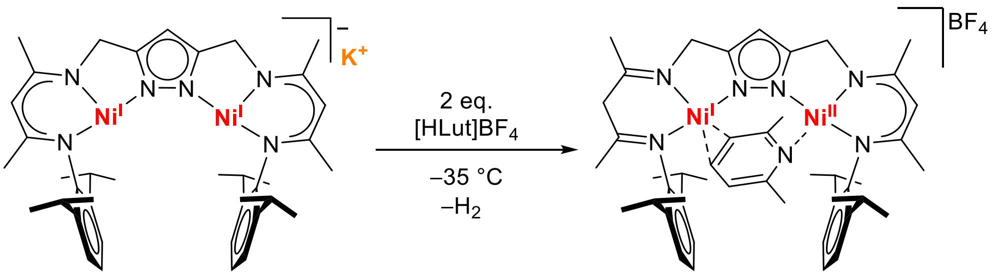

2^{K} **5**

Second procedure: [HLut]BF$_4$ (15.4 mg, 79.1 µmol, 2.0 eq.) was added under vigorous stirring to a precooled solution of **2**^{K} (30.0 mg, 39.6 µmol, 1.0 eq.) in THF (3 mL) at –35 °C, resulting in an intense deep red solution. The reaction mixture was filtered. Crystallization by layering hexane onto the THF solution at –35 °C gave yellow crystals of **5** (BF$_4^-$ salt) over a period of three days (yield: 55%).

IR [cm^{-1}]: $\tilde{\nu}$ = 2962 (w), 2928 (w), 2867 (w), 1654 (w), 1627 (w), 1577 (m), 1560 (m), 1534 (m), 1519 (m), 1460 (m), 1439 (m), 1401 (m), 1370 (m), 1337 (w), 1314 (w), 1309 (w), 1281 (w), 1251 (w), 1229 (w), 1202 (w), 1184 (w), 1161 (w), 1006 (s), 836 $\nu_{PF_6^-}$ (s). (PF$_6^-$ salt of **5**)

UV-vis (THF) [nm]: λ = 376, 496. (PF$_6^-$ salt of **5**)

SQUID: S = 1/2; g = 1.82. (BF$_4^-$ salt of **5**; the g value is too low because of contamination with inorganic salts (mostly KBF$_4$).

EPR (rt, 9.438 GHz, crystalline material, X-band): g = 2.010, 2.186, 2.294. (BF$_4^-$ salt of **5**)

Molecular Structure Determined by X-Ray Diffraction

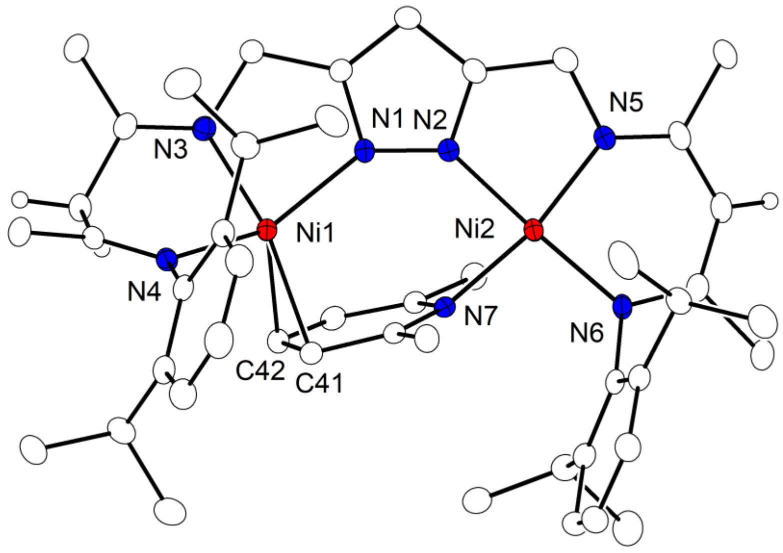

Figure S15. Plot (30% probability thermal ellipsoids) of the cationic part of **5** (most hydrogen atoms omitted for clarity). Selected bond lengths [Å] and angles [°]: Ni1–N1 2.043(2), Ni2–N2 1.895(3), Ni1–N3 2.008(3), Ni1–N4 2.075(2), Ni2–N5 1.878(3), Ni2–N6 1.898(2), Ni1–C41 2.122(3), Ni1–C42 2.152(3), Ni2–N7 1.934(2), C41–C42 1.403(5), Ni1···Ni2 4.3141(5); N3–Ni1–N1 81.10(10), N3–Ni1–N4 88.00(10), N1–Ni1–N4 144.40(10), N3–Ni1–C41 162.03(12), N1–Ni1–C41 109.50(11), N4–Ni1–C41 90.75(11), N3–Ni1–C42 124.60(12), N1–Ni1–C42 113.12(11), N4–Ni1–C42 100.99(11), C41–Ni1–C42 38.32(12), N5–Ni2–N2 84.44(11), N5–Ni2–N6 93.27(11), N2–Ni2–N6 176.04(11), N5–Ni2–N7 164.62(11), N2–Ni2–N7 85.62(10), N6–Ni2–N7 97.27(10).

IR Spectroscopy and UV-vis Spectroscopy and SQUID Magnetometry Data

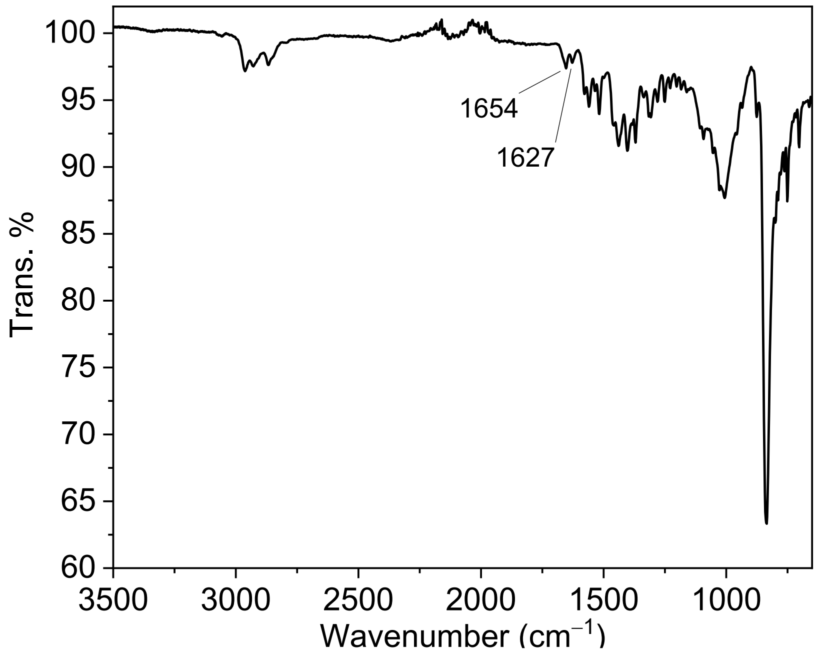

Figure S16. ATR-IR spectrum of solid **5** (PF_6^- salt).

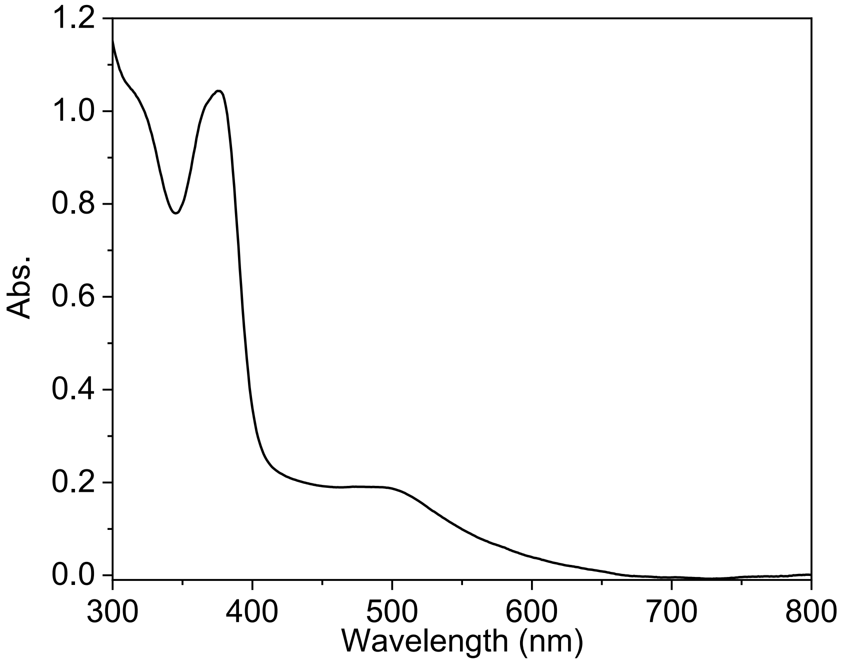

Figure S17. UV-vis spectrum of **5** (PF_6^- salt; 10^{-4} M) in THF at room temperature.

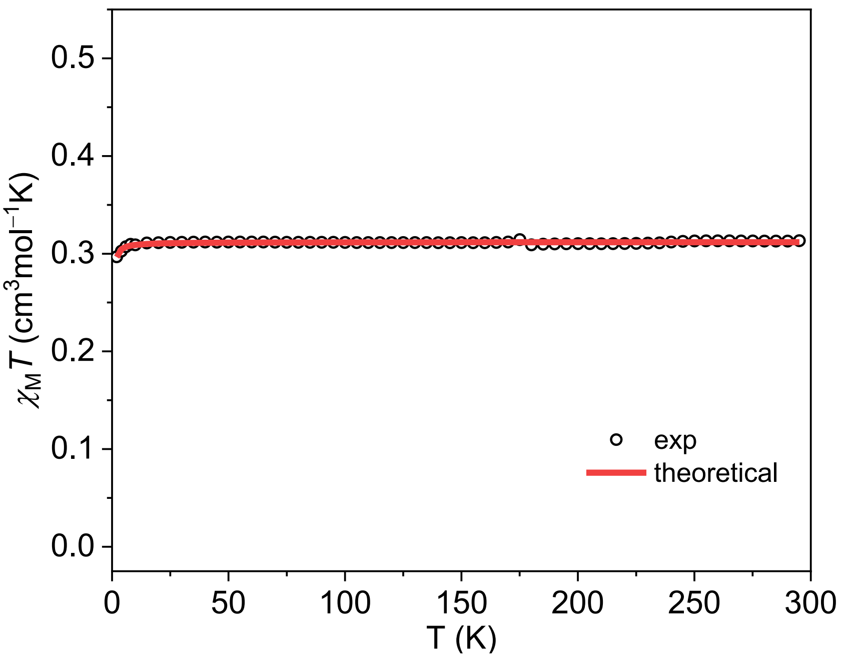

Figure S18. $\chi_M T$ vs. T plot for crystalline **5** (BF_4^- salt). Experimental data shown as black dots, calculated curve fit (with g = 1.82) as red line; the g value is low because of residual KBF_4.

3. Additional Data

3.1 NMR Spectra

Formation of 3^{N2}

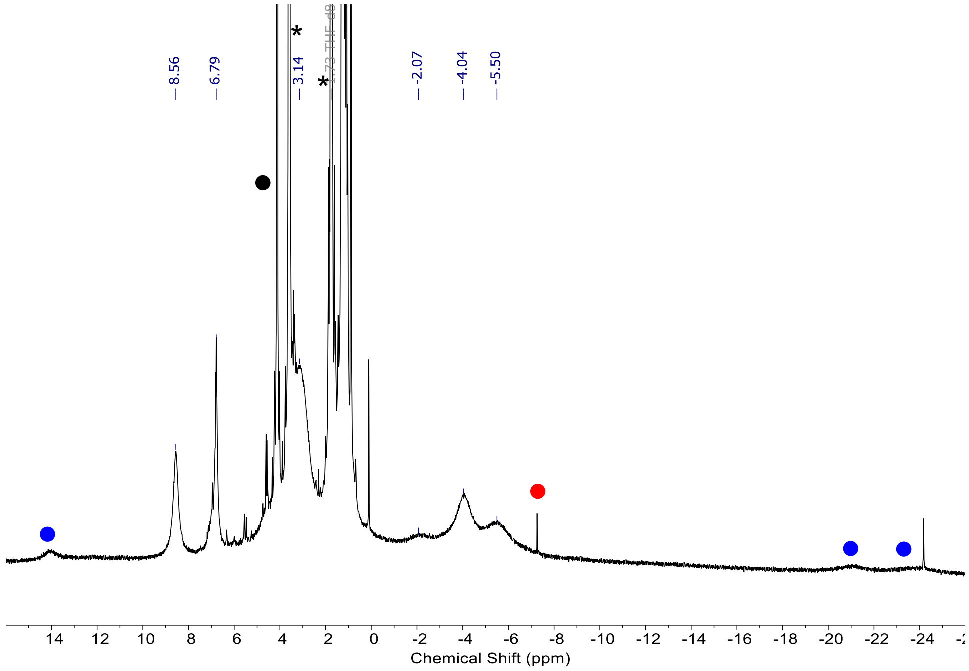

Figure S19. ^{1}H NMR spectrum obtained reacting $\mathbf{2^{K}}$ with 1 eq. of $FcPF_6$ in THF-d_8 under N_2 atmosphere. The peaks assigned to $\mathbf{3^{N2}}$ are picked. Residual solvent (*), ferrocene (●), residual $\mathbf{2^{K}}$ (●) and $LNi_2(\mu\text{-}OH)$ (●) are marked as indicated in brackets.

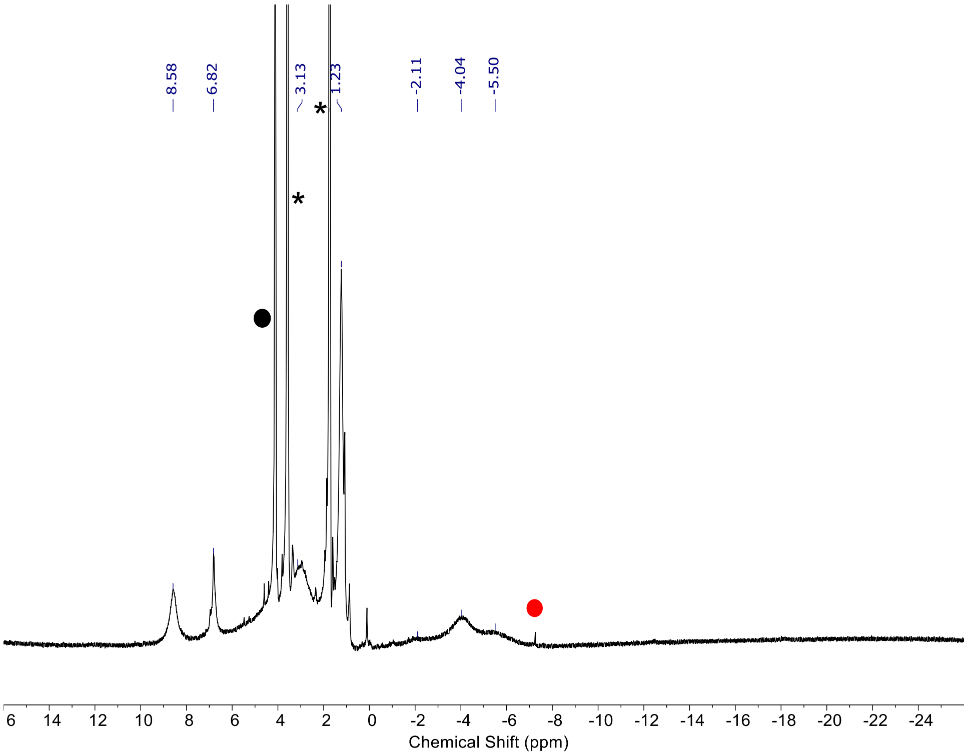

Figure S20. ^{1}H NMR spectrum obtained after reacting **1^K** with 1 eq. of $FcPF_6$ in THF-d_8 under N_2 atmosphere. The peaks assigned to **3^{N2}** are picked. Residual solvent (*), ferrocene (•) and LNi_2(μ-OH) (•) are marked as indicated in brackets.

Reactivity of 3^{N2}

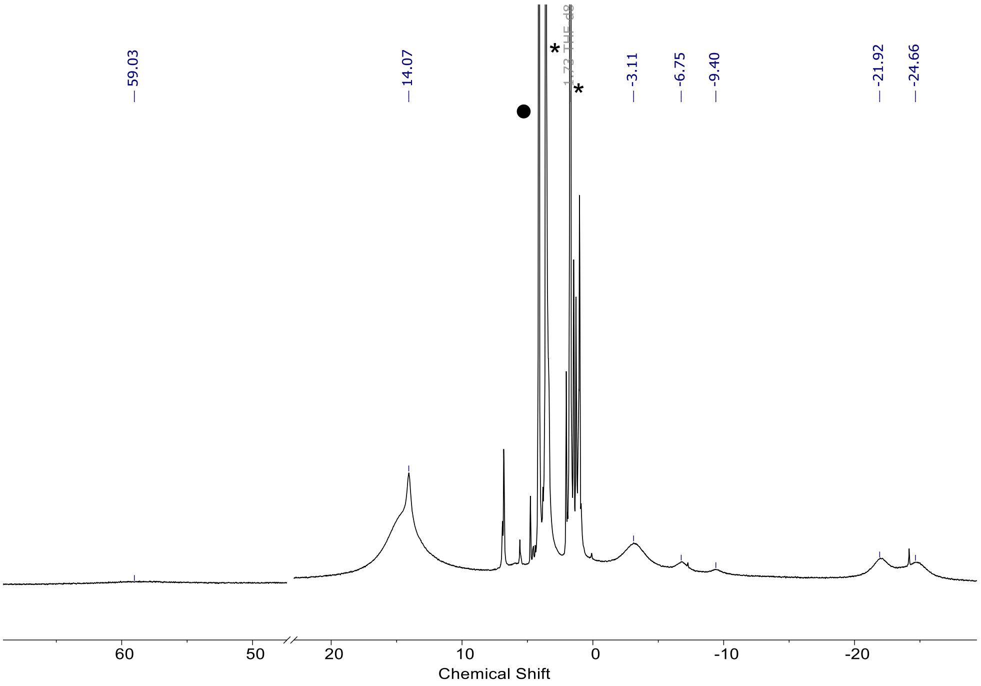

Figure S21. ^{1}H NMR spectrum of *in situ* generated **3^{N2}** after addition of 1.2 eq. of $CoCp_2$* in THF-d_8. Residual solvent (*), and ferrocene (•) are marked as indicated in brackets.

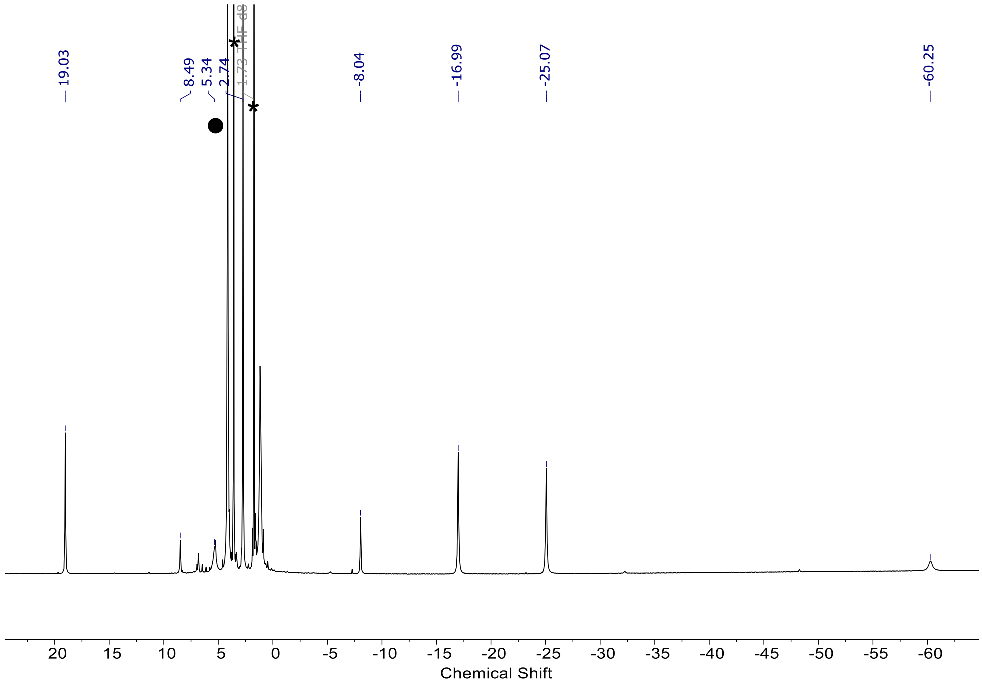

Figure S22. ^{1}H NMR spectrum of in situ generated $\mathbf{3^{N2}}$ after addition of 1 eq. of $FcPF_6$ in THF-d_8. Residual solvent (*) and ferrocene (•) are marked as indicated in brackets.

Formation and Reactivity of $\mathbf{3^{THF}}$

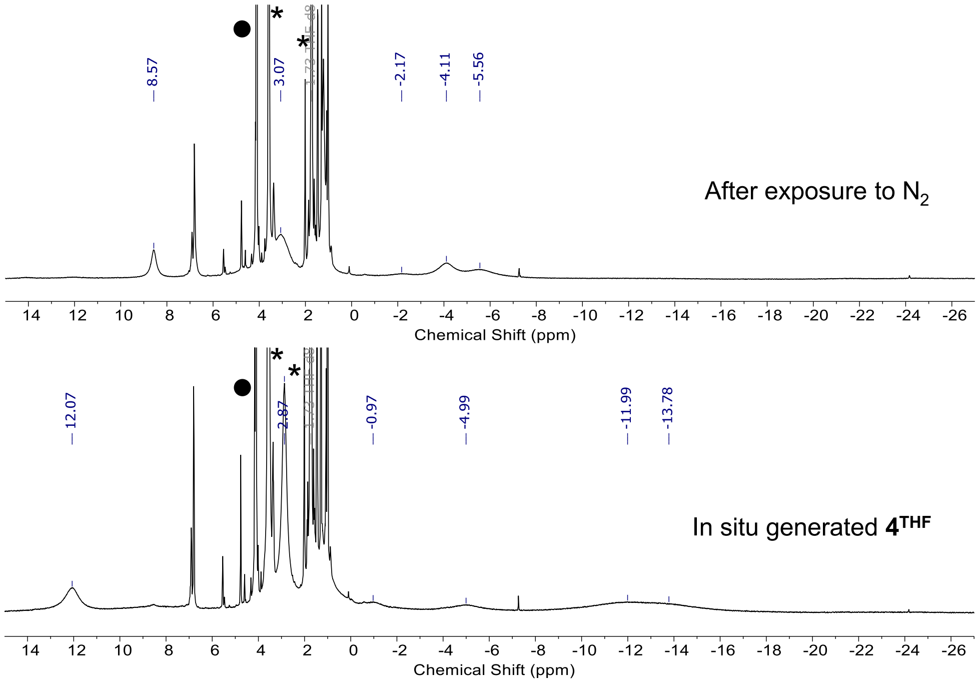

Figure S23. ^{1}H NMR spectrum of *in situ* prepared $\mathbf{3^{THF}}$, generated from the reaction of $\mathbf{1^{K}}$ with 1 eq. of $FcPF_6$ in THF-d_8 under argon atmosphere (bottom). Same sample after exposure to N_2 atmosphere (top). Residual solvent (*) and ferrocene (•) are marked as indicated in brackets.

3.2 EPR Spectrum of 3^{THF}

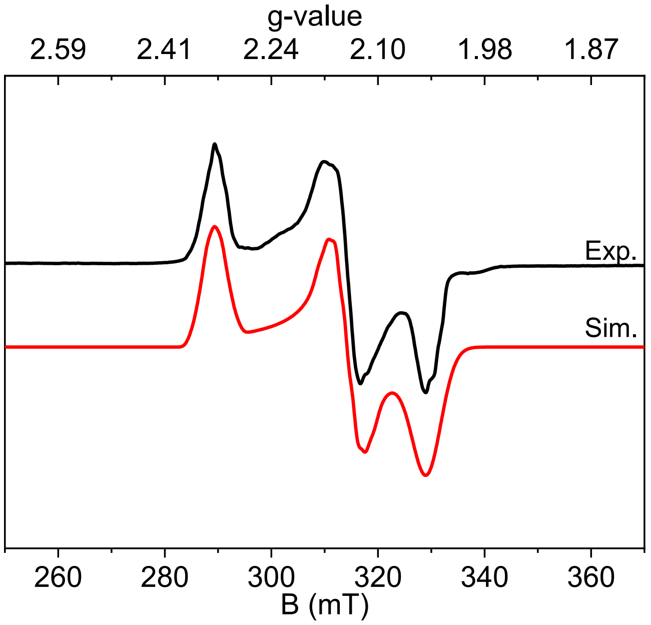

Figure S24. X-band EPR spectrum (9.426 GHz) of *in situ* prepared $\mathbf{3^{THF}}$ in THF (2.5 mM) at 135 K. Experimental data is displayed as black line, while simulated spectrum is reported in red with g = [2.045, 2.143, 2.328].

3.3 GC Measurements

Quantification of the H_2 evolved by the reaction of 1^K with $FcPF_6$ and by the reaction of 1^K with [HLut]BArF. To determine the amount of hydrogen gas a calibration line was recorded. In a nitrogen filled glovebox a J. Young NMR-tube was filled with THF (450 µL) and sealed (always the same NMR-tube was used). A rubber septum was attached, and the headspace was purged with argon. The tube was opened and a defined volume of H_2 (10, 20, 30, 50, 100 and 200 µL) and 200 µL of methane as internal standard were added through the septum. A sample (1 mL) of the gas mixture in the headspace was taken and injected into the GC. In the chromatogram the amount of hydrogen was integrated against the signal of methane. The results of the calibration are depicted in Figure S25.

For samples of the chemical reactions studied, the same NMR tube and 450 µL THF were used, 200 µL of methane was added and the amount of formed hydrogen determined using the calibration line. The results of the reaction of $\mathbf{1^K}$ with one equivalent of $FcPF_6$ and with one equivalent of $[HLut]BAr^F_4$ are listed in Table S5 and Table S6, respectively.

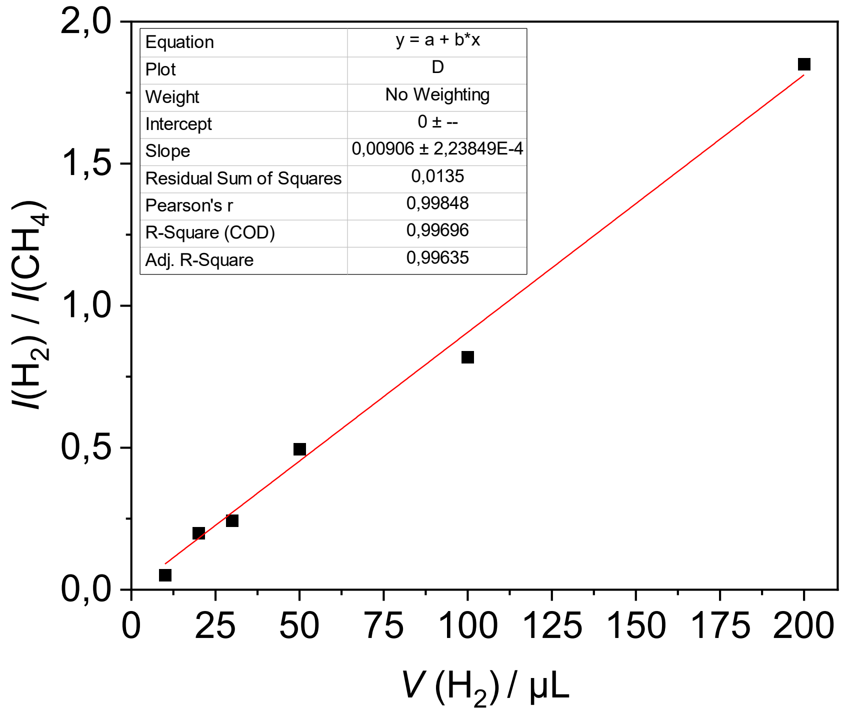

Figure S25. GC calibration curve for quantitative H_2 detection.

Table S5. H_2 quantification of the reaction of **1**^{K} with one equivalent of $FcPF_6$ under N_2, three independent runs.

Entry	*I*(H_2)/*I*(CH_4)	*n* (1^K) / µmol	*n* (H_2) / µmol	eq. H_2 / 1^K
**1**	1.35	5.87	6.09	1.04
**2**	1.29	6.10	5.81	0.95
**3**	1.42	6.37	6.42	1.01

On average 1.0 equiv. of H_2 evolved.

Table S6. H_2 quantification of the reaction of **1**^{K} with one equivalent of [HLut]BArF under N_2, four independent runs.

Entry	*I*(H_2)/*I*(CH_4)	*n* (1^K) / µmol	*n* (H_2) / µmol	eq. H_2 / 1^K
**1**	1.89	5.23	8.54	1.63
**2**	1.08	5.63	4.89	0.87
**3**	0.95	4.33	4.26	0.99
**4**	1.13	4.15	5.09	1.23

The amount of H_2 was found to vary for multiple experiments and to be less than the expected 1.5 equiv. in most cases, because the reaction of **1**^{K} with [HLut]BAr$^F{}_4$ is not clean and gives some minor unknown byproducts; on average 1.2 equiv. of H_2 evolved.

Determination of H_2 released by reaction of 2^K with [HLut]BArF

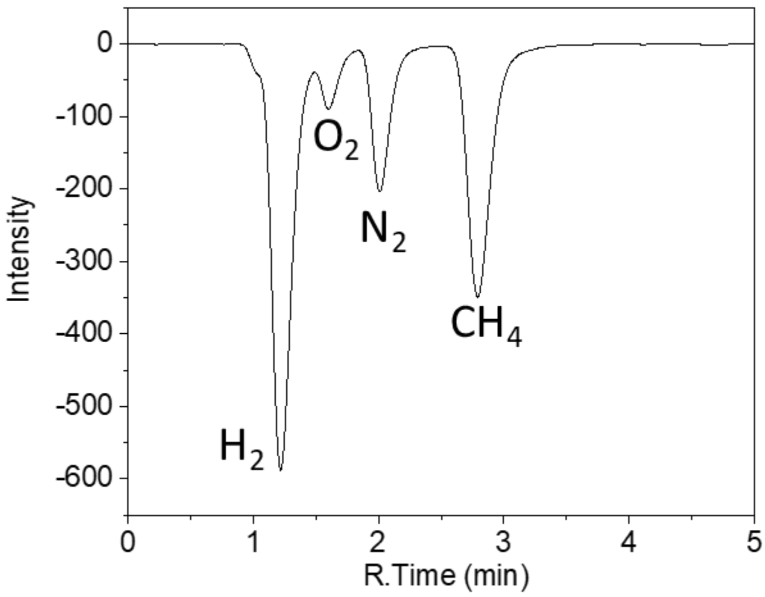

Figure S26. Representative GC trace of the headspace of the reaction of $\mathbf{2^K}$ with 2 eq. of [HLut]BAr$^F{}_4$. O_2 and N_2 derived from purging, methane was added as internal standard.

3.4 CV studies

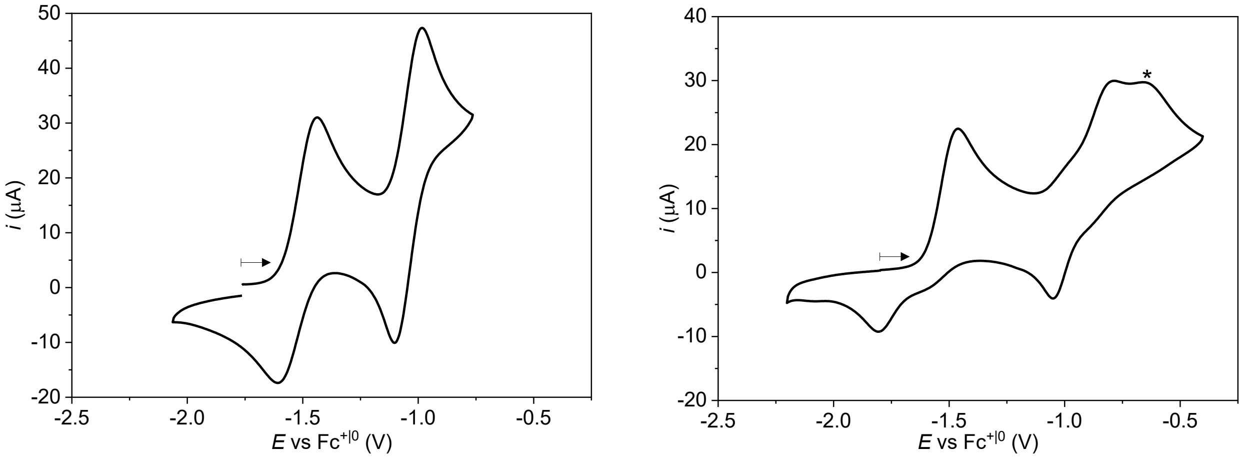

Figure S27. Left: CV of $\mathbf{2^K}$ (3 mM) in THF with 0.2 M $TBAPF_6$ under Ar atmosphere at 100 mVs^{-1}. Right: CV of $\mathbf{2^K}$ (3 mM) in THF with 0.2 M $TBAPF_6$ under N_2 atmosphere at 100 mVs^{-1}. The peak marked with an asterisk originates from an unknown decomposition product.

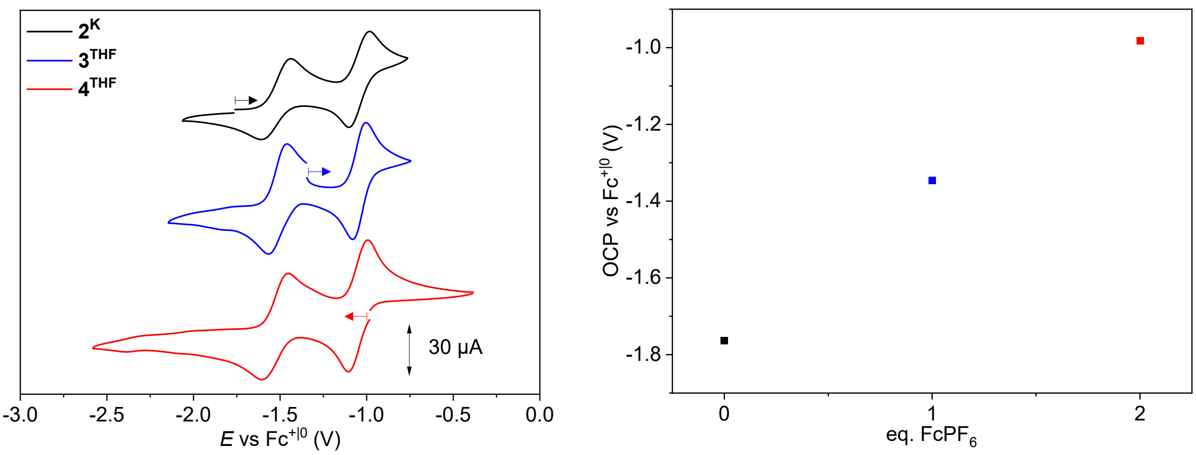

Figure S28. CVs of $\mathbf{2^K}$ (black trace, 3 mM) and $\mathbf{1^K}$ (3 mM) with one equivalent ($\mathbf{3^{THF}}$ blue trace) and two equivalents ($\mathbf{4^{THF}}$ red trace) of $FcPF_6$ in THF with 0.2 M $TBAPF_6$ under Ar atmosphere at 100 mVs^{-1}; Right: OCP vs equivalents of $FcPF_6$.

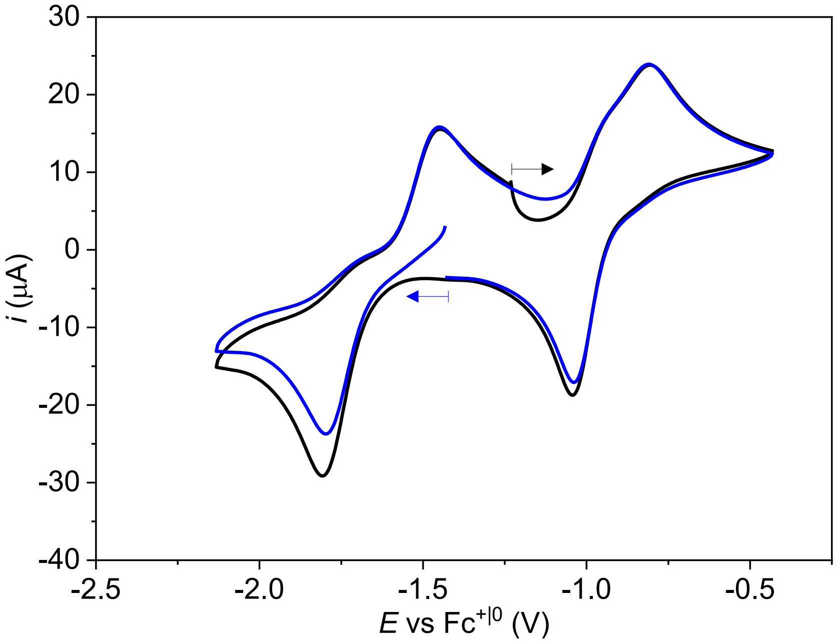

Figure S29. CVs of in situ prepared $\mathbf{3^{N2}}$ (3 mM) in THF with 0.2 M $TBAPF_6$ under N_2 atmosphere at 100 mVs^{-1}.

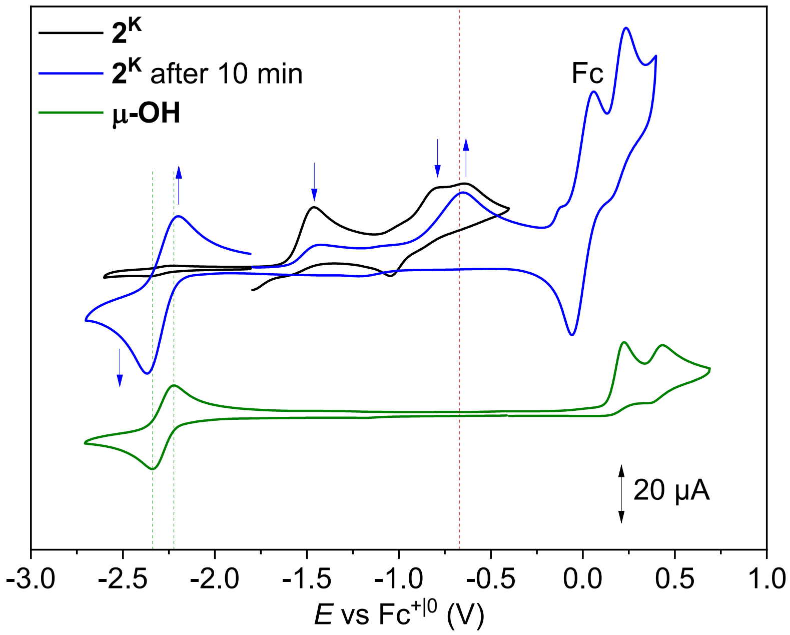

Figure S30. CVs of $\mathbf{2^K}$ (3 mM, black trace), $\mathbf{2^K}$ after 10 min (blue trace, blue arrows indicate the change of the CV upon decomposition) and the decomposition product LNi_2(μ-OH) (1 mM, green trace) in THF with 0.2 M $TBAPF_6$ under N_2 atmosphere at 100 mVs^{-1}.

3.5 IR-Spectroelectrochemistry (IR-SEC)

IR-SEC of 2^K 5 mM in THF 0.2 M $TBAPF_6$ under N_2

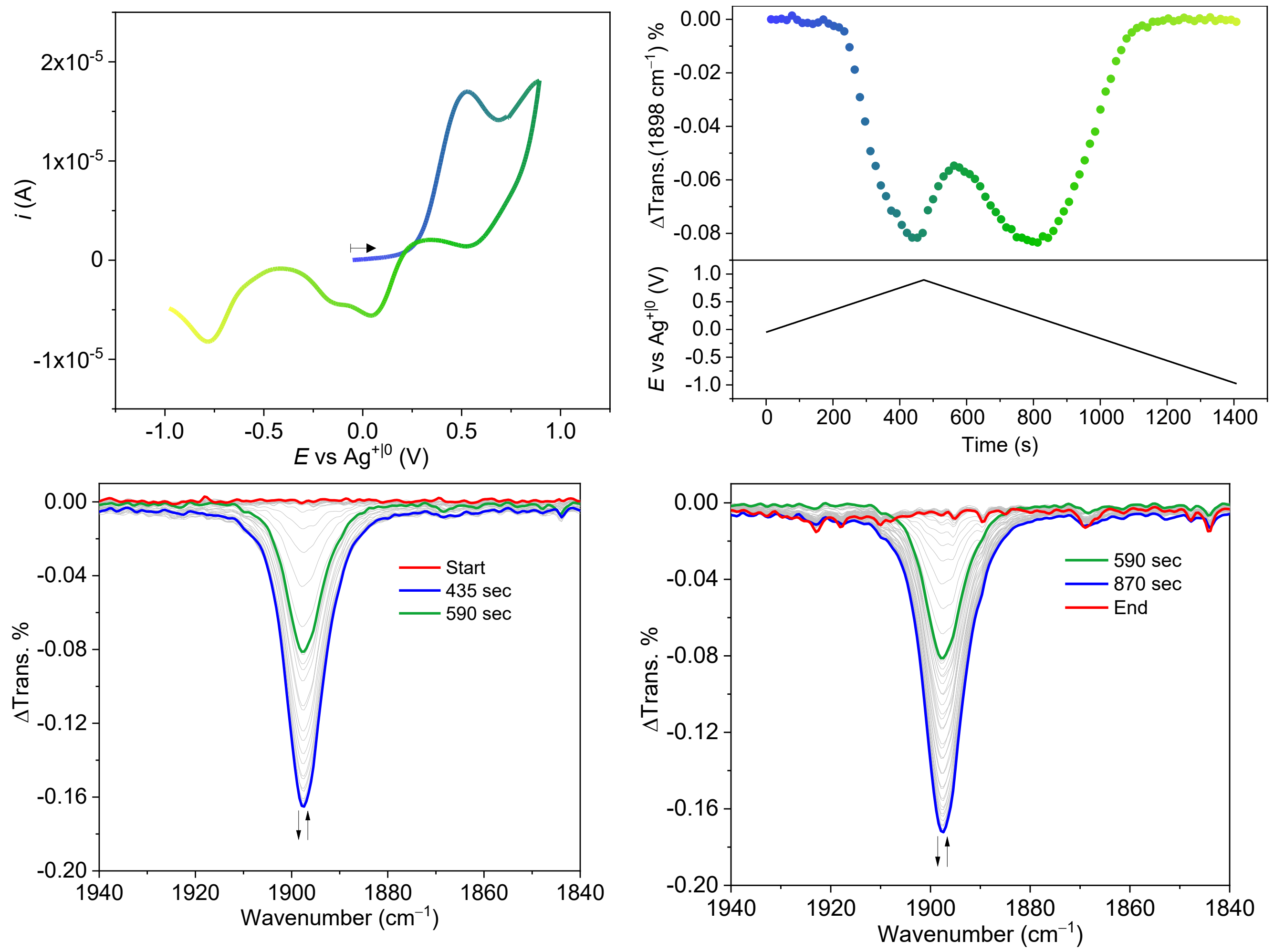

Figure S31. Top left: CV recorded during IR-SEC experiment, $v = 2$ mV·s^{-1}. Top right: difference in transmittance at 1898 cm^{-1} and applied potential vs time of the experiment. Bottom left and right: difference in transmittance of the IR spectra recorded.

IR-SEC of *in situ* generated 3^{N2} (~5 mM in THF 0.2 M $TBAPF_6$) under N_2

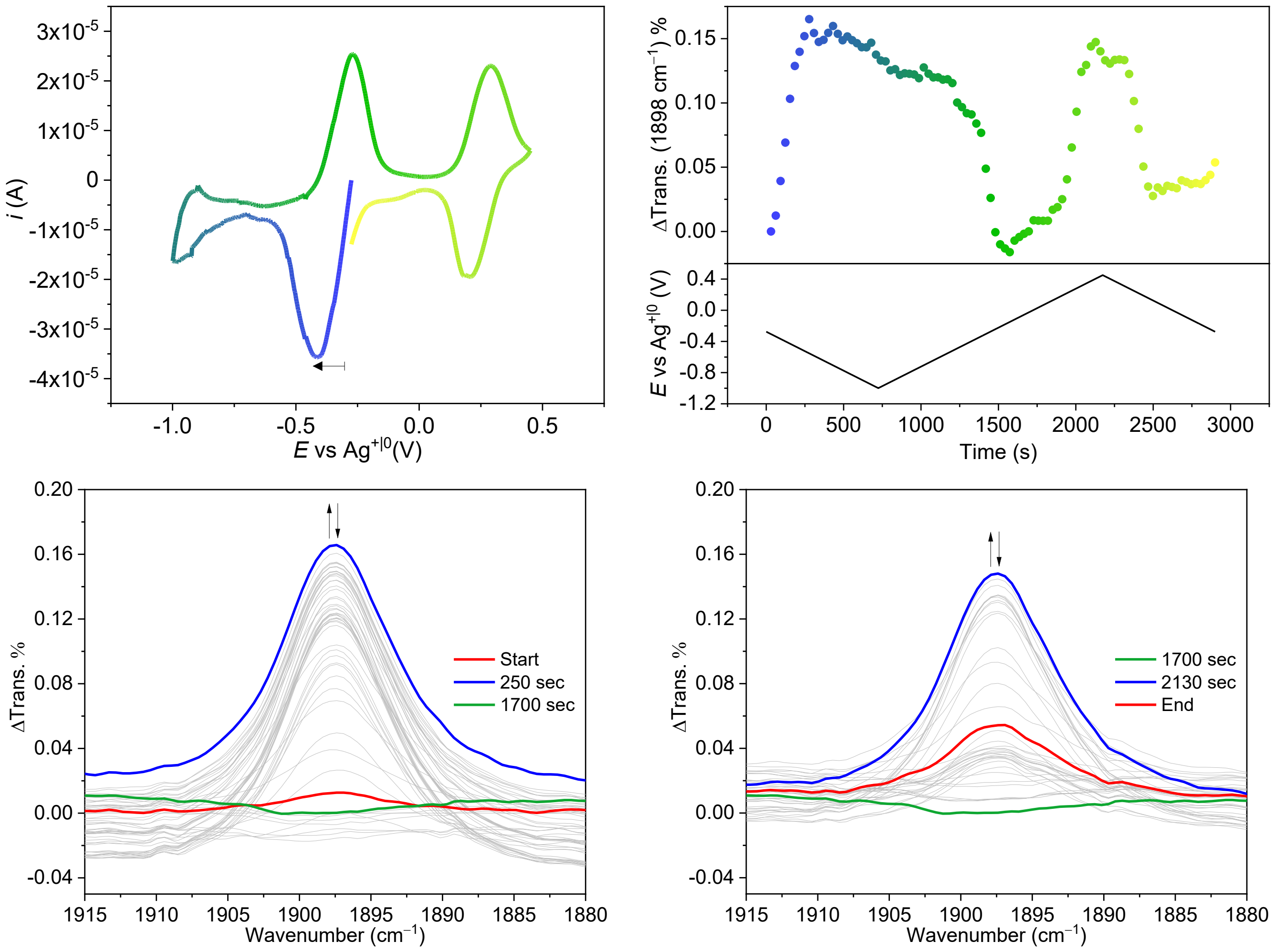

Figure S32. Top left: CV recorded during IR-SEC experiment, $v = 2$ mV·s^{-1}. Top right: difference in transmittance at 1898 cm^{-1} and applied potential vs time of the experiment. Bottom left and right: difference in transmittance of the IR spectra recorded (baseline shift due to gas evolution).

4. References

(1) Thomas, A. M.; Lin, B.-L.; Wasinger, E. C.; Stack, T. D. P. Ligand noninnocence of thiolate/disulfide in dinuclear copper complexes: solvent-dependent redox isomerization and proton-coupled electron transfer. *J. Am. Chem. Soc.* **2013**, *135*, 18912–18919. DOI: 10.1021/ja409603m.

(2) Guo, R.; Qi, X.; Xiang, H.; Geaneotes, P.; Wang, R.; Liu, P.; Wang, Y.-M. Stereodivergent Alkyne Hydrofluorination Using Protic Tetrafluoroborates as Tunable Reagents. *Angew. Chem. Int. Ed.* **2020**, *59*, 16651–16660. DOI: 10.1002/anie.202006278.

(3) Stoll, S.; Schweiger, A. EasySpin, a comprehensive software package for spectral simulation and analysis in EPR. *J. Magn. Reson.* **2006**, *178*, 42–55. DOI: 10.1016/j.jmr.2005.08.013.

(4) Bill, E. *julX: Program for Simulation of Molecular Magnetic Data*, **2008**.

(5) Kahn, O. *Molecular Magnetism, VCH Publ*; Inc.: New York, NY, USA, **1993**.

(6) Krejčik, M.; Danćk, M.; Hartl, F. Simple Construction of an Infrared Optically Transparent Thin-Layer Electrochemical Cell: Applications to the Redox Reactions of Ferrocene, $Mn_2(CO)_{10}$ and $Mn(CO)_3$(3,5-di-t-butyl-catecholate). *J. Electroanal. Chem. Interfacial Electrochem.* **1991**, *317*, 179−187.

(7) Grimme, S.; Hansen, A.; Ehlert, S.; Mewes, J.-M. r2SCAN-3c: A "Swiss army knife" composite electronic-structure method. *J. Chem. Phys.* **2021**, *154*, 64103. DOI: 10.1063/5.0040021.

(8) Caldeweyher, E.; Ehlert, S.; Hansen, A.; Neugebauer, H.; Spicher, S.; Bannwarth, C.; Grimme, S. A generally applicable atomic-charge dependent London dispersion correction. *J. Chem. Phys.* **2019**, *150*, 154122. DOI: 10.1063/1.5090222.

(9) Kruse, H.; Grimme, S. A geometrical correction for the inter- and intra-molecular basis set superposition error in Hartree-Fock and density functional theory calculations for large systems. *J. Chem. Phys.* **2012**, *136*, 154101. DOI: 10.1063/1.3700154.

(10) Neese, F. Software update: The ORCA program system—Version 5.0. *WIREs Comput Mol Sci* **2022**, *12*. DOI: 10.1002/wcms.1606.

(11) Neese, F.; Technical Directorship F. Wennmohs, with contributions from Aravena, D.; Atanasov, M., Auer, A. A.; Becker, U.; Bistoni, G.; Brehm, M.; Bykov, D.; Chilkuri, V. G.; Datta, D.; Dutta, A. K.; Ganyushin, D.; Garcia, M.; Guo, Y.; Hansen, A.; Helmich-Paris, B.; Huntington, L.; Izsák, R.; Kettner, M.; Kollmar, C.; Kossmann, S.; Krupiˇcka, M.; Lang, L.; Lechner, M.; Lenk, D.; Liakos, D. G.; Manganas, D.; Pantazis, D. A.; Papadopoulos, A.; Petrenko, T.; Pinski, P.; Pracht, P.; Reimann, C.; Retegan, M.; Riplinger, C.; Risthaus, T.; Roemelt, M.; Saitow, M.; Sandhöfer, B.; Schapiro, I.; Sen, A.; Sivalingam, K.; de Souza, B.; Stoychev, G.; Van den Heuvel, W.; Wezisla, B.; and with contributions from collaborators Kállay, M.; Grimme, S.; Valeev, E.; Chan, G.; Pittner, J.; Brehm, M.; Goerigk, L.; Åsgerisson, V.; Ungur, L. *ORCA version 5.0.3, an ab initio, DFT and semiempirical SCF-MO package* **2022**. https://orcaforum.cec.mpg.de/.

(12) Perdew, J. P.; Ernzerhof, M.; Burke, K. Rationale for mixing exact exchange with density functional approximations. *J. Chem. Phys.* **1996**, *105*, 9982–9985. DOI: 10.1063/1.472933.

(13) Adamo, C.; Barone, V. Toward reliable density functional methods without adjustable parameters: The PBE0 model. *J. Chem. Phys.* **1999**, *110*, 6158–6170. DOI: 10.1063/1.478522.

(14) Grimme, S.; Ehrlich, S.; Goerigk, L. Effect of the damping function in dispersion corrected density functional theory. *J. Comput. Chem.* **2011**, *32*, 1456–1465. DOI: 10.1002/jcc.21759. Published Online: Mar. 1, 2011.

(15) van Lenthe, E.; van der Avoird, A.; Wormer, P. E. S. Density functional calculations of molecular hyperfine interactions in the zero order regular approximation for relativistic effects. *J. Chem. Phys.***1998**, *108*, 4783–4796. DOI: 10.1063/1.475889.

(16) van Wüllen, C. Molecular density functional calculations in the regular relativistic approximation: Method, application to coinage metal diatomics, hydrides, fluorides and chlorides, and comparison with first-order relativistic calculations. *J. Chem. Phys.* **1998**, *109*, 392–399. DOI: 10.1063/1.476576.

(17) Neese, F. Efficient and accurate approximations to the molecular spin-orbit coupling operator and their use in molecular g-tensor calculations. *J. Chem. Phys.* **2005**, *122*, 34107. DOI: 10.1063/1.1829047.

(18) Weigend, F.; Ahlrichs, R. Balanced basis sets of split valence, triple zeta valence and quadruple zeta valence quality for H to Rn: Design and assessment of accuracy.*PCCP* **2005**, *7*, 3297–3305. DOI: 10.1039/B508541A.

(19) Weigend, F. Accurate Coulomb-fitting basis sets for H to Rn. *PCCP* **2006**, *8*, 1057–1065. DOI: 10.1039/B515623H.

(20) Pantazis, D. A.; Neese, F. All-electron basis sets for heavy elements. *WIREs Comput Mol Sci* **2014**, *4*, 363–374. DOI: 10.1002/wcms.1177.

(21) Neese, F.; Wennmohs, F.; Hansen, A.; Becker, U. Efficient, approximate and parallel Hartree–Fock and hybrid DFT calculations. A 'chain-of-spheres' algorithm for the Hartree–Fock exchange. *Chemical Physics* **2009**, *356*, 98–109. DOI: 10.1016/j.chemphys.2008.10.036.

(22) Glendening, E. D.; Badenhoop, J. K.; Reed, A. E.; Carpenter, J. E.; Bohmann, J. A.; Morales, C. M.; Karafiloglu, P.; Landis, C. R.; Weinhold, F. *NATURAL BOND ORBITAL 7.0*. https://nbo7.chem.wisc.edu/ (accessed 2024-07-03).

(23) Sheldrick, G. M. SHELXT - integrated space-group and crystal-structure determination. *Acta crystallographica. Section A, Foundations and advances* **2015**, *71* (Pt 1), 3–8. DOI: 10.1107/S2053273314026370.

(24) Sheldrick, G. M. Crystal structure refinement with SHELXL. *Acta Cryst C* **2015**, *71* (Pt 1), 3–8. DOI: 10.1107/S2053229614024218.

(25) *X-RED*; STOE & CIE GmbH: Darmstadt, Germany, **2002**.

(26) *SADABS*; BRUKER AXS GmbH: Karlsruhe, Germany, **2016**.

(27) Menges, F. *Spectragryph - optical spectroscopy software*, **2016-2019**. http://www.effemm2.de/spectragryph/.
